# Circulating CD38 shows stronger association with incident heart failure in women despite lower levels: sex differences and kidney-function context

**DOI:** 10.1186/s13293-026-00924-7

**Published:** 2026-06-04

**Authors:** Richard Mprah, Jeremiah Ong’Achwa Machuki, Prosperl Ivette Wowui, Diana Chulu, Yixuan Lei, Anastasia Wemaatu Lamawura Kanoseh, Manuella Quaye, Parfait Botolo Sakava, Simon Kumah Yalley, Joshua Nii Yemo Quarshie, Saffia Shaheen, Tao Dong, Hong Sun

**Affiliations:** Department of Physiology, Xuzhou Medical University, 209 Tongshan Road, Xuzhou, 221004 Jiangsu China

**Keywords:** CD38, Heart Failure, Sex Differences, NAD+, Biomarkers, Metabolism, Kidney Function, Preclinical Model

## Abstract

**Background:**

CD38, an NADase implicated in aging and metabolic stress, promotes cardiac dysfunction via NAD⁺ depletion. Whether circulating CD38 is associated with incident heart failure (HF), whether this association differs by sex, and how it relates to kidney-function context remain unclear.

**Methods:**

We measured plasma CD38 in 42,584 UK Biobank participants (1,659 incident HF events) and evaluated its association with HF using sequential Cox models, attenuation analyses, competing-risk models, and incremental prediction metrics. Complementary mouse studies employed chronic β-adrenergic stress (isoproterenol) to investigate sex-specific responses and test the effects of CD38 inhibition (78c), TGF-β1 inhibition (SB431542), and their combination.

**Results:**

CD38 was associated with incident HF in models adjusting for traditional risk factors (M1: HR per SD 1.25, 95% CI 1.18–1.32), with complete attenuation after eGFR adjustment. Despite higher CD38 levels in men, the association was stronger in women (M1: HR 1.39 vs. 1.17 per SD; sex interaction *P* = 0.004). After eGFR adjustment, the association remained positive in women (M3: HR 1.17) but became inverse in men (M3: HR 0.90). The CD38-HF association showed substantial attenuation after adjustment for NT-proBNP, ANGPT2, and IL-6, consistent with shared statistical variance with downstream biomarker burden rather than causal mediation. Fine–Gray competing-risk models (3,212 deaths without HF) produced similar results. In mice, isoproterenol induced cardiac remodeling and systolic impairment in both sexes and elicited greater metabolic and renal stress responses in females despite their higher baseline cardiac NAD⁺/ATP levels. CD38 inhibition restored cardiac NAD⁺/ATP and reduced renal stress markers, with numerically greater reductions in females, while TGF-β1 inhibition reduced fibrotic signaling. Combined inhibition produced complementary effects across metabolic and fibrotic readouts.

**Conclusions:**

Circulating CD38 is not an independent predictor of HF after accounting for kidney function and downstream biomarker burden, but appears to reflect a broader cardiometabolic-renal stress context. Despite lower circulating levels, CD38 showed a stronger association with HF in women than in men. Proof-of-concept mouse experiments showed that CD38 inhibition preserved cardiac NAD⁺/ATP and reduced renal stress markers, while TGF-β pathway inhibition reduced fibrotic signaling, providing mechanistic support for further sex-aware HF research.

**Graphical abstract:**

**Figure:**
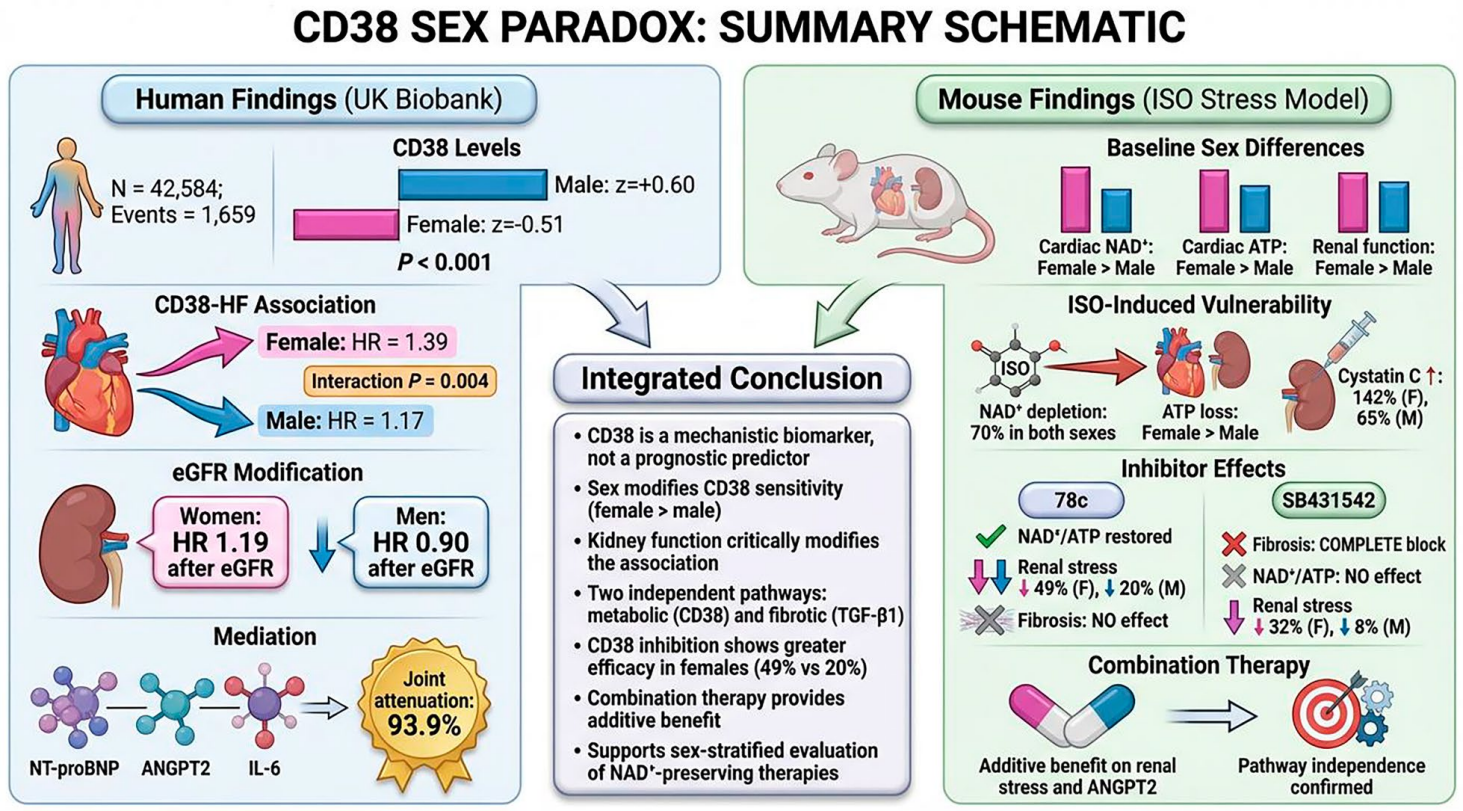

**Supplementary Information:**

The online version contains supplementary material available at 10.1186/s13293-026-00924-7.

## Plain language summary

Women and men can develop heart failure through different biological pathways. CD38 is a protein in blood that breaks down NAD⁺, a molecule that cells use to make energy. We asked whether blood CD38 levels are linked to future heart failure, and whether this link differs by sex and kidney function. We studied 42,584 UK Biobank participants without heart failure at baseline and followed them for new-onset heart failure (1,659 events). We tested whether CD38 predicted heart failure risk and whether the association differed between women and men and across levels of kidney function. We also used a mouse model of long-term stress on the heart to examine sex-specific responses and to test whether blocking CD38 could protect the heart and kidney-related stress signals. Higher CD38 levels were associated with a higher risk of developing heart failure. Even though men had higher CD38 levels overall, the relationship between CD38 and heart failure risk was stronger in women. Kidney function also mattered: in women, higher CD38 was more strongly linked to risk as kidney function declined, while in men, the pattern differed when kidney function was very good. In mice, heart stress reduced heart energy molecules and triggered stronger kidney-stress responses in females. Blocking CD38 restored cardiac energy molecules in both sexes and reduced kidney-stress signals more in females. These findings suggest CD38-related energy pathways may contribute to heart failure risk differently in women and men, and that kidney function should be considered when evaluating NAD⁺-preserving strategies.

**Supplementary Information:**

The online version contains supplementary material available at 10.1186/s13293-026-00924-7.

## Highlights

- In 42,584 UK Biobank participants followed for 12.8 years (1,659 incident events), higher circulating CD38 was associated with a higher risk of incident heart failure
- The CD38–heart failure association differed by sex: despite higher CD38 levels in men, the risk association per unit increase was stronger in women
- Kidney-function strata suggested possible heterogeneity in this sex difference, with distinct CD38 association patterns across eGFR categories
- Most of the CD38 association was explained by established downstream biomarkers, and adding CD38 did not materially improve risk prediction beyond standard clinical and biomarker models
- In male and female mice exposed to chronic β-adrenergic stress, CD38 inhibition restored cardiac NAD⁺ and ATP and reduced kidney-linked stress responses with greater benefit in females, providing mechanistic support for a sex-differential metabolic stress response

**Supplementary Information:**

The online version contains supplementary material available at 10.1186/s13293-026-00924-7.

## Introduction

Despite major advances in prevention and treatment, heart failure (HF) remains a leading cause of morbidity and mortality worldwide and continues to show important sex differences that are not fully understood [1]. Women and men differ not only in lifetime HF risk, but also in typical HF antecedents, ventricular remodeling patterns, and responses to established therapies [1, 2]. Current biomarkers such as NT-proBNP improve diagnosis and risk stratification, but they largely reflect established cardiac stress and injury rather than upstream biological processes that may shape vulnerability earlier in the disease course [3, 4]. Identifying upstream pathways that differ between women and men may therefore help refine sex-informed strategies for HF prevention and treatment.

Sex-specific biomarker behavior is increasingly recognized as clinically relevant in cardiovascular medicine. A well-known example is cardiac troponin: women typically have lower circulating concentrations than men, yet troponin increases may carry important prognostic implications and have contributed to the adoption of sex-specific diagnostic thresholds in clinical practice [5–7]. More broadly, a systematic analysis of 86 cardiovascular biomarkers showed that most differ by sex in ways that may influence risk assessment [8]. However, sex-stratified associations remain underexamined for many emerging biomarkers. The long-standing male bias in preclinical cardiovascular research reinforces this gap. Although current policy emphasizes sex as a biological variable [9], many mechanistic studies still rely predominantly on male animals, limiting understanding of female biology and potentially obscuring sex-specific therapeutic responses [10].

CD38 is a multifunctional ectoenzyme and the principal mammalian NADase, consuming the essential metabolic cofactor NAD⁺ and thereby influencing cellular energy balance, mitochondrial function, and stress adaptation [11]. In experimental models of pressure overload, ischemia-reperfusion injury, aging, and metabolic stress, CD38 upregulation has been linked to NAD⁺ depletion, impaired mitochondrial function, and adverse cardiac remodeling [12–14]. Pharmacologic or genetic suppression of CD38 can restore NAD⁺ availability and improve cardiac phenotypes in some settings, supporting CD38 as a plausible upstream pathway of cardiovascular vulnerability [14]. At the same time, most preclinical CD38 studies in cardiovascular disease have been conducted in male animals. The smaller body of work that has included both sexes suggests that CD38-related biology may be sex-dependent, with sex-specific patterns reported in cardiac hypertrophy, hormonal modulation of functional benefit, and myocarditis models [15–17]. However, no prospective human study has directly tested whether circulating CD38 shows different associations with incident HF in women and men.

TGF-β1 provides an informative comparison pathway. Unlike CD38, which is closely linked to cellular energetics and NAD⁺ metabolism, TGF-β1 is a well-established mediator of fibrotic remodeling [18]. Examining both biomarkers in parallel, therefore, offers an opportunity to distinguish metabolic-stress and fibrosis-related components of HF risk. In addition, circulating CD38 is strongly inversely correlated with glomerular filtration rate [19], raising the possibility that kidney function may influence the CD38-HF association through biological stress pathways, altered biomarker handling, confounding by cardiorenal disease burden, or some combination of these mechanisms. Whether kidney function modifies the relationship between CD38 and HF, and whether any such modification differs by sex, has not been established.

We therefore hypothesized that circulating CD38 marks an upstream metabolic-stress pathway relevant to incident HF, that its association with HF differs by sex, and that kidney-function context materially influences this relationship. We further hypothesized that CD38-linked biology would exhibit a distinct downstream profile compared with TGF-β1-mediated fibrotic signaling. To test these questions, we used a two-phase translational strategy. First, we examined plasma CD38 and TGF-β1 in relation to incident HF over 12.8 years in 42,584 UK Biobank participants, with explicit evaluation of sex differences, kidney-function context, and attenuation after adjustment for established downstream biomarkers. Second, we used a chronic β-adrenergic stress model in male and female mice to address the male-only gap in preclinical CD38 research and to experimentally probe the effects of CD38 inhibition (78c), TGF-β receptor I inhibition (SB431542), and their combination on metabolic, fibrotic, and renal stress-related readouts. A schematic overview of the hypothesized relationships among CD38, sex, kidney function, and TGF-β1-related fibrosis is provided in Supplementary Fig. S2.

## Materials and methods

### Study population and data sources

#### UK biobank cohort

**Fig. 1:**
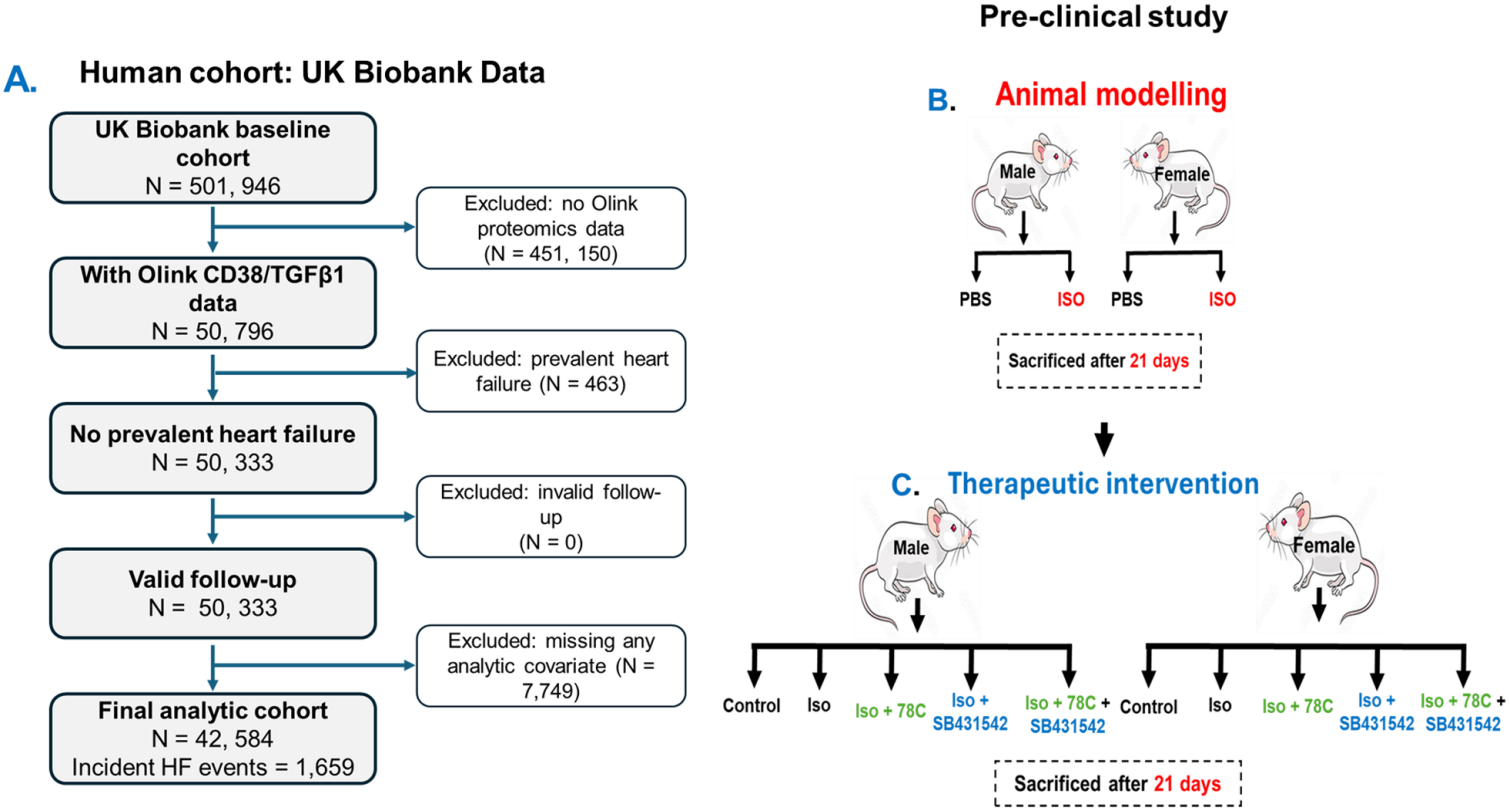
Study design. (**A**) Participant flow diagram for the UK Biobank analytic cohort. (**B**) Phase 1 murine protocol: male and female FVB mice received vehicle or isoproterenol (ISO; 2.5 mg/kg/day, subcutaneous) for 21 days; transthoracic echocardiography was performed at day 21 in a subset of animals (*n* = 5 per group) using a Silicon Wave 60 ultrasound system. (**C**) Phase 2 inhibitor protocol: mice received ISO alone or in combination with the CD38 inhibitor 78c (10 mg/kg/day, intraperitoneal), the TGF-β receptor I inhibitor SB431542 (10 mg/kg/day, intraperitoneal), or both for 21 days

This prospective cohort study utilized data from the UK Biobank, a large-scale population-based study that enrolled approximately 500,000 participants aged 40–69 years across the United Kingdom between 2006 and 2010. For the present analysis, we included participants with available proteomic measurements of CD38 and transforming growth factor-β1 (TGF-β1) from the Olink Explore 3072 platform (*n* = 50,796). Participants were excluded sequentially as follows: 463 with prevalent heart failure at baseline (ICD-10 I50 recorded on or before the baseline assessment date); and 7,749 with missing values in one or more analytic covariates (primarily C-reactive protein, eGFR, systolic blood pressure, and BMI), yielding a final analytic cohort of 42,584 participants (Fig. 1A). All 16 analytic variables had 0% missingness in the final dataset, confirming complete-case analysis. The complete-case approach may introduce selection bias if missingness is related to both the exposure and outcome; this is acknowledged as a limitation. Multiple imputation was not performed because the primary source of missingness (the absence of Olink assay data in the broader UK Biobank cohort) is not recoverable through imputation.

#### Exposure assessment

The primary exposures were plasma CD38 and TGF-β1 concentrations measured using the Olink Explore 3072 proximity extension assay platform. Protein levels were expressed as Normalized Protein Expression (NPX) values, which are log2-transformed and normalized for technical variation. For all regression analyses, protein concentrations were standardized to z-scores (mean = 0, SD = 1) to facilitate comparison of effect sizes across biomarkers.

#### Outcome ascertainment

The primary outcome was incident heart failure, identified through linked hospital inpatient records and death registries using International Classification of Diseases, Tenth Revision (ICD-10) code I50. Participants were followed from their baseline assessment date until the first occurrence of heart failure, death, or the censoring date (December 31, 2021), whichever occurred first.

#### Covariate assessment

Baseline demographic and clinical characteristics were obtained from questionnaires, physical measurements, and linked health records. Covariates included age, sex, body mass index (BMI), systolic blood pressure, smoking and alcohol consumption status, and prevalent comorbidities (hypertension, diabetes mellitus, hyperlipidemia). Additional circulating biomarkers included NT-proBNP, IL-6, ANGPT2, and CRP. Medication use at baseline was determined from self-reported treatment codes and included cardiovascular medications (statins, ACEi/ARB, beta-blockers, calcium channel blockers, diuretics) and metformin. Complete definitions of covariates are provided in the Supplementary Methods.

#### Hormonal and reproductive status

In female participants, menopausal status was classified using a validated age-based proxy: women aged < 50 years at baseline were classified as premenopausal and women aged ≥ 50 years as postmenopausal, consistent with the median age of natural menopause in UK women (approximately 51 years). This approach did not incorporate self-reported menopause date, hormone replacement therapy (HRT) use, or surgical menopause history. Women aged 45–60 years were included in menopausal status analyses to minimise age confounding at the classification boundary. The limitation of this proxy, specifically that women aged ≥ 50 using HRT or those with premature menopause may be misclassified, is acknowledged; such misclassification could attenuate or otherwise distort subgroup differences by including some hormonally post-menopausal women in the premenopausal group. Circulating hormone concentrations were measured at baseline in both sexes, including estradiol (women only), testosterone, sex hormone-binding globulin (SHBG), and insulin-like growth factor-1 (IGF-1). Free androgen index (FAI) was calculated as testosterone/SHBG × 100. Hormone values were log-transformed where appropriate and standardised to z-scores for analysis. Correlations between CD38 and each hormone were examined separately in men and women using Pearson’s correlation.

#### Kidney function assessment

Estimated glomerular filtration rate (eGFR) was calculated using the CKD-EPI 2021 combined creatinine-cystatin C equation, incorporating serum creatinine (µmol/L, converted to mg/dL) and serum cystatin C (mg/L) measured at baseline, together with age and sex. This combined equation was selected as it provides superior accuracy compared with creatinine-only estimates, and both analytes were available in the UK Biobank Olink proteomics subsample. For analyses examining kidney function as an effect modifier, eGFR was categorized into three clinically relevant groups: preserved kidney function (eGFR ≥ 90 mL/min/1.73 m²), mildly to moderately reduced kidney function (eGFR 60–89 mL/min/1.73 m²), and moderately to severely reduced kidney function (eGFR < 60 mL/min/1.73 m²). Continuous eGFR was used in correlation analyses with biomarkers and as an adjustment covariate in sensitivity analyses.

### Statistical analysis

#### Descriptive statistics and primary survival analysis

Baseline characteristics were summarized using means with standard deviations for continuous variables and frequencies with percentages for categorical variables. Differences between participants who developed incident heart failure and those who remained heart failure-free were assessed using two-sample t-tests for continuous variables and chi-square tests for categorical variables. The association between biomarkers (CD38, TGF-β1) and incident heart failure was assessed using Cox proportional hazards regression models, presented as a prespecified sequential framework of four models. Model 1 (M1) adjusted for age (continuous, z-score), sex, body mass index (continuous, z-score), prevalent hypertension (binary), and prevalent diabetes (binary). Model 2 (M2) added systolic blood pressure (z-score), smoking status (current, former), alcohol use (current drinker, binary), hyperlipidaemia, and baseline medication use (statins, ACE inhibitors/angiotensin receptor blockers, beta-blockers, diuretics, calcium channel blockers, and metformin, each coded as binary from self-reported medication data). Model 3 (M3) further added eGFR. Model 4 (M4) further added downstream biomarkers (NT-proBNP, ANGPT2, IL-6). This sequential framework was prespecified to evaluate whether the CD38-HF association reflects confounding by traditional risk factors and medications (M1→M2), kidney function (M2→M3), or downstream cardiometabolic stress (M3→M4). Hazard ratios (HRs) and 95% confidence intervals (CIs) were estimated for each 1-standard deviation increment in standardized biomarker levels. For quartile-based analyses, CD38 was categorized into quartiles with the lowest quartile (Q1) as the reference group.

#### Sex-stratified analysis and sex-specific effect modification

Sex-stratified Cox proportional hazards models were fitted separately in males and females in two stages: (i) an unadjusted model including CD38 alone, and (ii) a fully adjusted model including CD38, TGF-β1, age, BMI, systolic blood pressure, eGFR, hypertension, diabetes, hyperlipidaemia, smoking status, and the downstream biomarkers NT-proBNP, IL-6, and ANGPT2. Statistical interaction between CD38 and sex was assessed by including a multiplicative interaction term (z_CD38 × sex) in a Cox model adjusted for TGF-β1, age, BMI, systolic blood pressure, eGFR, hypertension, diabetes, hyperlipidaemia, and smoking status, with significance evaluated from the model coefficient for the interaction term (z-test). To address potential scaling artifacts arising from whole-cohort standardisation, a sensitivity analysis was performed in which CD38 was standardised separately within males and females before Cox regression.

In an exploratory analysis, the CD38–HF association was examined according to menopausal status in women aged 45–60 years (*n* = 11,892). Menopausal status was classified using an age-based proxy derived from UK Biobank Field 21,003 (age at recruitment): women aged < 50 years were classified as premenopausal and those aged ≥ 50 years as postmenopausal [20–22]. This classification does not incorporate self-reported menopause date, surgical menopause, or hormone replacement therapy use, and potential misclassification is acknowledged as a limitation. Cox models were fitted, including CD38 (per 1-SD), menopausal status, their multiplicative interaction term, and adjustment for age, BMI, hypertension, and diabetes. Given the small number of premenopausal HF events (*n* = 20), this analysis is considered hypothesis-generating and requires replication in cohorts with confirmed menopausal status.

#### Three-way interaction analysis: biomarker × sex ×Kidney function

To test whether kidney function modifies the sex-specific associations between biomarkers and heart failure, we performed three-way interaction analyses. Participants were stratified into six groups based on sex (male or female) and eGFR category (≥ 90, 60–89, or < 60 mL/min/1.73 m²). Cox models were fit separately within each stratum using the base adjustment model. The significance of the three-way interaction was assessed using likelihood ratio tests. To quantify the degree of attenuation by eGFR adjustment, we calculated: % Attenuation = [(HR_without_eGFR - HR_with_eGFR)/(HR_without_eGFR − 1)] × 100%, where values exceeding 100% indicate complete attenuation with reversal of effect direction.

#### Biomarker attenuation analysis

To characterize the statistical relationship between CD38/TGF-β1 and established downstream biomarkers of cardiac stress (NT-proBNP), vascular dysfunction (ANGPT2), and systemic inflammation (IL-6), we conducted a biomarker attenuation analysis and a supplementary path analysis. Because all biomarkers were measured at the same baseline assessment, the sequential adjustment approach cannot establish temporal ordering or causal direction; these analyses are interpreted as exploratory characterisations of shared statistical variance rather than evidence of causal mediation. For each biomarker pair, we estimated: Path a (exposure → downstream biomarker association); Path b (downstream biomarker → outcome association, adjusting for exposure); Total effect (β_total, without downstream biomarker adjustment); and Direct effect (β_direct, with downstream biomarker adjustment). The proportion of association explained by shared variance with each downstream biomarker was calculated as: % Attenuated = [(β_total − β_direct)/β_total] × 100%. Individual biomarker contributions were assessed separately; joint attenuation was assessed by simultaneously including all three biomarkers. A supplementary path analysis (SEM-like sequential regression) was performed to provide directional estimates: the total indirect effect of CD38 through IL-6, ANGPT2, and NT-proBNP was estimated as the product of standardised path coefficients. All analyses were performed overall and stratified by sex. A proportion attenuated exceeding 100% indicates a statistical suppression effect, whereby adjustment for correlated downstream biomarkers unmasks an underlying inverse direct association; this pattern is consistent with the sex-stratified sequential Cox model results and does not imply causal mediation. Because all biomarkers were measured at the same baseline assessment, these analyses are interpreted as descriptive attenuation analyses rather than causal mediation models.

#### Statistical software

All statistical analyses were performed using R version 4.3.0. Cox proportional hazards models were fit using the survival package, discrimination metrics were calculated using the timeROC package, and spline analyses were performed using the rms package. Two-sided p-values < 0.05 were considered statistically significant. Additional details on subgroup analyses, sensitivity analyses, model diagnostics, and predictive discrimination are provided in Supplementary Methods.

### Preclinical mechanistic studies

#### Animals and experimental design

Male and female FVB mice aged 8–10 weeks were obtained from the institutional animal facility and housed under standard conditions. The study was conducted in two phases. In Phase 1, mice of both sexes received either vehicle control (PBS) or isoproterenol (ISO, 2.5 mg/kg/day, subcutaneous) for 21 days to establish baseline sex differences and characterize sex-specific responses to β-adrenergic stress (Fig. 1B). In Phase 2, separate cohorts were assigned to test pathway-specific inhibition: control, ISO alone, ISO plus CD38 inhibitor 78c (10 mg/kg/day, intraperitoneal), ISO plus TGF-β receptor I inhibitor SB431542 (10 mg/kg/day, intraperitoneal), or ISO plus combined treatment (Fig. 1C). The treatment dosages were selected according to previous reports [14], [23–25]. Pharmacological inhibitors were administered simultaneously with ISO for 21 days. Sample sizes were *n* = 6–7 mice per group for metabolite and plasma biomarker measurements, and *n* = 3 per group for Western blot analyses in Phase 1. For echocardiographic assessment, *n* = 5 mice per group (Phase 1 only) underwent imaging at baseline and at day 21. All procedures were approved under Protocol # xz11-12540.

#### Echocardiography

Transthoracic echocardiography was performed at day 21 of the ISO or vehicle protocol in Phase 1 animals (*n* = 5 per group) under light isoflurane anaesthesia (1.5–2.0.5.0%), as previously described [26], using a Silicon Wave 60 ultrasound system (KOLO Medical). M-mode images were acquired from the parasternal short-axis view at the level of the papillary muscles. All measurements were performed by an observer blinded to group allocation, with a minimum of three consecutive cardiac cycles averaged per animal. Two-way ANOVA (sex × treatment) was used to test for group differences and interactions.

#### Tissue collection and biochemical assays

At study termination (day 21), mice were anesthetized with esketamine. Blood was centrifuged, and plasma was stored at −80 °C. Hearts from Phase 2 animals were snap-frozen in liquid nitrogen and sectioned at 5 μm for Masson’s trichrome staining (G1340; Solarbio Life Sciences). Collagen volume fraction (CVF) was quantified as the ratio of collagen-positive (blue) area to total myocardial area using ImageJ (NIH, version 1.53) on five non-overlapping fields per section, averaged per animal. Hearts from Phase 1 animals were snap-frozen for biochemical assays. Cardiac NAD⁺/NADH levels were quantified using the Coenzyme I NAD/NADH Assay Kit (Solarbio, catalog # BC0310), and ATP levels were measured using the Enhanced ATP Assay Kit (Beyotime, catalog # S0026). Metabolite concentrations were normalized to tissue protein content and expressed as relative units normalized to sex-matched controls. Plasma concentrations of CD38, TGF-β1, ANGPT2, pSMAD2/3, and cystatin C were measured by ELISA (kit details in Supplementary Methods). All samples were run in duplicate with intra-assay and inter-assay coefficients of variation less than 10% and 15%, respectively.

Renal gene expression was assessed in Phase 1 kidney cortex samples by quantitative reverse-transcription PCR (qRT-PCR). Total RNA was extracted from frozen kidney cortex tissue using TRIzol™ Reagent (Invitrogen™; catalog no. 15596026) according to the manufacturer’s protocol. Complementary DNA (cDNA) was generated using a reverse transcription kit (FSQ107; Toyobo), and qPCR analysis was performed using SYBR Green Master Mix (Q111-02; Vazyme) according to the manufacturer’s instructions. Primer sequences were as follows: mouse Cd38 forward 5′-GGTCCAAGTGATGCTCAATGGG-3′, reverse 5′-AGCTCCTTCGATGTCGTGCATC-3′ (OriGene Technologies, Inc, USA; MP201925); β-actin forward 5′-CATTGCTGACAGGATGCAGAAGG-3′, reverse 5′-TGCTGGAAGGTGGACAGTGAGG-3′. Relative expression was calculated using the ΔΔCt method with β-actin as the reference gene. Two-way ANOVA (sex × treatment) was used to assess group differences, followed by Tukey’s post-hoc test.

#### Statistical analysis for preclinical studies

Data are presented as mean ± SEM. For baseline sex differences, unpaired Student’s t-tests were used. For treatment effects within each sex, a one-way ANOVA was performed, followed by Tukey’s post hoc test. To assess sex-specific treatment responses, a two-way ANOVA with sex and treatment as factors was performed, followed by Sidak’s post hoc test when the interaction was significant. Statistical significance was set at *P* < 0.05. Effect sizes for ANOVA effects are reported as eta squared (η² = SS_effect/SS_total); complete ANOVA results, including sums of squares, F-statistics, degrees of freedom, and η² for all outcomes, are provided in Supplementary Table 8. All analyses were performed using GraphPad Prism version 9.0.

## Results

### Cohort characteristics and incident heart failure

The cohort comprised 42,584 participants (54.2% female; mean age 56.75 ± 8.22 years; BMI 27.42 ± 4.74 kg/m²). At baseline, 42.1% had hypertension, 2.1% diabetes, and 26.9% hyperlipidemia. Overall, 31.6% used cardiovascular medications (statins 16.0%, ACEi/ARB 13.8%, beta-blockers 6.5%, CCBs 6.3%, diuretics 7.4%), and 19.5% had polypharmacy (Table 1). Over a median follow-up of 12.83 years (IQR 12.18–13.52), 1,659 participants (3.9%) developed incident heart failure (3.07 per 1,000 person-years). Follow-up was shorter among HF cases than non-cases (8.29 vs. 12.87 years; *P* < 0.001), with 24.2% of events occurring within 5 years. Compared with participants who remained HF-free, those who developed HF were older, more often male, and had higher BMI and systolic blood pressure, with markedly higher prevalence of hypertension, diabetes, and hyperlipidemia (all *P* ≤ 0.023) (Table 1). HF cases also had lower LDL- and HDL-cholesterol, higher HbA1c, and substantially greater use of all cardiovascular medication classes (all *P* < 0.001) (Table 1). Participants who developed HF had higher baseline CD38, TGF-β1, NT-proBNP, IL-6, ANGPT2, and CRP (all *P* < 0.001) (Supplementary Table 1), though these biomarkers exhibited sex-specific patterns in their associations with HF.

**Table 1:** Baseline Characteristics of Study Participants by Incident Heart Failure Status

Characteristic	Overall (*N* = 42,584)	No HF (*N* = 40,984)	HF (*N* = 1,659)	*P*-value
**Demographics**				
Age, years	56.75 ± 8.22	56.52 ± 8.21	62.45 ± 6.22	< 0.001
Female sex, n (%)	23,122 (54.2)	22,460 (54.8)	662 (39.9)	< 0.001
BMI, kg/m²	27.42 ± 4.74	27.33 ± 4.67	29.64 ± 5.71	< 0.001
Systolic BP, mmHg	137.73 ± 18.63	137.48 ± 18.53	144.02 ± 20.07	< 0.001
Diastolic BP, mmHg	82.15 ± 10.15	82.16 ± 10.10	82.03 ± 11.15	0.6
**Comorbidities**,** n (%)**				
Hypertension	17,949 (42.1)	16,569 (40.4)	1,380 (83.1)	< 0.001
Diabetes mellitus	908 (2.1)	859 (2.1)	49 (3.0)	0.023
Hyperlipidemia	11,486 (26.9)	10,560 (25.8)	926 (55.8)	< 0.001
**Lifestyle Factors**,** n (%)**				
Current smoker	4,476 (10.5)	4,220 (10.3)	256 (15.4)	< 0.001
Former smoker	14,816 (34.7)	14,081 (34.4)	735 (44.3)	< 0.001
**Lipid Profile**				
Total cholesterol, mmol/L	5.67 ± 1.15	5.68 ± 1.15	5.22 ± 1.25	< 0.001
LDL-C, mmol/L	3.54 ± 0.87	3.55 ± 0.87	3.24 ± 0.94	< 0.001
HDL-C, mmol/L	1.45 ± 0.38	1.45 ± 0.38	1.32 ± 0.39	< 0.001
Triglycerides, mmol/L	1.47 [1.04, 2.13]	1.47 [1.04, 2.12]	1.65 [1.16, 2.33]	< 0.001
**Metabolic Markers**				
HbA1c, mmol/mol	35.30 [32.80, 38.00]	35.20 [32.80, 37.90]	37.40 [34.30, 41.00]	< 0.001
Urate, µmol/L	303.3 [251.4, 361.8]	301.9 [250.3, 360.1]	343.8 [288.7, 408.0]	< 0.001
Albumin, g/L	45.17 ± 2.64	45.21 ± 2.62	44.21 ± 2.91	< 0.001
**Medications**,** n (%)**				
Statins	6,829 (16.0)	6,179 (15.1)	650 (39.2)	< 0.001
ACEi/ARB	5,904 (13.8)	5,285 (12.9)	619 (37.3)	< 0.001
Beta-blockers	2,770 (6.5)	2,423 (5.9)	347 (20.9)	< 0.001
CCB	2,701 (6.3)	2,432 (5.9)	269 (16.2)	< 0.001
Diuretics	3,156 (7.4)	2,849 (7.0)	307 (18.5)	< 0.001
Antiplatelets	6,259 (14.7)	5,634 (13.7)	625 (37.7)	< 0.001
Any CV medication	13,458 (31.6)	12,384 (30.2)	1,074 (64.7)	< 0.001
Polypharmacy (≥ 5 meds)	8,321 (19.5)	7,467 (18.2)	854 (51.4)	< 0.001
**Follow-up**				
Duration, years	12.83 [12.18, 13.52]	12.87 [12.24, 13.56]	8.29 [5.13, 10.80]	< 0.001

Data are presented as mean ± SD, median [IQR], or n (%). P-values from Student’s t-test, Wilcoxon rank-sum test, or chi-square test comparing participants who developed heart failure versus those who remained heart failure-free during follow-up. Abbreviations: ACEi, angiotensin-converting enzyme inhibitor; ARB, angiotensin receptor blocker; BMI, body mass index; BP, blood pressure; CCB, calcium channel blocker; CV, cardiovascular; HbA1c, glycated hemoglobin; HDL-C, high-density lipoprotein cholesterol; HF, heart failure; LDL-C, low-density lipoprotein cholesterol

### Association of CD38 and TGF-β1 with incident heart

**Fig. 2:**
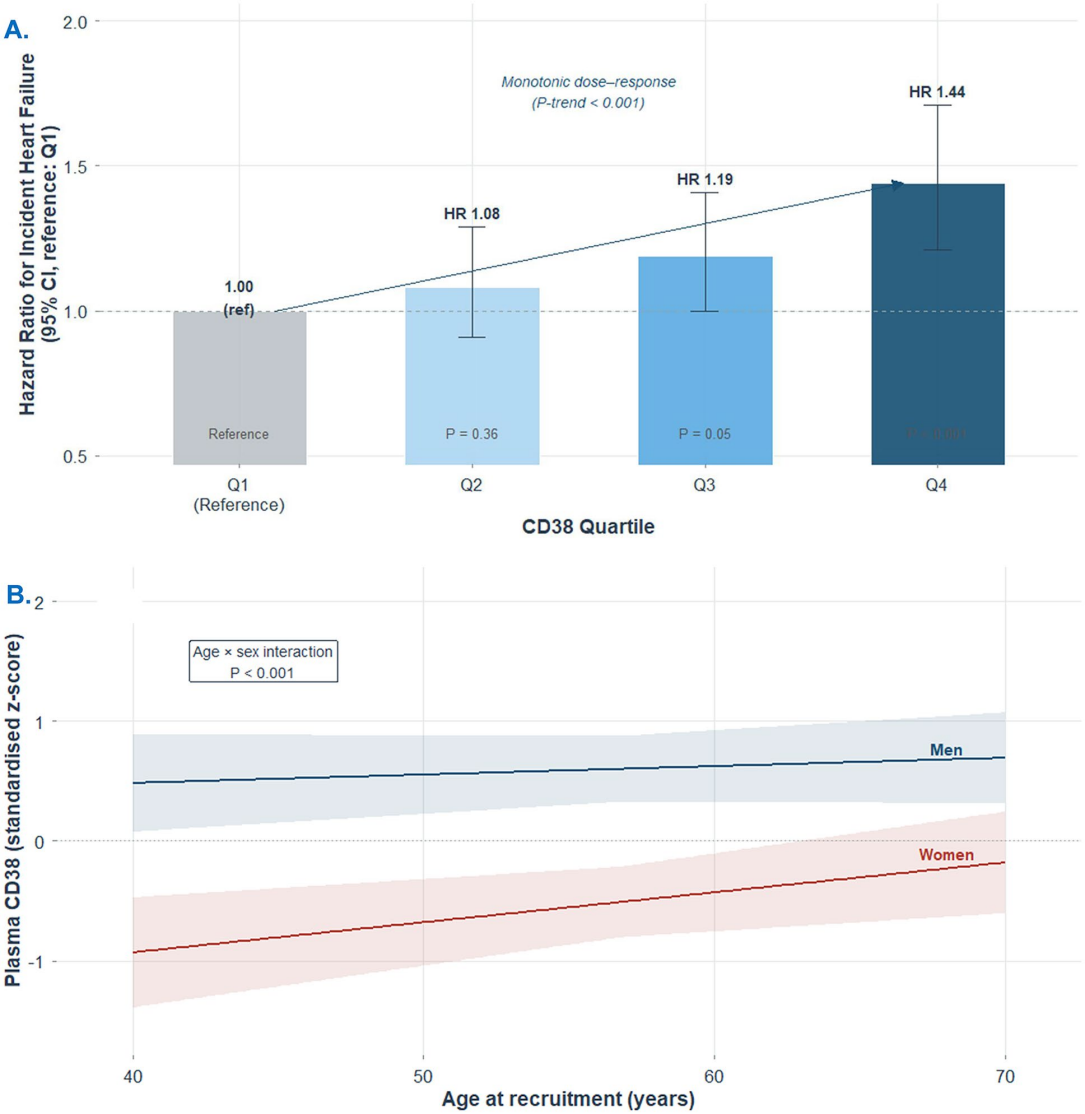

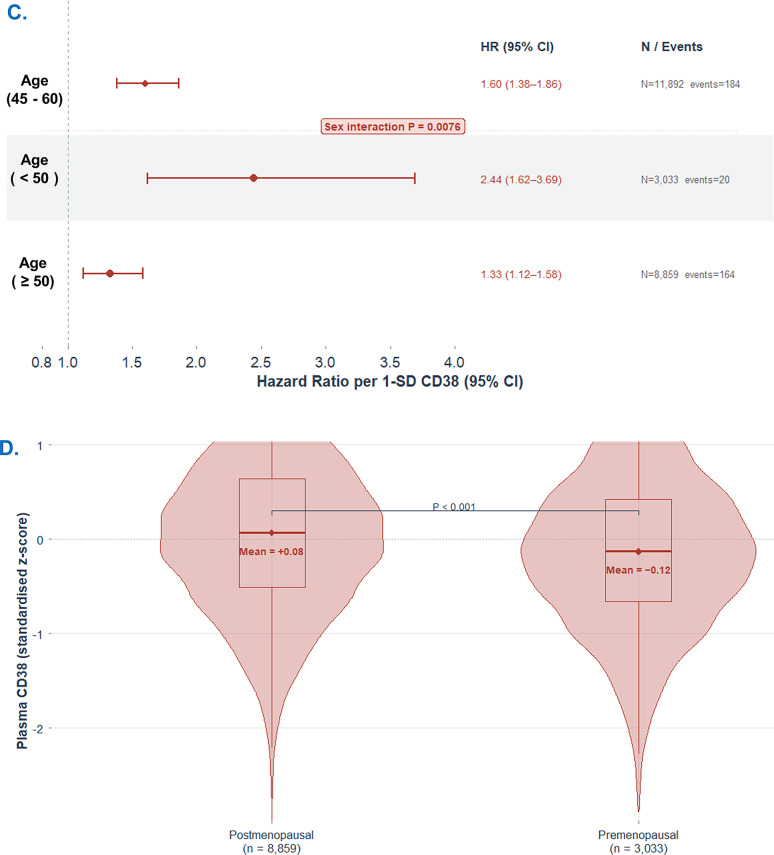
Plasma CD38 and incident heart failure. (**A**) Incident heart failure risk across quartiles of plasma CD38, with the lowest quartile (Q1) as the reference group. (**B**) Plasma CD38 by age in women and men. (**C**) Exploratory analysis: association of CD38 with incident heart failure in women stratified by menopausal status among participants aged 45–60 years (premenopausal HF events *n* = 20; results require replication). (**D**) Distribution of plasma CD38 according to menopausal status. In panel A, hazard ratios (HRs) and 95% confidence intervals (CIs) are shown for quartiles relative to Q1. In panel C, HRs and 95% CIs are shown per 1-standard deviation (SD) higher CD38. Women aged < 50 years were classified as premenopausal and those aged ≥ 50 years as postmenopausal. UK Biobank Cox models were adjusted for age, sex where applicable, body mass index, hypertension, and diabetes

In sequential Cox models (Table 2), CD38 was associated with incident heart failure in minimally adjusted analyses (M1: age, sex, BMI, hypertension, diabetes; HR per 1-SD 1.248, 95% CI 1.184–1.316, *P* < 0.001). The unadjusted association was stronger (HR 1.59, 95% CI 1.52–1.67), reflecting confounding by metabolic risk burden. A clear dose-response relationship was observed when quartiles analyzed CD38: compared to the lowest quartile (Q1), participants in Q2 (HR 1.08, 95% CI 0.91–1.29, *P* = 0.36), Q3 (HR 1.19, 95% CI 1.00–1.41, *P* = 0.05), and Q4 (HR 1.44, 95% CI 1.21–1.71, *P* < 0.001) showed progressively increased heart failure risk (Fig. 2A). Additional adjustment for SBP, smoking, alcohol, hyperlipidemia, and medication use (M2) produced modest attenuation (HR 1.212, 95% CI 1.149–1.278). Further adjustment for eGFR (M3) led to full statistical attenuation of the overall association (HR 0.989, 95% CI 0.923–1.059, *P* = 0.748), with the HR remaining near unity after additional adjustment for NT-proBNP, ANGPT2, and IL-6 (M4: HR 0.975, 95% CI 0.911–1.043). This sequential attenuation pattern was robust to substituting BMI with waist circumference and body fat percentage (Supplementary Table 5) and to a competing-risks analysis using the Fine–Gray subdistribution model, accounting for 3,212 deaths as competing events (Supplementary Table 6; overall HR 0.997, 95% CI 0.931–1.067). TGF-β1 was also associated with incident heart failure in minimally adjusted models (HR 1.17, 95% CI 1.11–1.23, *P* < 0.001; Supplementary Table 7).

**Table 2:** Sequential cox proportional hazards models for the association between circulating CD38 and incident heart failure

Cohort	Model	Adjustments	HR per 1-SD	95% CI	*P* value
Overall	M1	Age, sex, BMI, hypertension, diabetes	1.248	1.184–1.316	< 0.001
Overall	M2	M1 + SBP, smoking, alcohol, hyperlipidemia, medications	1.212	1.149–1.278	< 0.001
Overall	M3	M2 + eGFR	0.989	0.923–1.059	0.748
Overall	M4	M3 + NT-proBNP, ANGPT2, IL-6	0.975	0.911–1.043	0.457
Women	M1	Age, BMI, hypertension, diabetes	1.392	1.278–1.516	< 0.001
Women	M2	M1 + SBP, smoking, alcohol, hyperlipidemia, medications	1.351	1.240–1.472	< 0.001
Women	M3	M2 + eGFR	1.166	1.042–1.305	0.007
Women	M4	M3 + NT-proBNP, ANGPT2, IL-6	1.096	0.979–1.227	0.112
Men	M1	Age, BMI, hypertension, diabetes	1.169	1.092–1.251	< 0.001
Men	M2	M1 + SBP, smoking, alcohol, hyperlipidemia, medications	1.138	1.063–1.217	< 0.001
Men	M3	M2 + eGFR	0.9	0.826–0.981	0.016
Men	M4	M3 + NT-proBNP, ANGPT2, IL-6	0.904	0.830–0.985	0.021

Hazard ratios are per 1-SD higher circulating CD38. Medication classes in M2 included statins, ACE inhibitors/ARBs, beta-blockers, diuretics, calcium channel blockers, and metformin. eGFR = estimated glomerular filtration rate calculated using the CKD-EPI 2021 creatinine-cystatin C equation. M3 eGFR adjustment leads to full statistical attenuation of the CD38–HF association overall and in men, and partial attenuation in women, reflecting kidney-dependent effect modification

### The CD38 sex paradox: higher levels in males but stronger risk in females

Analysis of sex-specific associations revealed an interesting paradox: despite males having substantially higher plasma CD38 than females (mean z-score 0.60 ± 0.85 vs. − 0.51 ± 0.83, *P* < 0.001), the CD38-heart failure association was paradoxically stronger in females (M1 HR 1.392, 95% CI 1.278–1.516) than males (M1 HR 1.169, 95% CI 1.092–1.251; interaction *P* = 0.004; Table 2). This counterintuitive finding, where the sex with lower biomarker levels experiences greater risk per unit increase, suggested the presence of sex-specific effect modifiers or distinct underlying mechanisms. TGF-β1, by contrast, showed no significant sex interaction (*P* = 0.098), with more similar effect sizes in females (HR 1.20, 95% CI 1.11–1.29) and males (HR 1.10, 95% CI 1.04–1.17) (Table 2). This sex-differential association persisted when CD38 was standardized within sex (females HR 1.31; males HR 1.14; interaction *P* = 0.005), suggesting that the differential association was not a scaling artifact. CD38 increased with age more steeply in females (β = 0.025 per year) than males (β = 0.007 per year; age × sex interaction *P* < 0.001), though age adjustment did not eliminate the sex-differential heart failure association (Fig. 2B). The CD38 × TGF-β1 interaction was non-significant in both minimally adjusted (*P* = 0.14) and fully adjusted models (*P* = 0.69), suggesting these biomarkers operate through largely independent pathways.

### Hormonal and reproductive modulation of CD38-associated Risk

In an exploratory analysis of women aged 45–60 years (*n* = 11,892; 184 events), the CD38–HF association appeared stronger in premenopausal than postmenopausal women (HR 2.44, 95% CI 1.62–3.69 vs. HR 1.33, 95% CI 1.12–1.58; interaction *P* = 0.0076) (Table 3; Fig. 2C). This difference persisted after adjustment for age, BMI, hypertension, and diabetes. A stronger association was observed in premenopausal women despite their lower absolute CD38 levels (mean NPX − 0.12 vs. + 0.08; *P* < 0.001) and fewer total events (20/3,033 vs. 164/8,859) (Fig. 2C–D, Supplementary Table 2 A). However, these observations remain exploratory given the small number of events (*n* = 20). To explore whether circulating estradiol contributed to the CD38–HF association, we examined correlations and performed an attenuation analysis in women with estradiol measurements (*n* = 5,232). CD38 showed a weak but statistically significant inverse correlation with estradiol (*r* = − 0.051, *P* = 0.0002), driven primarily by postmenopausal women (*r* = − 0.093, *P* < 0.001) (Supplementary Table 2B). Formal mediation analysis showed no evidence that estradiol mediated the CD38–HF relationship: the average causal mediation effect was near zero (*P* = 0.55), and the proportion mediated was less than 1% (*P* = 0.57) (Supplementary Table 2C). CD38 showed weak but statistically significant correlations with SHBG (women: *r* = − 0.15; men: *r* = − 0.12), free androgen index (women: *r* = 0.08; men: *r* = 0.08), and IGF-1 (women: *r* = − 0.09; men: *r* = 0.04) (all *P* < 0.001) (Supplementary Table 2B). These effect sizes are small (all r² < 0.03), and their significance reflects the large sample size rather than strong biological associations.

**Table 3:** Exploratory analysis: CD38–HF association by menopausal status (Women Aged 45–60)

Group	*N*	Events	Event Rate	HR (95% CI)	*P*-value
Premenopausal	3,033	20	0.66%	2.44 (1.62–3.69)	< 0.001
Postmenopausal	8,859	164	1.85%	1.33 (1.12–1.58)	0.001
**Interaction**	-	-	-	-	**0.0076**

Hazard ratios per 1-SD increment in CD38, adjusted for age, BMI, hypertension, and diabetes. Interaction P-value from the Cox model including CD38 × menopausal status term. Event rates show that premenopausal women have fewer total events despite a higher relative risk

Bold value indicates the interaction term between CD38 and menopausal status in the Cox model

###  Kidney function as an important context for the CD38 sex difference

**Fig. 3:**
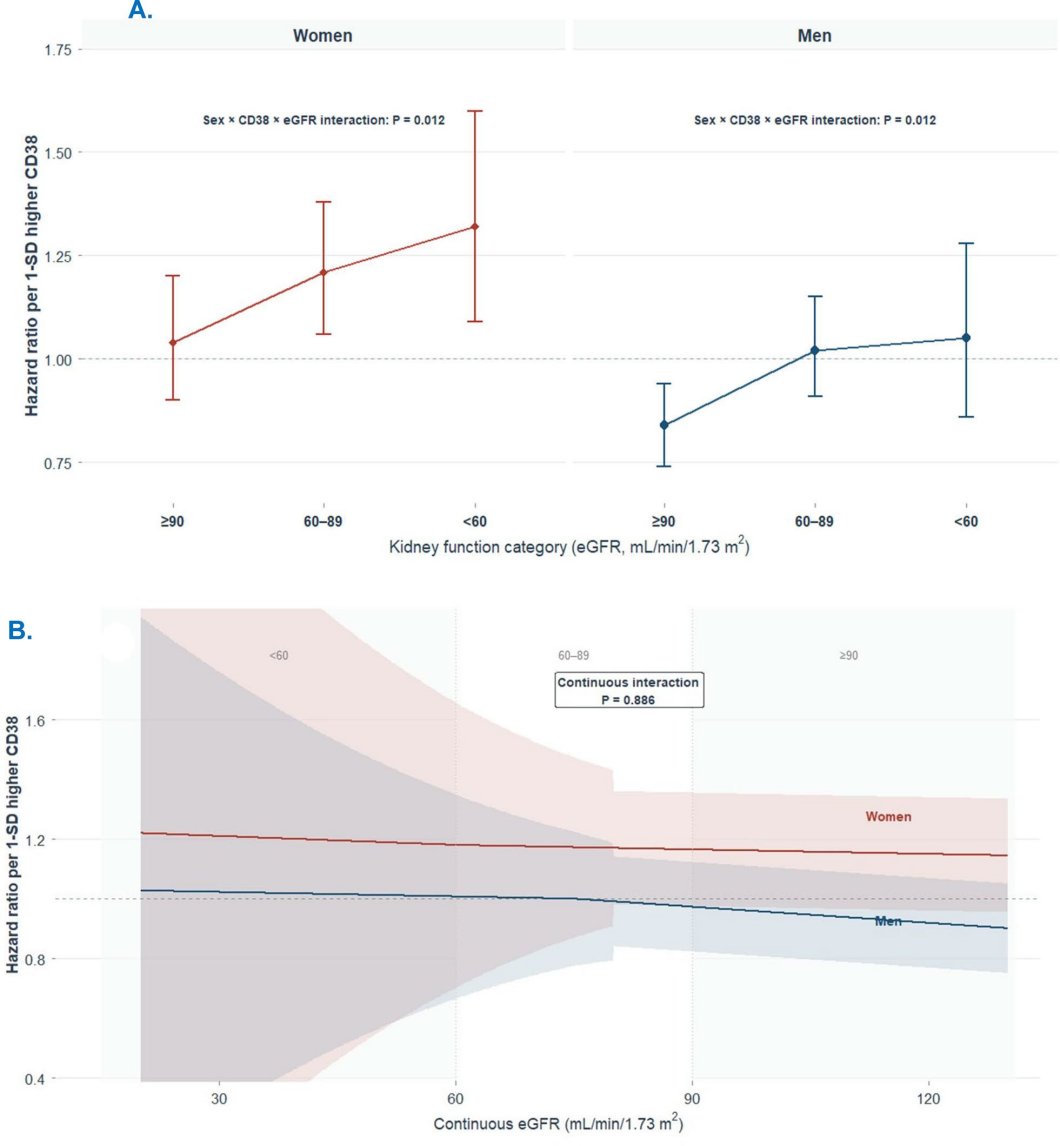
Kidney-function context and the CD38–heart failure association by sex. (**A**) Association of circulating CD38 with incident heart failure according to clinically defined kidney function strata in women and men. Sequential Cox models and eGFR-stratified estimates are shown to illustrate attenuation after adjustment for kidney function and downstream biomarkers, together with sex-specific HRs across eGFR categories. (**B**) Continuous modeling of the interaction between CD38, sex, and kidney function using eGFR as a continuous variable. In panel A, HRs and 95% CIs are shown per 1-SD higher CD38. eGFR categories were defined as ≥ 90, 60–89, and < 60 mL/min/1.73 m². In panel B, shaded bands represent 95% CIs around the modeled HRs. Models were adjusted for age, body mass index, hypertension, and diabetes unless otherwise indicated in the sequential model framework

Kidney function influenced the association between CD38 and incident HF. CD38 was inversely correlated with eGFR (*r* = − 0.48), whereas TGF-β1 showed a weaker correlation (*r* = − 0.22). In the sequential Cox framework, adjustment for traditional risk factors, medications, and lifestyle factors (M1→M2) produced only modest attenuation of the CD38–HF association (overall HR 1.248→1.212). In contrast, further adjustment for eGFR (M2→M3) abolished the overall association (HR 1.212→0.989, *P* = 0.75), whereas TGF-β1 retained a residual association after eGFR adjustment (HR 1.15→1.10) (Table 2). In sex-stratified models, eGFR adjustment attenuated but did not eliminate the association in women (HR 1.351→1.166, 95% CI 1.042–1.305, *P* = 0.007), whereas in men the association reversed direction after eGFR adjustment (HR 1.138→0.900, 95% CI 0.826–0.981, *P* = 0.016) and remained inverse after full biomarker adjustment (M4: HR 0.904, 95% CI 0.830–0.985, *P* = 0.021) (Fig. 3A). In three-way interaction analyses using categorical eGFR strata, CD38 showed significant effect modification by sex and kidney function (*P* = 0.012). Among women, there was no clear association at preserved kidney function (eGFR ≥ 90: HR 1.04, 95% CI 0.90–1.20), but progressively stronger associations emerged with declining eGFR (60–89: HR 1.21; < 60: HR 1.32). Among men, an inverse association was observed only in those with preserved kidney function (eGFR ≥ 90: HR 0.84, 95% CI 0.74–0.94, *P* = 0.002), whereas associations at lower eGFR levels were null (Fig. 3A) (Table 3).

When eGFR was modeled as a continuous variable, the three-way interaction between CD38, sex, and eGFR was not significant (*P* = 0.886; Fig. 3B), suggesting that the observed kidney-function heterogeneity may not follow a simple linear gradient. Sensitivity analysis using an alternative binary eGFR cutoff (≥ 60 vs. < 60 mL/min/1.73 m²) showed different association patterns by sex: the CD38–HF association remained significant in women (HR 1.265, 95% CI 1.149–1.393, *P* < 0.001) but was null in men (HR 1.019, 95% CI 0.947–1.098, *P* = 0.61).

### Biomarker attenuation analyses suggest divergent statistical pathway structures and sex-specific patterns

**Fig. 4:**
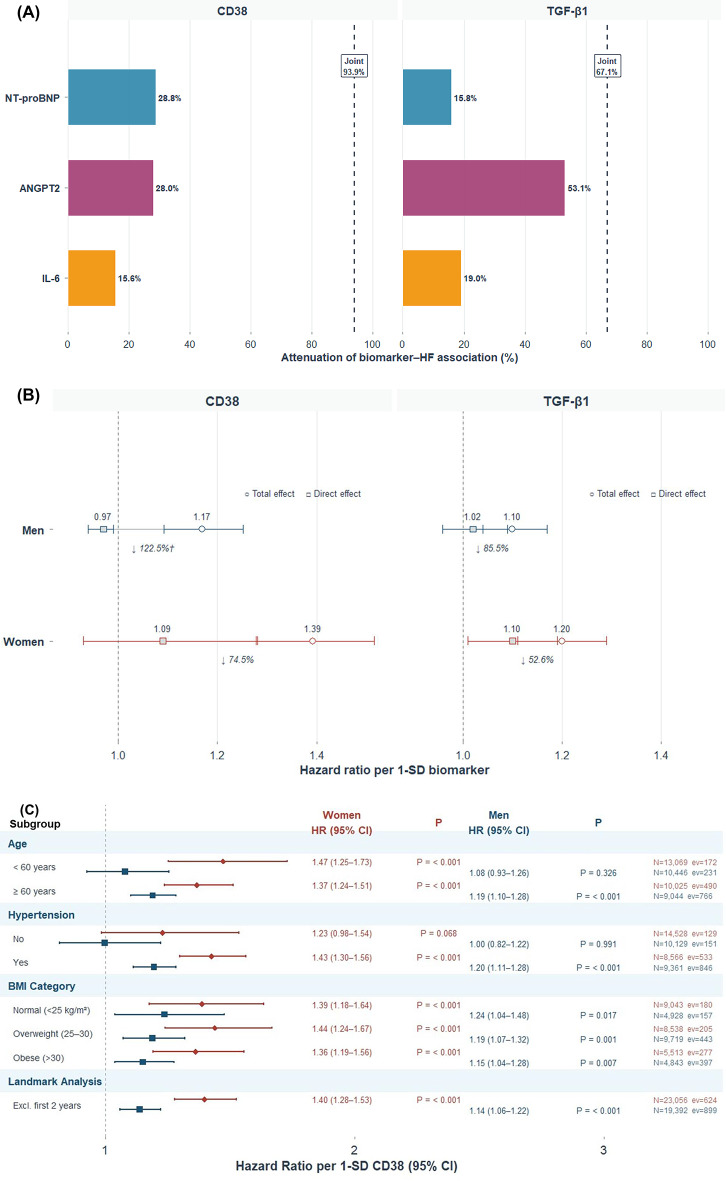

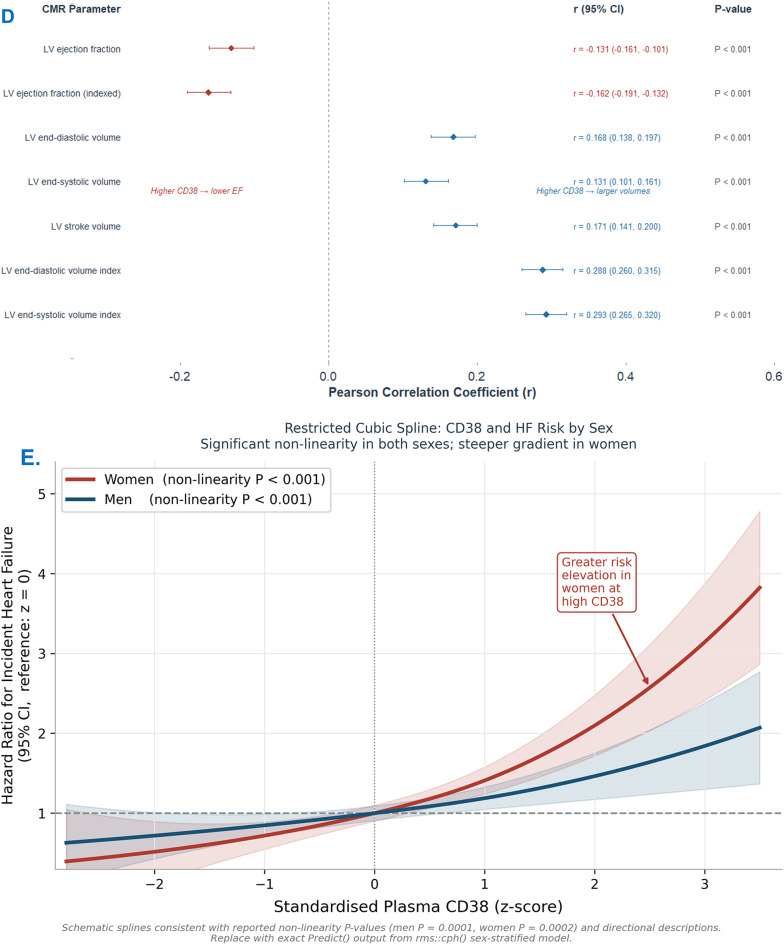
Pathway analyses and structural correlates of CD38.(**A**) Attenuation analysis showing the proportion of the CD38–heart failure and TGF-β1–heart failure associations explained by individual downstream biomarkers (NT-proBNP, ANGPT2, and IL-6), together with the corresponding joint attenuation. (**B**) Sex-stratified attenuation analyses for CD38 and TGF-β1. For each biomarker, total effects and direct effects after adjustment for NT-proBNP, ANGPT2, and IL-6 are shown separately in women and men; connecting lines indicate attenuation toward the null, and percentages denote the proportion of the association explained by the included downstream biomarkers. (**C**) Prespecified subgroup analyses of the CD38–heart failure association in women and men. (**D**) Associations between circulating CD38 and cardiac magnetic resonance (CMR)-derived left ventricular structure and function. Pearson correlation coefficients (r) and 95% CIs are shown; negative correlations indicate lower ejection fraction with higher CD38, whereas positive correlations indicate larger ventricular volumes with higher CD38. All correlations shown in panel D were statistically significant at *P* < 0.001. (**E**) Sex-stratified restricted cubic spline models for the association between circulating CD38 and incident heart failure. HRs are shown relative to the reference value at CD38 z-score = 0, with shaded areas indicating 95% CIs. Mediation methods, subgroup definitions, imaging variables, and spline modeling are described in the Methods and Supplementary Tables. Attenuation results reflect shared statistical variance among baseline biomarkers and are not intended to represent mechanistic pathway decomposition

To characterize shared statistical variance among baseline biomarkers, without implying causal directionality, joint biomarker attenuation analysis showed that adjustment for NT-proBNP, ANGPT2, and IL-6 reduced the CD38-HF association. Joint biomarker attenuation analysis showed that adjustment for NT-proBNP, ANGPT2, and IL-6 reduced the CD38-HF association (93.9% joint attenuation) more than the TGF-β1 association (67.1%), consistent with greater shared statistical variance with downstream biomarker burden. A supplementary path analysis estimated the total indirect pathway association through these three biomarkers at approximately 65% of the total association, a more conservative estimate that accounts for their correlated structure (Fig. 4A). Biomarker-specific attenuation patterns differed between CD38 and TGF-β1: CD38 showed relatively balanced contributions from NT-proBNP (28.8%), ANGPT2 (28.0%), and IL-6 (15.6%), whereas TGF-β1 attenuation was predominantly driven by ANGPT2 (53.1%), with smaller contributions from IL-6 (19.0%) and NT-proBNP (15.8%). Sex-stratified attenuation analysis revealed contrasting patterns for CD38 and TGF-β1 (Fig. 4B).

For CD38, males showed joint attenuation exceeding 100% (122.5%), with the direct effect reduced to near-unity after biomarker adjustment (HR 0.97, 95% CI 0.94–0.99), a pattern consistent with statistical suppression. In females, CD38 attenuation was 74.5%, leaving a residual direct association that did not reach statistical significance (HR 1.09, *P* = 0.23). For TGF-β1, the pattern was reversed: males showed near-complete attenuation (85.5%) with a null direct effect (HR 1.02, *P* = 0.63), whereas females retained a significant direct association after mediator adjustment (HR 1.10, *P* = 0.023) with 52.6% attenuation. These results reflect shared statistical variance among baseline biomarkers and should not be interpreted as mechanistic partitioning of causal effects.

### Subgroup robustness and biomarker context

The CD38–HF association was generally consistent across subgroups. In males, the association was significant among older participants (age ≥ 60: HR 1.19, *P* < 0.001), those with hypertension (HR 1.19, *P* < 0.001), and across all BMI categories (normal: HR 1.24, *P* = 0.02; overweight: HR 1.19, *P* < 0.001; obese: HR 1.13, *P* = 0.02). No association was observed in males without hypertension (HR 1.00, 95% CI 0.82–1.22, *P* = 0.996) or younger males (age < 60: HR 1.09, 95% CI 0.94–1.26, *P* = 0.27). In females, the association was robust across all subgroups examined: younger and older age, with and without hypertension, and across all BMI categories (Fig. 4C, Supplementary Table 3). After adjustment for multiple testing using the Benjamini-Hochberg procedure, no significant effect modification was detected by NT-proBNP, IL-6, ANGPT2, or age group (all FDR ≥ 0.10). Landmark analyses excluding early follow-up confirmed the robustness of findings. After excluding the first 2 years, the CD38–HF association remained significant in both males (HR 1.14, 95% CI 1.06–1.22, *P* < 0.001; 900 events) and females (HR 1.40, 95% CI 1.29–1.53, *P* < 0.001; 624 events). Results were similar after excluding the first 5 years (Supplementary Table 4).

### Cardiac structural correlates associates CD38 to heart failure phenotypes

In a subsample with cardiac magnetic resonance imaging (*n* = 4,175), higher CD38 was associated with lower LVEF (*r* = − 0.131) and higher LV volumes, with stronger correlations for indexed measures (LVEDV index *r* = 0.288; LVESV index *r* = 0.293) (Fig. 4D; Table 4).

**Table 4:** Correlation of CD38 with cardiac magnetic resonance imaging parameters (*n* = 4,175)

CMR Parameter	*r*	95% CI	*P*-value
LV Function			
LV ejection fraction	−0.131	−0.161, −0.101	1.5 × 10⁻¹⁷
LV ejection fraction (indexed)	−0.162	−0.191, −0.132	7.5 × 10⁻²⁶
LV Volumes			
LV end-diastolic volume	0.168	0.138, 0.197	9.1 × 10⁻²⁸
LV end-systolic volume	0.131	0.101, 0.161	1.8 × 10⁻¹⁷
LV stroke volume	0.171	0.141, 0.200	8.6 × 10⁻²⁹
LV Volumes (Indexed)			
LV end-diastolic volume index	0.288	0.260, 0.315	2.5 × 10⁻⁸⁰
LV end-systolic volume index	0.293	0.265, 0.320	3.6 × 10⁻⁸³

Pearson correlation coefficients with 95% confidence intervals. Indexed measures are corrected for body surface area. CMR, cardiac magnetic resonance; LV, left ventricular; LVEF, left ventricular ejection fraction; LVEDV, left ventricular end-diastolic volume; LVESV, left ventricular end-systolic volume. All correlations are significant at *P* < 0.001

### Predictive discrimination and clinical utility

We assessed whether CD38 provided incremental prognostic value beyond established clinical and biomarker predictors. Model diagnostics showed the proportional hazards assumption was satisfied for CD38 in both males (Schoenfeld test *P* = 0.57) and females (*P* = 0.27). Testing for non-linearity revealed significant non-linear effects in both sexes (males: *P* = 0.0001; females: *P* = 0.0002), with the spline curves indicating a steeper increase in heart failure risk at higher CD38 levels, particularly among females (Fig. 4E). Addition of CD38 to a base clinical risk model (including age, sex, BMI, eGFR, hypertension, diabetes, NT-proBNP, ANGPT2) showed minimal improvement in predictive discrimination. Using IPCW-adjusted metrics: 5-year AUC increased from 85.0% to 85.0% (ΔAUC = 0.1%, *P* = 0.04 but clinically negligible). The Integrated Discrimination Improvement (IDI) was 0.000 (95% CI: 0.000–0.001.000.001, *P* = 0.64), and the continuous Net Reclassification Improvement (NRI) was 0.060 (95% CI: −0.134 to 0.111, *P* = 0.22). Neither metric achieved statistical or clinical significance, confirming that CD38 provides no incremental prognostic value beyond established biomarkers (Supplementary Table 4).

### Isoproterenol induces sex-differential metabolic and renal stress responses in mice

**Fig. 5:**
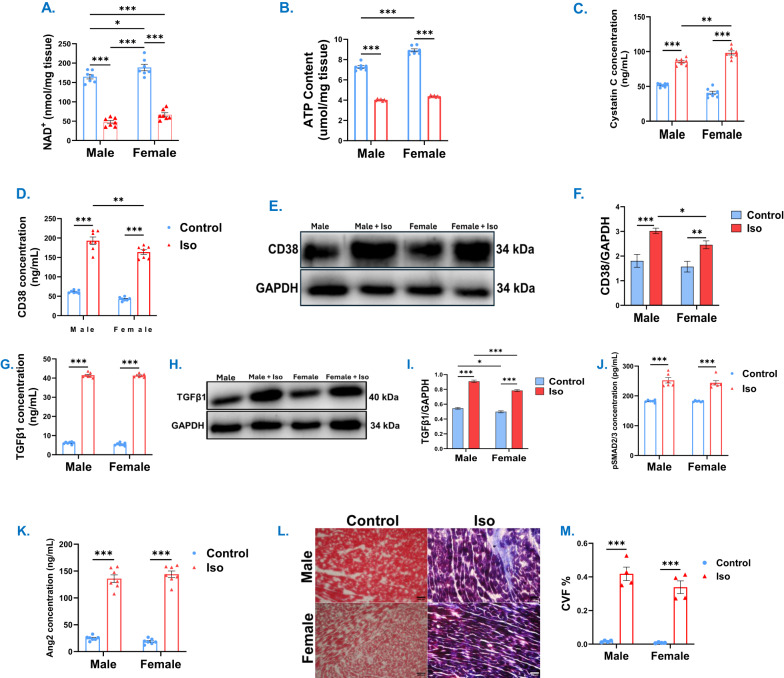
Baseline sex differences and chronic β-adrenergic stress responses in mice. Male and female FVB mice received vehicle or ISO (2.5 mg/kg/day, subcutaneous) for 21 days. Panels show cardiac NAD⁺ (**A**), cardiac ATP (**B**), plasma cystatin C (**C**), plasma CD38 (**D**), cardiac CD38 protein (**E–F**), plasma TGF-β1 (**G**), cardiac TGF-β1 protein (**H–I**), cardiac pSMAD2/3 (**J**), plasma ANGPT2 (**K**), and myocardial collagen volume fraction with representative Masson’s trichrome images (**L**) and quantification (**M**). ISO significantly increased collagen volume fraction in both sexes (treatment *P* < 0.001; η²=0.916); no significant sex effect (*P* = 0.136) or sex × treatment interaction (*P* = 0.221) was observed. Data are mean ± SEM. Two-way ANOVA with sex and treatment as factors; n per group as indicated (*n* = 4 per group for histological analyses). Representative immunoblots are shown where applicable

At baseline, female mice exhibited higher cardiac NAD⁺ (*P* = 0.002; Fig. 5A), higher cardiac ATP (*P* < 0.001; Fig. 5B), and lower plasma cystatin C than males (40.6 vs. 51.8 ng/mL, *P* = 0.008; Fig. 5C), indicating superior baseline renal function. Circulating CD38 was higher in males (*P* < 0.001; Fig. 5D), consistent with the human cohort, though cardiac CD38 protein did not differ at baseline (*P* = 0.495; Fig. 5E **– **F). Plasma TGF-β1 was similar between sexes (*P* = 0.246; Fig. 5G), while cardiac TGF-β1 was modestly higher in males (*P* = 0.019; Fig. 5H **–** I). pSMAD2/3 and ANGPT2 showed no baseline sex differences (Fig. 5J **– **K).

Echocardiographic assessment indicated that the ISO protocol induced cardiac structural and functional changes consistent with a cardiac stress phenotype in both sexes (Fig. S1 A – C). ISO increased LV mass in both sexes (overall ISO effect *P* < 0.001) and reduced ejection fraction (*P* = 0.002). A significant sex effect reflected higher baseline systolic function in females (EF 75.4% vs. 68.0%, *P* = 0.041); post-ISO, EF declined significantly in females (*P* = 0.018) but not in males (*P* = 0.312), though the sex × treatment interaction was non-significant (*P* = 0.282).

ISO induced severe NAD⁺ depletion in both sexes (*P* < 0.001; Fig. 5A), eliminating the female baseline advantage (post-ISO: 47.7 vs. 66.4 arbitrary units, *P* = 0.153; interaction *P* = 0.679). Despite comparable NAD⁺ depletion, downstream energetic consequences differed: females showed a significantly greater absolute ATP decline (51% vs. 45%; interaction *P* < 0.001, η² = 0.024; Fig. 5B), starting from a higher set-point but reaching similarly depleted post-ISO levels. Renal stress was markedly exaggerated in females: cystatin C increased 142% in females versus 65% in males (interaction *P* < 0.001, η² = 0.061; Fig. 5C), with females ending at significantly higher absolute levels despite lower baseline (98.1 vs. 85.5 ng/mL, *P* = 0.003).

Cardiac CD38 was upregulated by ISO in both sexes (*P* < 0.001; Fig. 5E **– **F), with males achieving higher absolute post-ISO levels (3.022 vs. 2.459 arbitrary units, *P* = 0.032) but comparable induction magnitude (interaction *P* = 0.186). Plasma TGF-β1 increased comparably in both sexes (interaction *P* = 0.436; Fig. 5G), while cardiac TGF-β1 showed a larger ISO-induced increase in males (interaction *P* = 0.001, η² = 0.014; Fig. 5H **– **I). Despite this, pSMAD2/3 activation was sex-equivalent (interaction *P* = 0.505; Fig. 5J). ANGPT2 was equally upregulated in both sexes (interaction *P* = 0.154; Fig. 5K). Masson’s trichrome staining of Phase 1 hearts revealed that ISO increased collagen volume fraction in both sexes (treatment *P* < 0.001; η² = 0.916), with no significant sex effect (*P* = 0.136) or sex × treatment interaction (*P* = 0.221; Fig. 5L **– **M), indicating that ISO-induced fibrotic remodeling did not differ between sexes at baseline.

### CD38 and TGF-β1 inhibition reveal distinct pathogenic axes

**Fig. 6:**
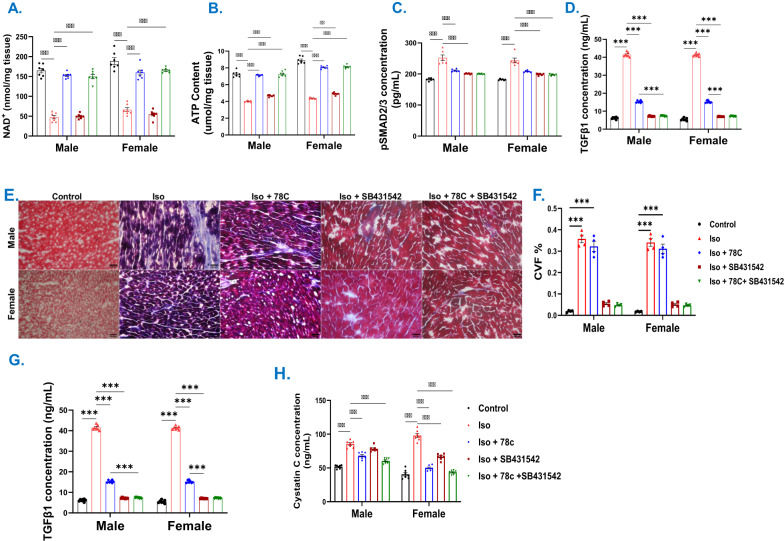
Pharmacologic inhibition of CD38 and TGF-β signaling during β-adrenergic stress.Male and female mice received ISO (2.5 mg/kg/day, subcutaneous) alone or with 78c (10 mg/kg/day, intraperitoneal), SB431542 (10 mg/kg/day, intraperitoneal), or both for 21 days. Panels show cardiac NAD⁺ (**A**), cardiac ATP (**B**), circulating pSMAD2/3 (**C**), plasma TGF-β1 (**D**), myocardial collagen volume fraction with representative Masson’s trichrome images (**E**) and quantification **(F**), plasma ANGPT2 (**G**), and plasma cystatin C (**H**). 78c did not significantly reduce collagen volume fraction versus ISO in either sex (male *P* = 0.673, female *P* = 0.842). SB431542 normalised collagen volume fraction to vehicle control levels in both sexes (*P* < 0.001 vs. ISO; *P* > 0.6 vs. control); the combination showed no additive benefit beyond SB431542 alone (*P* > 0.999). No significant sex effect or sex × treatment interaction was observed for fibrosis outcomes (all *P* > 0.4). Data are mean ± SEM; n per group as indicated (*n* = 4 per group for histological analyses). Two-way ANOVA

78c significantly restored ISO-induced NAD⁺ depletion in both sexes (*P* < 0.001 vs. ISO; Fig. 6A), with equivalent efficacy between sexes (interaction *P* = 0.289). ATP was similarly normalised (*P* < 0.001 vs. ISO; Fig. 6B). SB431542 had no significant effect on NAD⁺ or ATP (*P* > 0.99 vs. ISO), and adding SB431542 to 78c had no additional metabolic benefit (*P* > 0.05 vs. 78c alone). Conversely, SB431542 completely normalised pSMAD2/3 to control levels in both sexes (*P* < 0.001 vs. ISO; interaction *P* = 0.869; Fig. 6C) and normalised plasma TGF-β1 (*P* < 0.001 vs. ISO; Fig. 6D). 78c had no significant effect on either (*P* > 0.05 vs. ISO). This reciprocal specificity, 78c rescuing metabolism but not fibrotic signalling, SB431542 blocking fibrotic signalling but not metabolism, suggests independent upstream pathways. Masson’s trichrome staining provided structural-level evidence consistent with this independence (Fig. 6E **–** F). ISO induced marked cardiac fibrosis in both sexes (overall ISO effect *P* < 0.001). 78c had no effect on collagen volume fraction (male: *P* = 0.673; female: *P* = 0.842 vs. ISO). SB431542 normalised CVF to control levels in both sexes (male 5.4%, *P* = 0.601 vs. control; female 5.1%, *P* = 0.720 vs. control; both *P* < 0.001 vs. ISO), with the combination producing CVF levels identical to those of SB431542 alone (*P* > 0.999). No sex difference in fibrosis was observed under any condition (sex effect *P* = 0.419; interaction *P* = 0.970). Renal CD38 mRNA was significantly upregulated by ISO (*P* < 0.001; Fig. S1 E). The sex × treatment interaction approached significance (*P* = 0.098): females showed a significant increase (*P* = 0.003) while males showed a non-significant trend (*P* = 0.191). These directional findings are consistent with the cystatin C and cardiac CD38 patterns, but should be interpreted cautiously given the small sample sizes (*n* = 4 per group).

### Regulation of downstream mediators and combined inhibition suggests sex-differential downstream responses in renal stress-related markers

ISO increased plasma ANGPT2 in both sexes (*P* < 0.001; Fig. 6G), attenuated by both inhibitors, with a significant sex × treatment interaction (*P* < 0.001, η² = 0.026). SB431542 was the dominant ANGPT2 suppressor. Renal protection showed marked sex differences (interaction *P* < 0.001, η² = 0.093; Fig. 6H). 78c reduced cystatin C by 49% in females (*P* < 0.001 vs. ISO) and 20% in males (non-significant). SB431542 reduced cystatin C by 32% in females (*P* < 0.001) and 8% in males (non-significant). Combined inhibition normalised cystatin C to control levels in females only (*P* > 0.99 vs. control); males receiving combination therapy remained significantly elevated (*P* < 0.05 vs. control). No additive interaction was observed for NAD⁺ or pSMAD2/3, consistent with independent upstream mechanisms converging downstream on integrated organ injury.

## Discussion

In a cohort of more than 42,000 adults followed for over a decade, circulating CD38 was associated with incident heart failure (HF) in minimally adjusted models, showed strong statistical overlap with biomarkers of cardiac stress, vascular dysfunction, and inflammation, and provided little incremental prognostic value beyond established downstream biomarkers. These findings suggest that CD38 may be an upstream biomarker reflecting cardiometabolic stress context rather than an independent predictor of HF. The revised analyses further indicate that this relationship differs by sex and varies across kidney-function contexts.

### Sex-divergent CD38 biology and heart failure risk

The most notable finding of the human analyses is the stronger association between CD38 and incident HF in women despite substantially lower circulating CD38 levels than in men. This dissociation between biomarker levels and biomarker-associated risk has been reported in cardiovascular epidemiology [27–31], but to our knowledge has not been previously described for CD38. The sex interaction was specific to CD38 and was not observed for TGF-β1, supporting pathway specificity rather than a generalized sex difference in circulating mediators. The stronger female association persisted after within-sex standardization, across clinical subgroups, and in landmark analyses excluding early events, providing some evidence against strong scaling effects or reverse causation, though this cannot be excluded.

Several observations support the view that this pattern is unlikely to be explained entirely by measured confounders. Adjustment for age [32, 33], metabolic comorbidities, medication use, and inflammatory markers did not remove the sex interaction. In an exploratory subgroup analysis, the association appeared stronger in premenopausal than postmenopausal women, although this finding was based on only 20 premenopausal HF events and requires replication. Estradiol showed only a weak inverse correlation with CD38, and the available hormone analyses did not support a major role for circulating estradiol in accounting for the observed association. Similarly, additional adjustment for waist circumference and body fat percentage produced negligible changes in the CD38 hazard ratios, suggesting that body composition beyond BMI does not explain the primary findings. Residual confounding by socioeconomic, behavioral, reproductive, or treatment-related factors nonetheless remains possible.

The biological basis of the stronger female association remains uncertain. At baseline, female mice exhibited higher cardiac NAD⁺ and ATP levels than males, suggesting a more favorable energetic state [34]. Under β-adrenergic stress, both sexes experienced marked NAD⁺ depletion, effectively removing this baseline female advantage. CD38 inhibition restored NAD⁺ and ATP in both sexes, with numerically greater downstream benefit in females. These observations are consistent with the possibility that female hearts operate at a higher energetic set-point that may allow greater absolute metabolic loss under stress, although this interpretation remains inferential. The present study cannot distinguish whether this vulnerability is driven predominantly by gonadal hormones, broader reproductive metabolic context, sex chromosomes, or developmental programming, and these possibilities are not mutually exclusive.

### Kidney function as an important context for CD38-HF associations

The second notable finding concerns the influence of kidney-function context on the CD38-HF association. CD38 was more strongly correlated with eGFR than TGF-β1, and adjustment for eGFR abolished the overall CD38-HF association while only modestly attenuating TGF-β1. In sex-stratified models, eGFR adjustment attenuated but did not eliminate the association in women, whereas in men, the association reversed direction after eGFR adjustment and remained inverse after further biomarker adjustment. These results indicate that kidney function is an important context for interpreting circulating CD38.

The interpretation of this kidney-function sensitivity is not straightforward. It may reflect biological involvement of the kidney in the CD38 stress response [35, 36], confounding by renal and metabolic health [37], altered clearance of circulating CD38 [38, 39], or a combination of these mechanisms. The categorical interaction analyses suggested clinically meaningful heterogeneity across eGFR strata. In contrast, the corresponding continuous interaction was not significant, indicating that the observed pattern may not follow a simple linear gradient. In addition, alternative eGFR parameterization preserved the female association but rendered the male association null, suggesting that the female pattern is more stable, whereas the male inverse pattern is more sensitive to how kidney function is modeled. For this reason, the inverse association observed in men should not be overinterpreted as evidence of biological protection.

The murine data provide supportive evidence of renal involvement but do not establish structural renal injury or causal mediation. ISO increased cystatin C and renal CD38 mRNA overall, and pathway-directed interventions attenuated these responses. However, because direct renal histology and tubular injury markers were not assessed, the present data are better interpreted as showing renal engagement of the CD38 stress response rather than definitive renal protection [19, 40]. Future studies in primary renal disease models and genetically informed human analyses may help clarify whether kidney function primarily confounds, modifies, or lies on part of the pathway linking CD38 to HF risk.

### CD38 and TGF-β1 mark complementary axes

The human biomarker analyses suggest that CD38 and TGF-β1 relate to HF via partially distinct downstream pathways. CD38 showed strong attenuation after adjustment for NT-proBNP, ANGPT2, and IL-6, consistent with substantial statistical overlap with biomarkers of cardiac stress, vascular dysfunction, and inflammation. TGF-β1 also showed attenuation, but the pattern differed in magnitude and sex stratification. These findings suggest that CD38 and TGF-β1 occupy overlapping but non-identical positions within the broader stress-response network captured by circulating biomarkers.

The murine intervention studies support this distinction. CD38 inhibition restored the metabolic deficit, improving cardiac NAD⁺ and ATP, but did not meaningfully reduce fibrosis [14]. In contrast, TGF-β receptor inhibition reduced pSMAD2/3 signaling and fibrosis, but did not rescue the energetic endpoints. Combined treatment improved downstream integrated markers such as cystatin C and ANGPT2 more than either monotherapy, whereas proximal readouts remained pathway-specific. Together, these proof-of-concept results are consistent with a model of complementary rather than identical pathogenic axes, with CD38 linked more closely to metabolic stress and TGF-β1 to fibrotic remodeling; they are best interpreted as mechanistic support for further investigation rather than direct evidence for clinical application. This observation is consistent with the emerging hypothesis that HF involves parallel, interacting pathogenic processes rather than a single linear cascade [41, 42].

### CD38 as a mechanistic rather than prognostic biomarker

Although CD38 was associated with incident HF in minimally adjusted analyses, it provided little incremental prognostic value beyond established risk markers and downstream biomarkers. This pattern is consistent with its position as an upstream pathway biomarker rather than a clinical risk-discrimination tool. A biomarker may still have translational importance even if it adds little to prediction, provided that it identifies a modifiable biological process [43, 44]. In that sense, the value of CD38 may lie more in what it reveals about upstream stress biology and potential pathway-directed investigation than in improving risk prediction over existing biomarker panels.

### Limitations

This study has several limitations. The human analyses are observational and cannot establish causality. CD38 was measured once in relative units, limiting temporal and clinical interpretation. The final analytic cohort was derived through complete-case analysis, so selection bias cannot be ruled out. The kidney-function interaction was stronger in categorical than continuous models, and spline-based interaction analyses should be considered exploratory. The menopausal analysis was exploratory, relied on an age-based proxy rather than confirmed menopausal status, and included only 20 HF events in premenopausal women. Estradiol was available in only a subset of women, limiting inference regarding hormonal mechanisms. HF subtypes could not be distinguished from ICD-10 codes, so subtype-specific interpretations remain hypothesis-generating. In mice, the experiments were not powered for interaction testing, used a preventive pharmacological design, and were performed in an isoproterenol model of β-adrenergic stress; extension to other HF models requires a dedicated study. In addition, the gonad-intact murine design does not allow direct separation of hormonal from chromosomal or developmental sex effects. Finally, the predominantly European UK Biobank population may limit generalizability. Despite these constraints, the concordance of the human and preclinical findings supports further investigation of CD38 as a sex-differential marker of HF vulnerability.

## Perspectives and significance

This study shows that the association between circulating CD38 and incident HF differs by sex and varies by kidney-function context. In women, CD38 remained positively associated with HF after kidney adjustment, whereas in men, the association became inverse or null depending on model specification, underscoring the importance of renal context in interpreting CD38-related risk. These findings support further mechanistic investigation of CD38-linked NAD⁺ biology in sex-aware cardiovascular research and suggest that the broader phenomenon of lower biomarker levels but stronger associated risk in women may warrant attention across other upstream cardiometabolic biomarkers. At present, however, these results are best viewed as a framework for further mechanistic and translational work rather than a basis for immediate clinical implementation.

## Electronic Supplementary Material

Below is the link to the electronic supplementary material.

Supplementary material 1.

Supplementary material 2.

Supplementary material 3.

## Data Availability

The data supporting the findings of this study are available within the article and the supplementary materials. Codes used for the UK Biobank data analysis are available upon reasonable request. Further inquiries may be directed to the corresponding author.

## Abbreviations

ACEi/ARB: Angiotensin-Converting Enzyme Inhibitor/Angiotensin Receptor Blocker
IL-6: Interleukin-6
ALT: Alanine Aminotransferase
ISO: Isoproterenol
ANP: Atrial Natriuretic Peptide
IQR: Interquartile Range
ANGPT2: Angiopoietin-2
LDL-C: Low-Density Lipoprotein Cholesterol
AST: Aspartate Aminotransferase
LV: Left Ventricular
ATP: Adenosine Triphosphate
LVEDV: Left Ventricular End-Diastolic Volume
BMI: Body Mass Index
LVEF: Left Ventricular Ejection Fraction
BNP: Brain Natriuretic Peptide
LVESV: Left Ventricular End-Systolic Volume
BP: Blood Pressure
LVFS: Left Ventricular Fractional Shortening
CCB: Calcium Channel Blocker
LVSV: Left Ventricular Stroke Volume
CD38: Cluster of Differentiation 38
NPX: Normalized Protein Expression
CMR: Cardiac Magnetic Resonance
NT-proBNP: N-Terminal pro-B-type Natriuretic Peptide
CRP: C-Reactive Protein
PBS: Phosphate-Buffered Saline
CV: Cardiovascular
PVDF: Polyvinylidene Fluoride
GAPDH: Glyceraldehyde-3-Phosphate Dehydrogenase
RCS: Restricted Cubic Splines
HDL-C: High-Density Lipoprotein Cholesterol
RT-PCR: Reverse Transcription Polymerase Chain Reaction
HF: Heart Failure: SD: Standard Deviation
HR: Hazard Ratio: SEM: Standard Error of the Mean
ICD-10: International Classification of Diseases, Tenth Revision
TGF-β1: Transforming Growth Factor Beta 1
HR: Hazard Ratio: TNNI3: Cardiac Troponin I
ICD10: International Classification of Diseases, Tenth Revision
