# Supplementary material for: Circulating CD38 shows stronger association with incident heart failure in women despite lower levels: sex differences and kidney-function context
Source: Biol Sex Differ. 2026 Jun 4;17:144. doi: 10.1186/s13293-026-00924-7 (PMC13449966; doi:10.1186/s13293-026-00924-7)

Fig. 1: Study flowchart

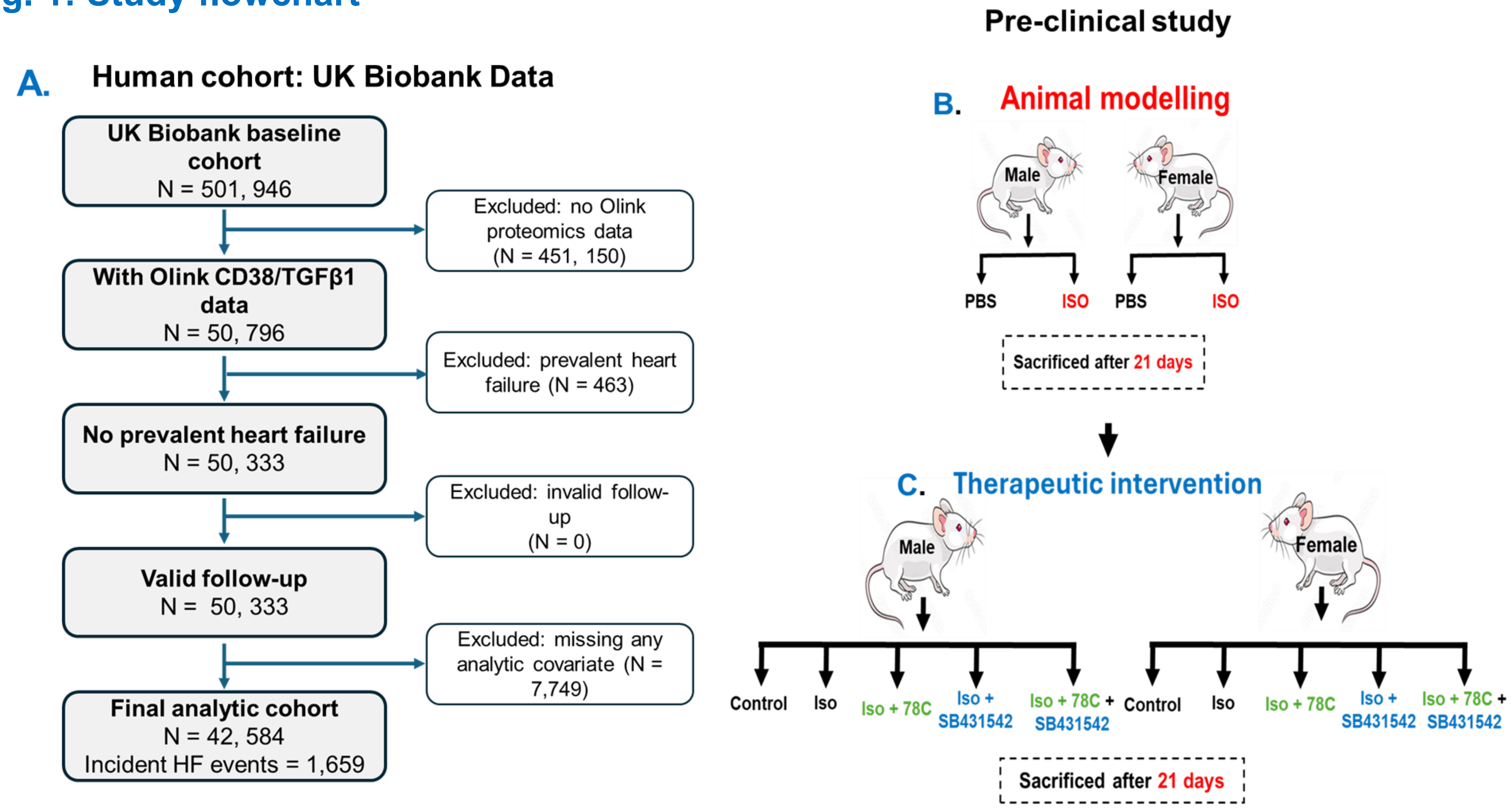

Fig. 2

A.

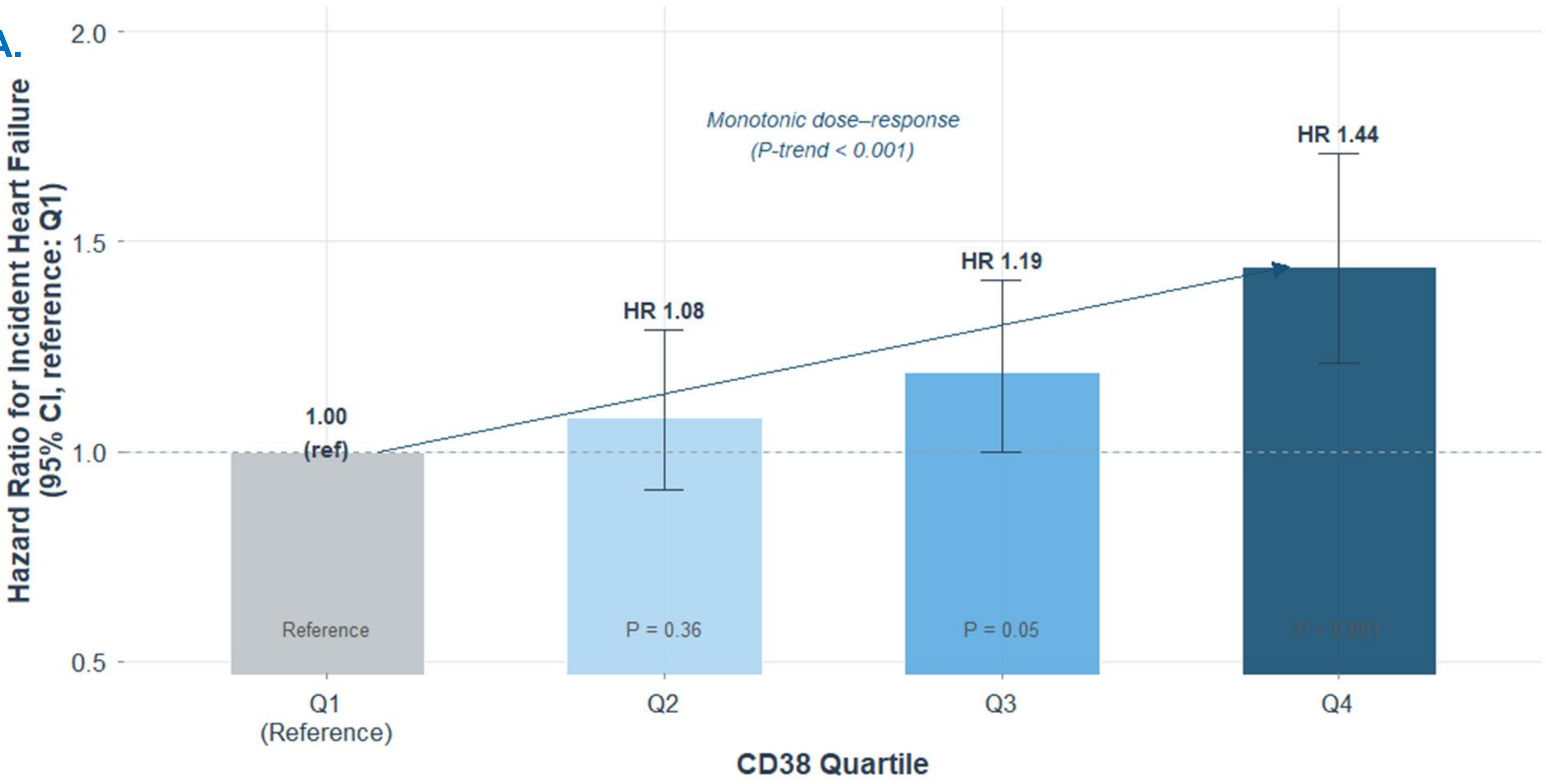
Hazard Ratio for Incident Heart Failure
(95% CI, reference: Q1)
2.0
1.5
1.0
0.5
Monotonic dose–response
(P-trend < 0.001)
1.00
(ref)
HR 1.08
HR 1.19
HR 1.44
Reference
P = 0.36
P = 0.05
P < 0.001
Q1
(Reference)
Q2
Q3
Q4
CD38 Quartile

Fig. 2

B.

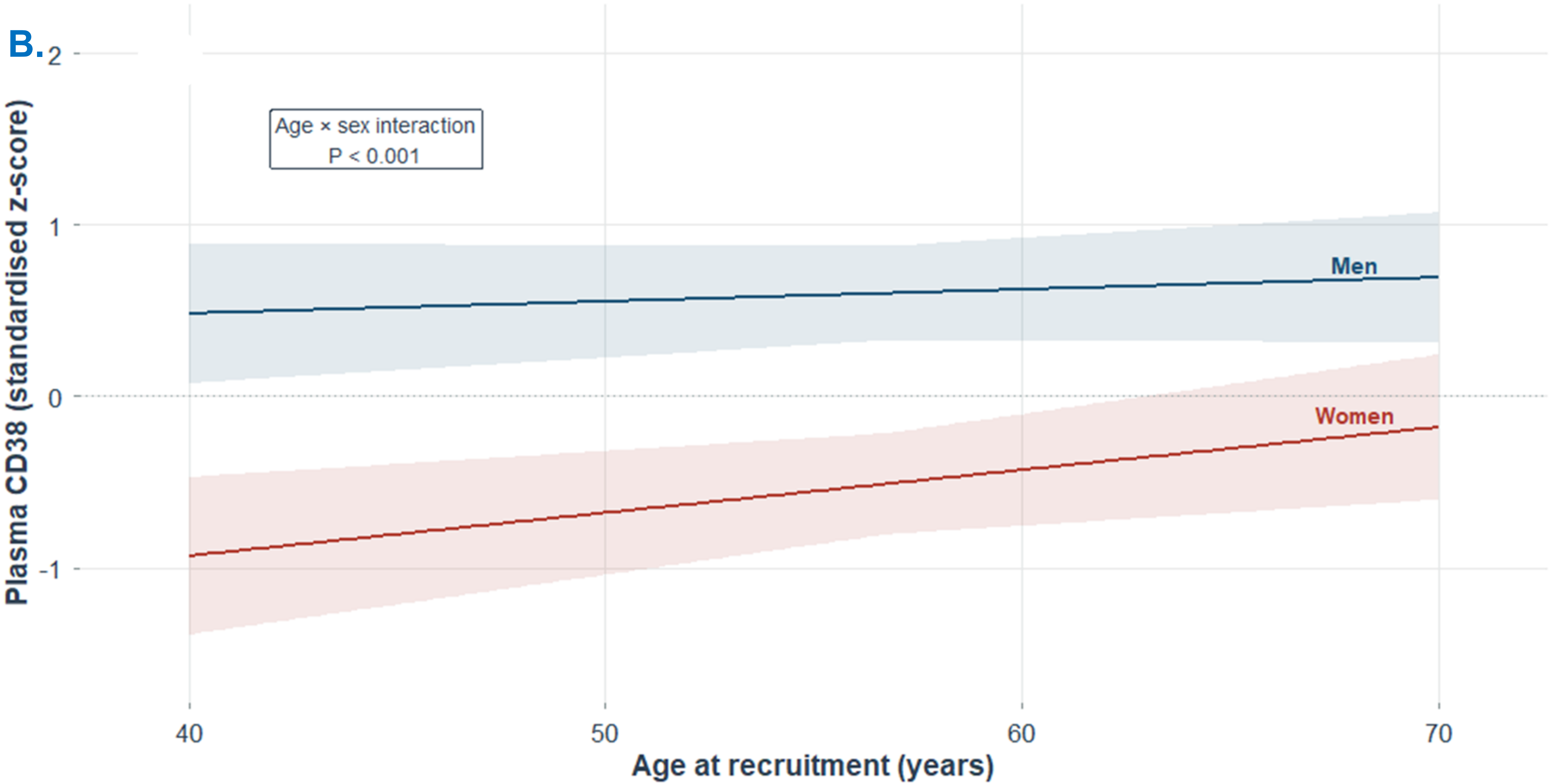
Age × sex interaction
P < 0.001
Men
Women
Plasma CD38 (standardised z-score)
2
1
0
-1
40
50
60
70
Age at recruitment (years)

Fig. 2

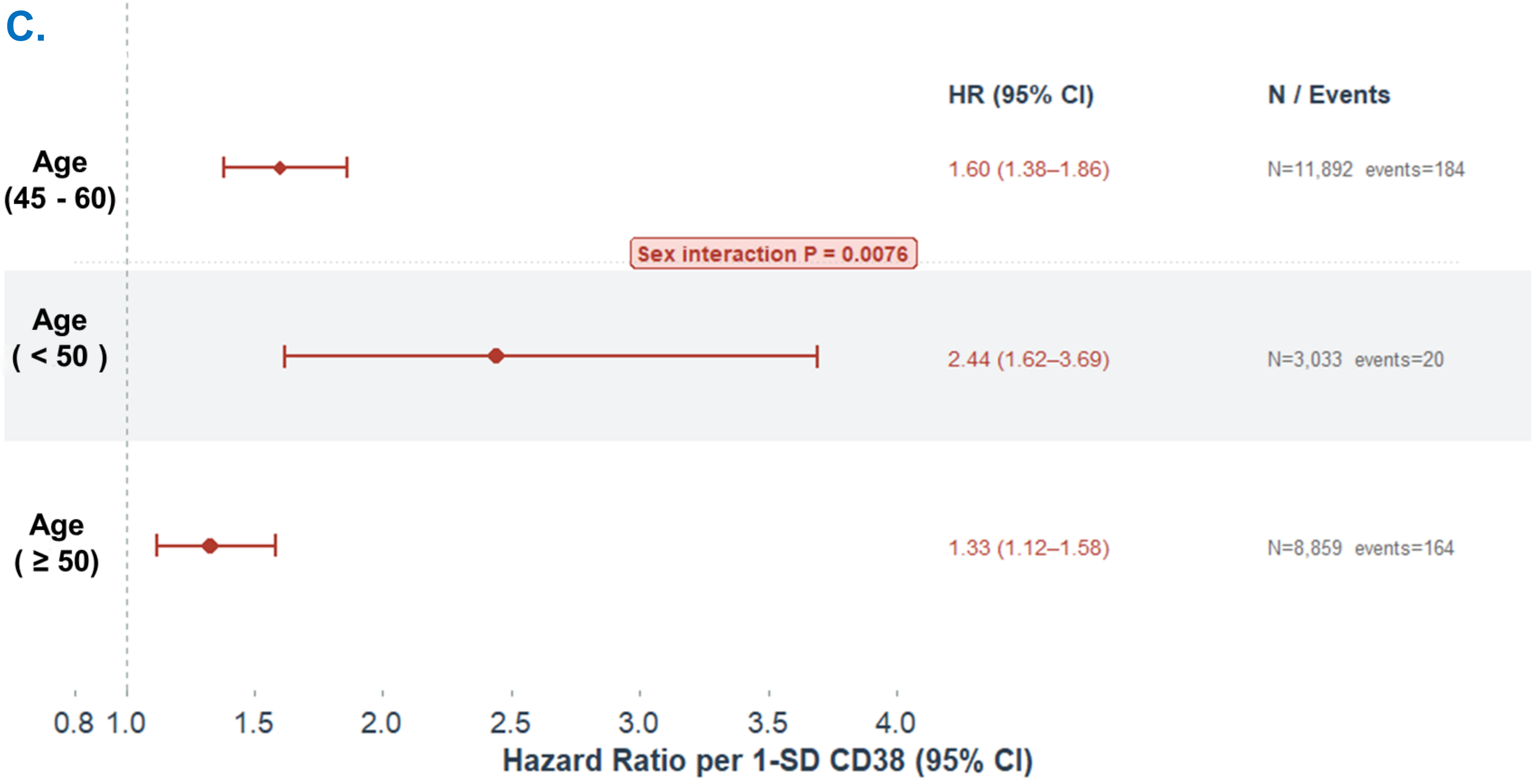
C.
HR (95% CI)
N / Events
Age
(45 - 60)
1.60 (1.38–1.86)
N=11,892 events=184
Sex interaction P = 0.0076
Age
(< 50)
2.44 (1.62–3.69)
N=3,033 events=20
Age
(≥ 50)
1.33 (1.12–1.58)
N=8,859 events=164
0.8 1.0 1.5 2.0 2.5 3.0 3.5 4.0
Hazard Ratio per 1-SD CD38 (95% CI)

Fig. 2

D.

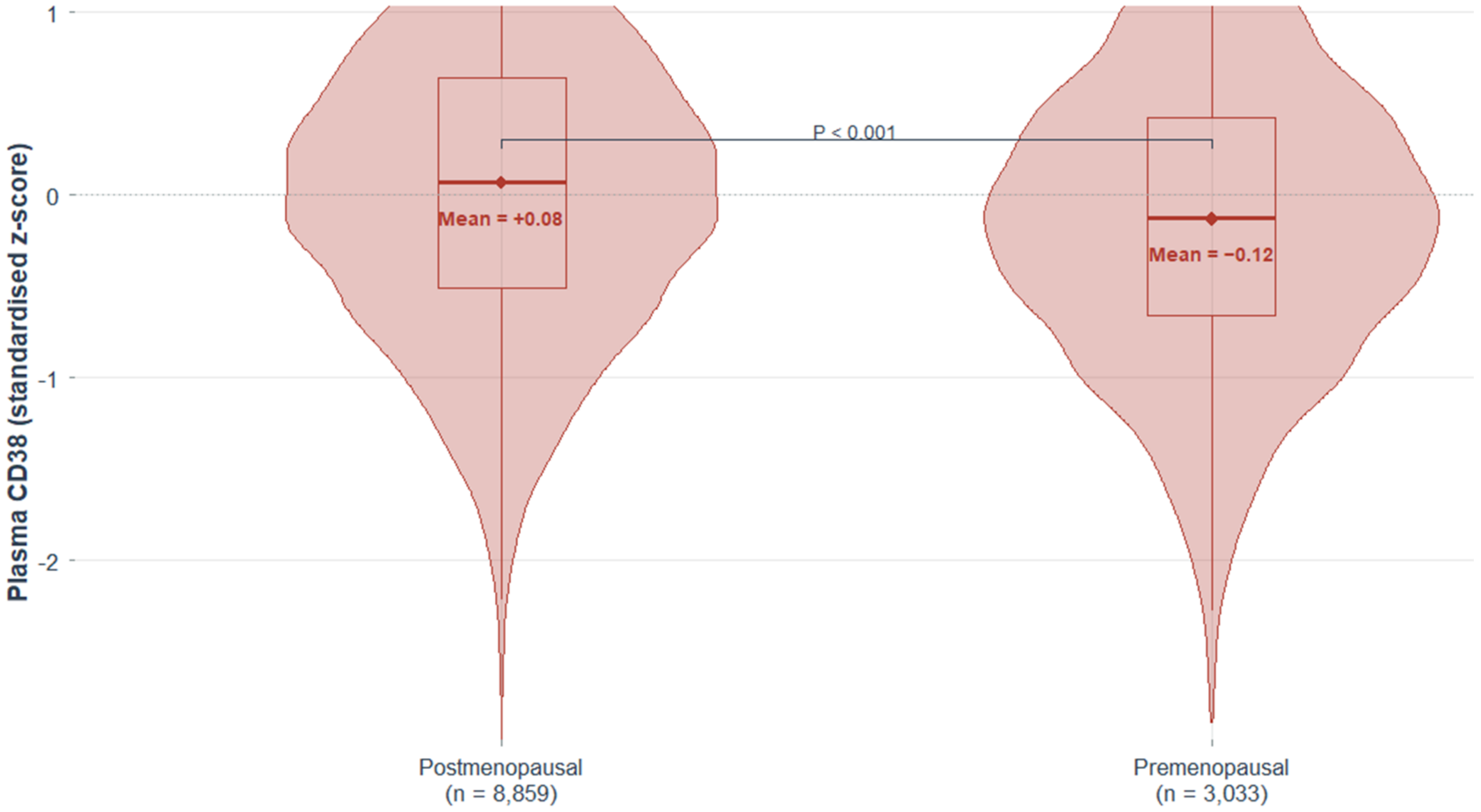

Plasma CD38 (standardised z-score)
1
0
-1
-2
P < 0.001
Mean = +0.08
Mean = −0.12
Postmenopausal
(n = 8,859)
Premenopausal
(n = 3,033)

Fig. 3 A.

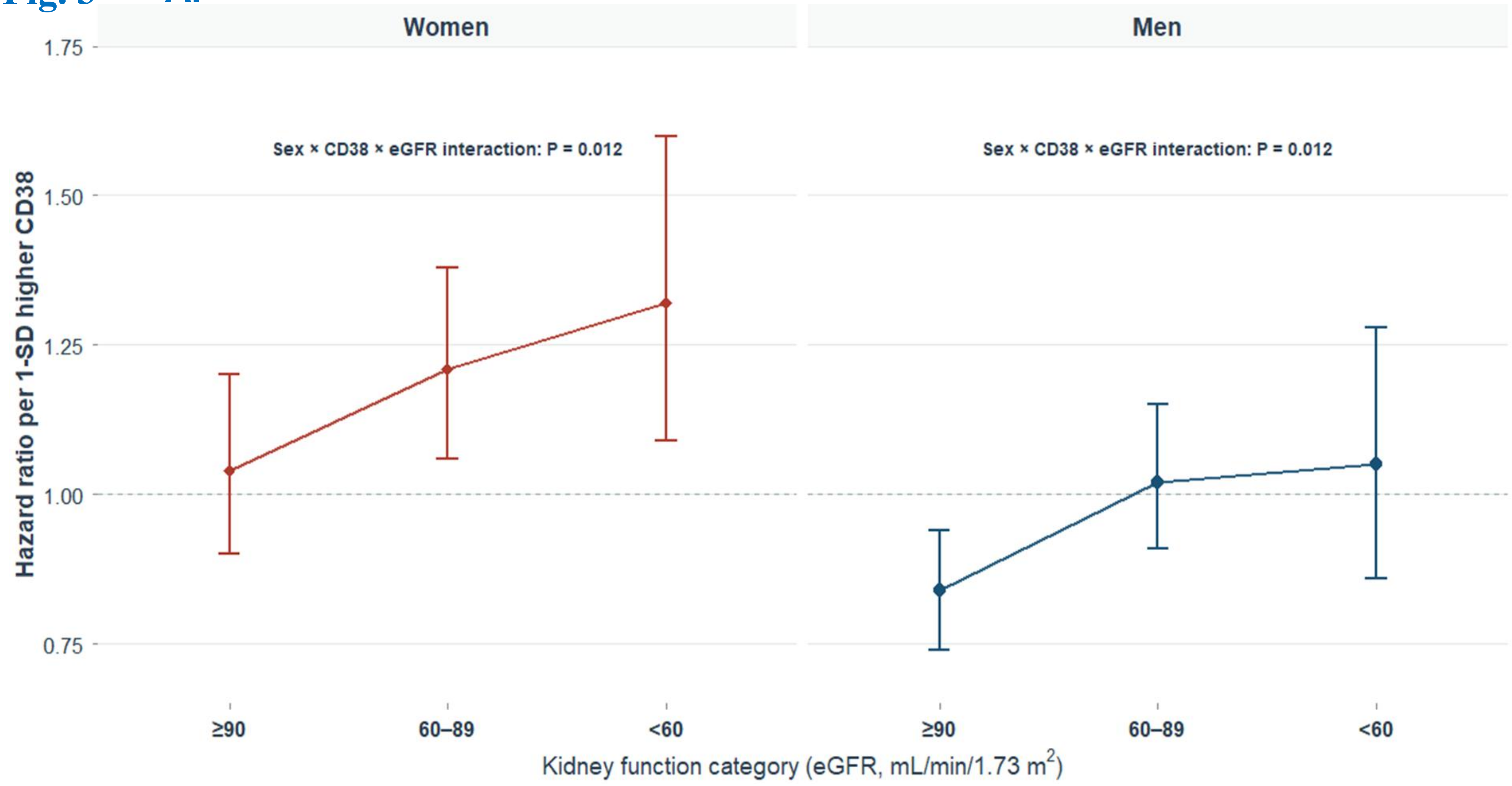
Women
Men
1.75
1.50
1.25
1.00
0.75
Hazard ratio per 1-SD higher CD38
Sex × CD38 × eGFR interaction: P = 0.012
Sex × CD38 × eGFR interaction: P = 0.012
≥90
60–89
<60
≥90
60–89
<60
Kidney function category (eGFR, mL/min/1.73 m2)

Fig. 3

B.

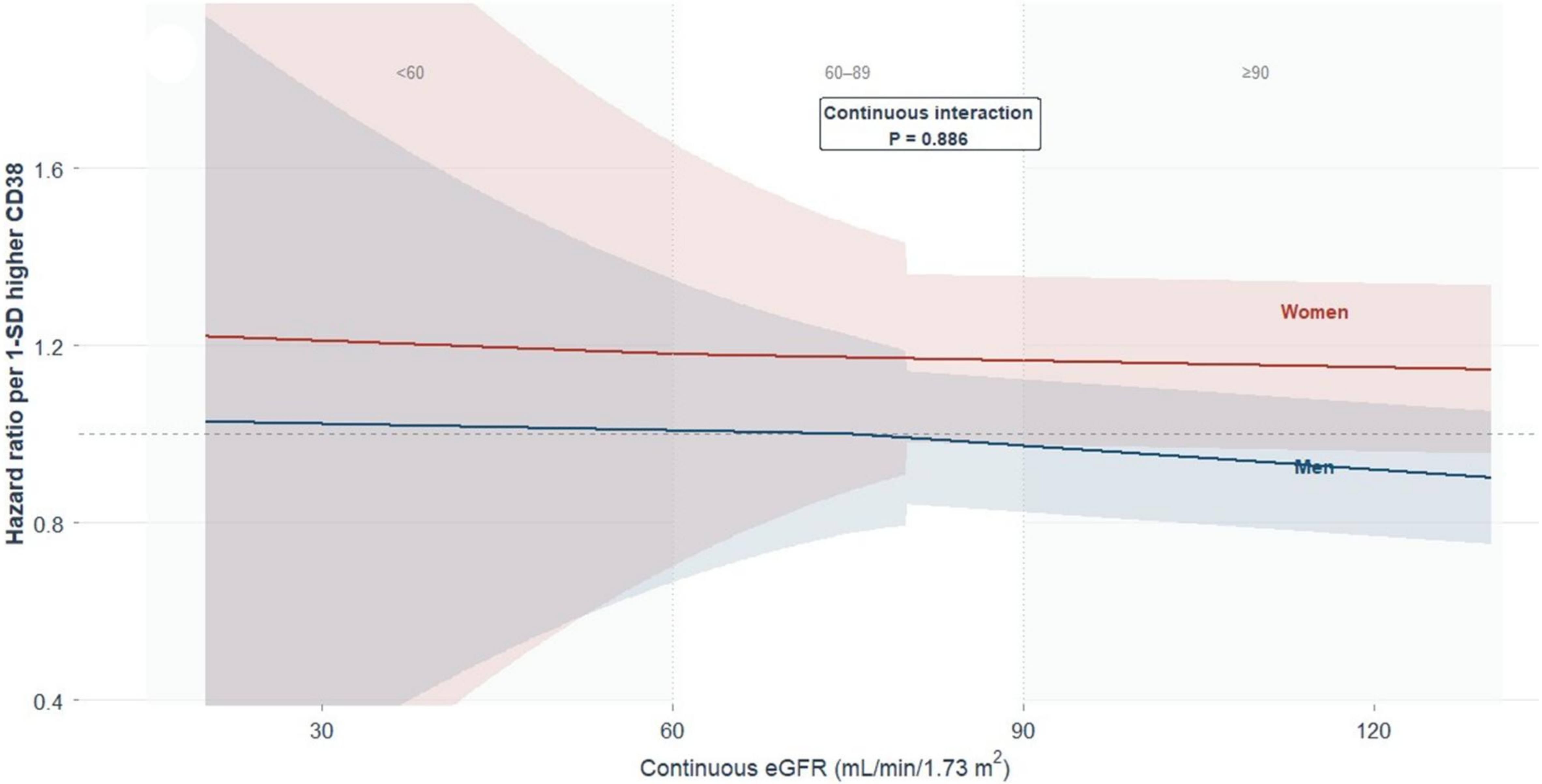

<60
60–89
≥90
Continuous interaction
P = 0.886
Women
Men
Hazard ratio per 1-SD higher CD38
1.6
1.2
0.8
0.4
30
60
90
120
Continuous eGFR (mL/min/1.73 m2)

Fig. 4

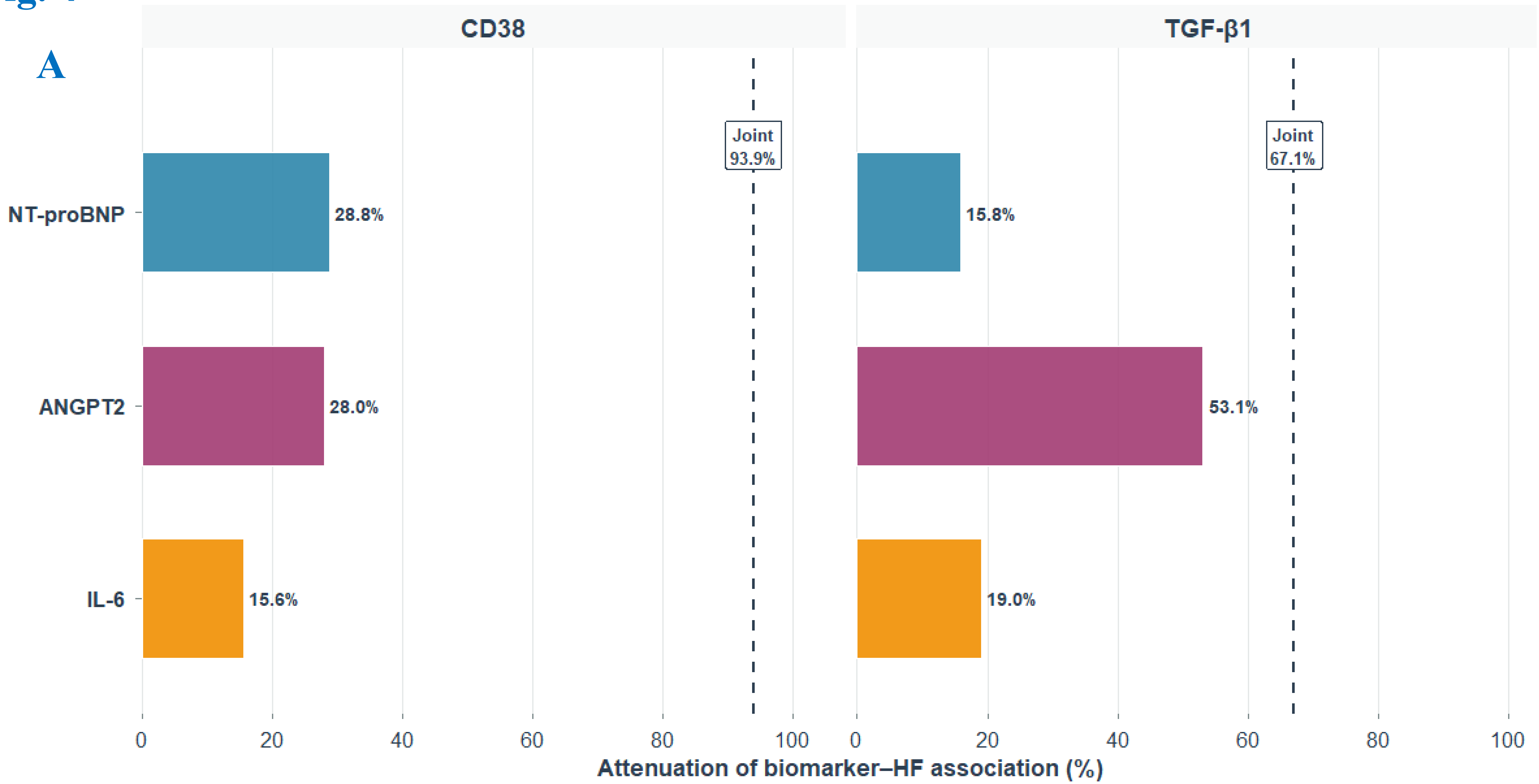
A
CD38
TGF-β1
Joint
93.9%
Joint
67.1%
NT-proBNP
ANGPT2
IL-6
28.8%
28.0%
15.6%
15.8%
53.1%
19.0%
0
20
40
60
80
100
Attenuation of biomarker–HF association (%)

Fig. 4

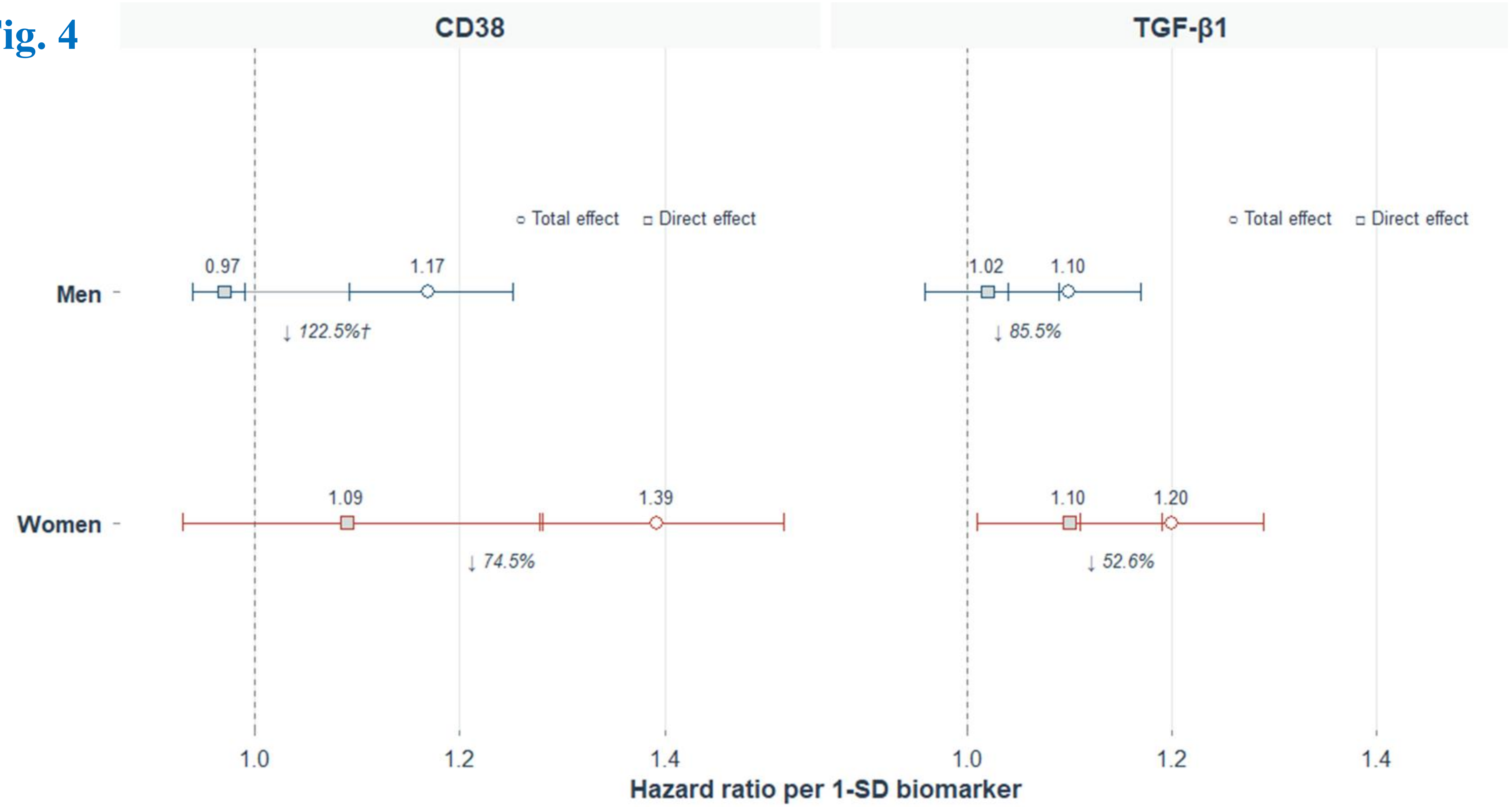

CD38
TGF-β1
Total effect
Direct effect
Men
Women
0.97
1.17
↓ 122.5%†
1.09
1.39
↓ 74.5%
1.02
1.10
↓ 85.5%
1.10
1.20
↓ 52.6%
1.0
1.2
1.4
Hazard ratio per 1-SD biomarker

Fig. 4

(C)

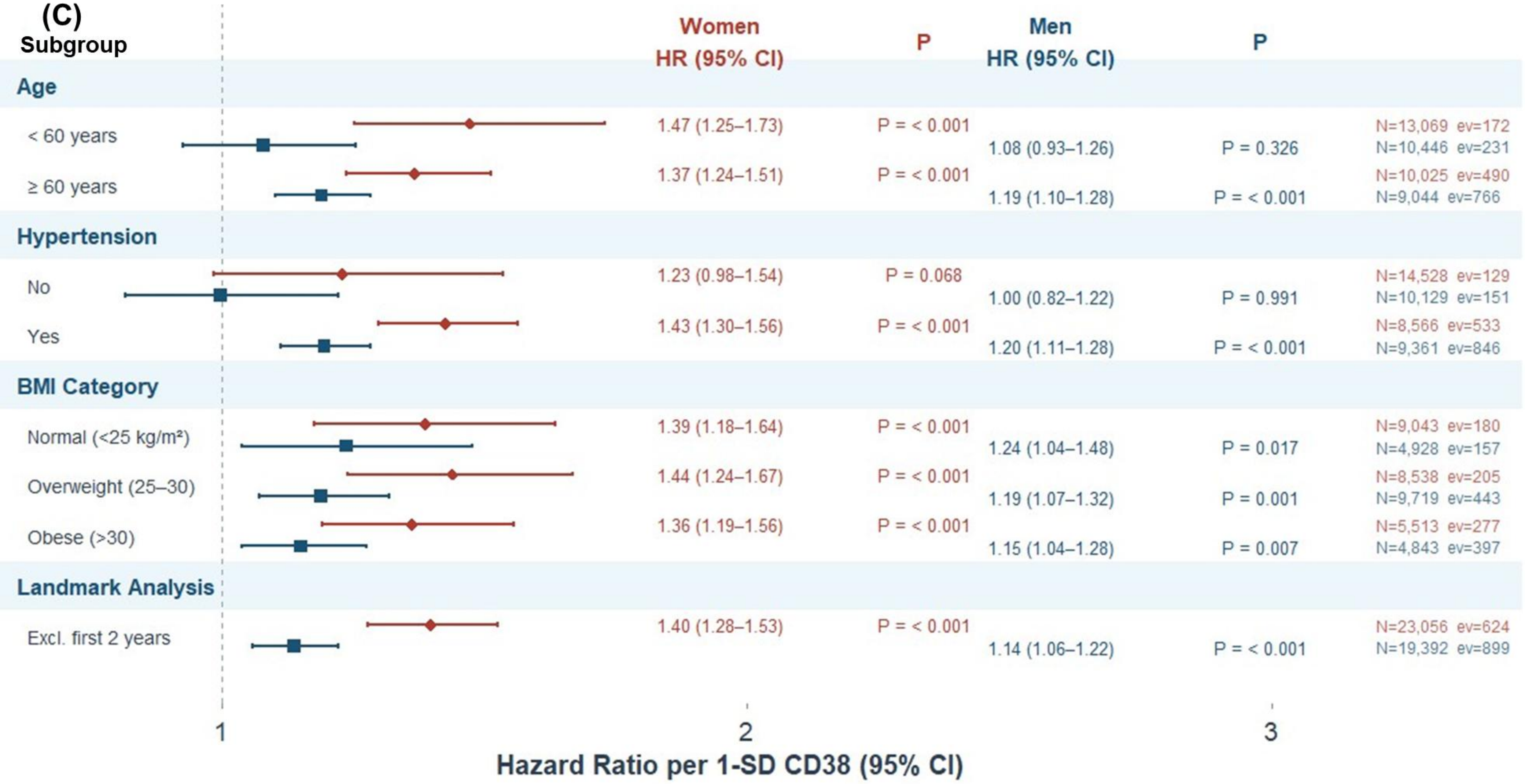

Subgroup
Women HR (95% CI)
P
Men HR (95% CI)
P
Age
< 60 years
1.47 (1.25–1.73)
P = < 0.001
1.08 (0.93–1.26)
P = 0.326
N=13,069 ev=172
N=10,446 ev=231
≥ 60 years
1.37 (1.24–1.51)
P = < 0.001
1.19 (1.10–1.28)
P = < 0.001
N=10,025 ev=490
N=9,044 ev=766
Hypertension
No
1.23 (0.98–1.54)
P = 0.068
1.00 (0.82–1.22)
P = 0.991
N=14,528 ev=129
N=10,129 ev=151
Yes
1.43 (1.30–1.56)
P = < 0.001
1.20 (1.11–1.28)
P = < 0.001
N=8,566 ev=533
N=9,361 ev=846
BMI Category
Normal (<25 kg/m²)
1.39 (1.18–1.64)
P = < 0.001
1.24 (1.04–1.48)
P = 0.017
N=9,043 ev=180
N=4,928 ev=157
Overweight (25–30)
1.44 (1.24–1.67)
P = < 0.001
1.19 (1.07–1.32)
P = 0.001
N=8,538 ev=205
N=9,719 ev=443
Obese (>30)
1.36 (1.19–1.56)
P = < 0.001
1.15 (1.04–1.28)
P = 0.007
N=5,513 ev=277
N=4,843 ev=397
Landmark Analysis
Excl. first 2 years
1.40 (1.28–1.53)
P = < 0.001
1.14 (1.06–1.22)
P = < 0.001
N=23,056 ev=624
N=19,392 ev=899
1
2
3
Hazard Ratio per 1-SD CD38 (95% CI)

Fig. 4

D

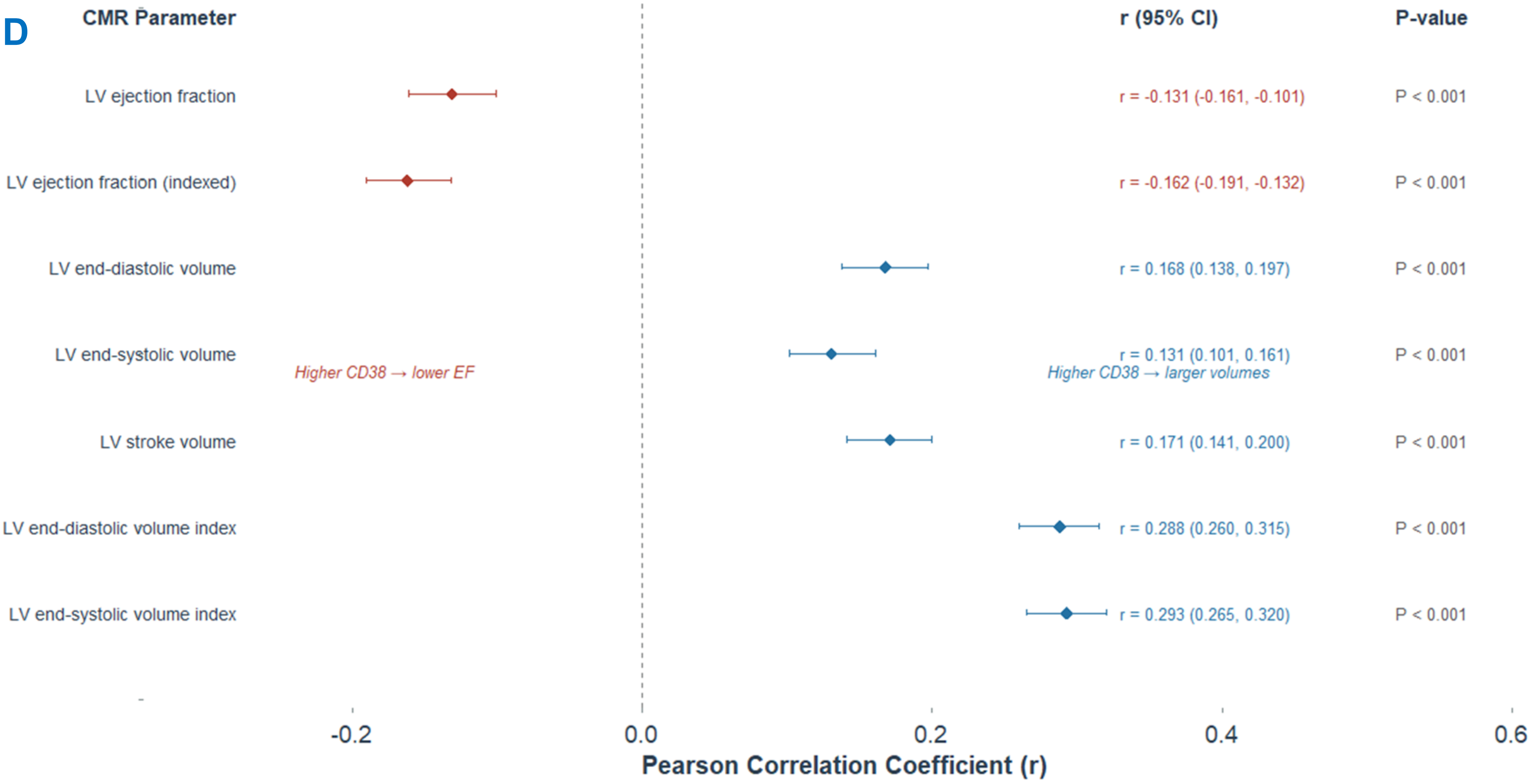
CMR Parameter
r (95% CI)
P-value
LV ejection fraction
r = -0.131 (-0.161, -0.101)
P < 0.001
LV ejection fraction (indexed)
r = -0.162 (-0.191, -0.132)
P < 0.001
LV end-diastolic volume
r = 0.168 (0.138, 0.197)
P < 0.001
LV end-systolic volume
r = 0.131 (0.101, 0.161)
P < 0.001
Higher CD38 → lower EF
Higher CD38 → larger volumes
LV stroke volume
r = 0.171 (0.141, 0.200)
P < 0.001
LV end-diastolic volume index
r = 0.288 (0.260, 0.315)
P < 0.001
LV end-systolic volume index
r = 0.293 (0.265, 0.320)
P < 0.001
-0.2
0.0
0.2
0.4
0.6
Pearson Correlation Coefficient (r)

Fig. 4 E.

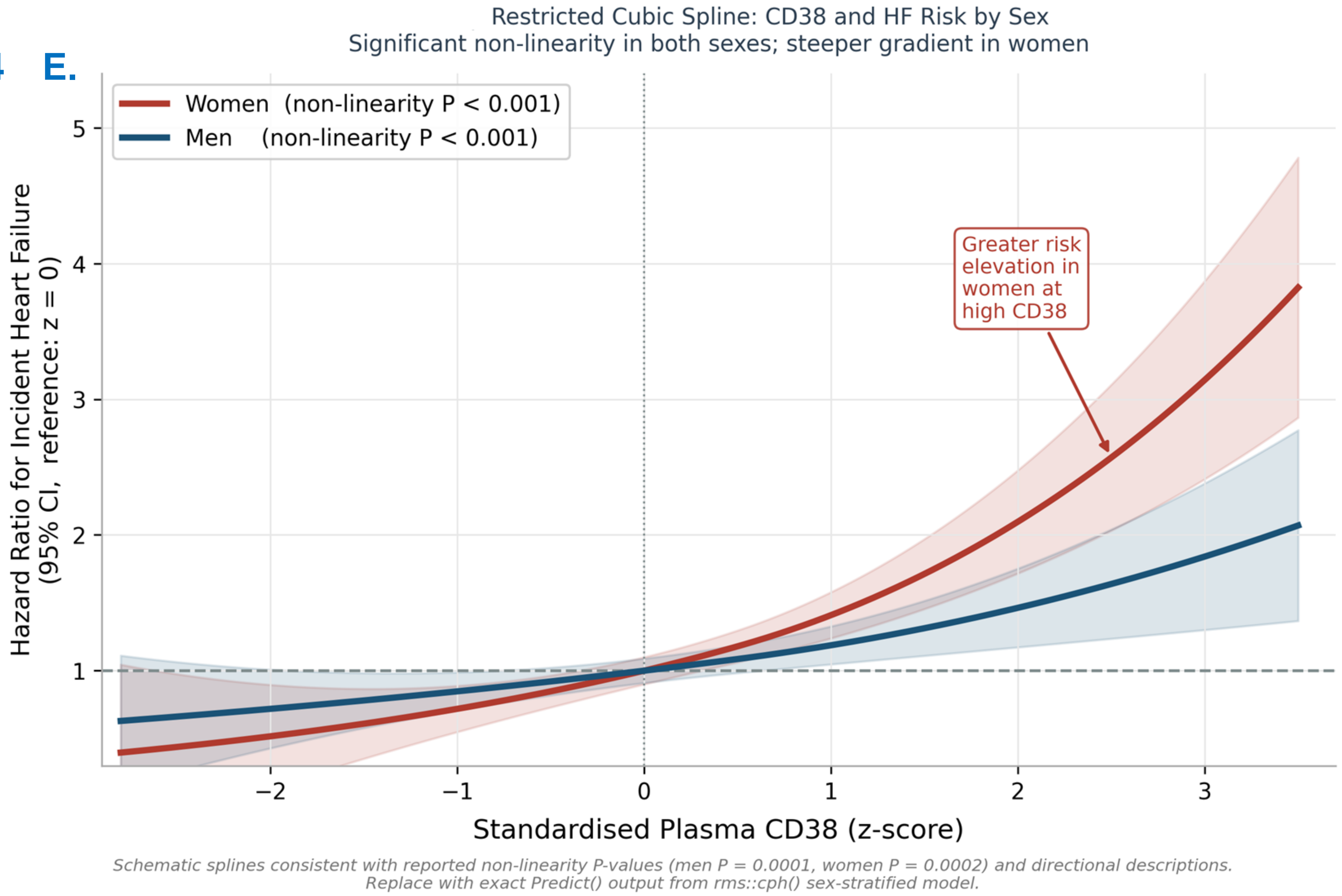
Restricted Cubic Spline: CD38 and HF Risk by Sex
Significant non-linearity in both sexes; steeper gradient in women
Women (non-linearity P < 0.001)
Men (non-linearity P < 0.001)
Greater risk elevation in women at high CD38
Hazard Ratio for Incident Heart Failure (95% CI, reference: z = 0)
5
4
3
2
1
−2
−1
0
1
2
3
Standardised Plasma CD38 (z-score)
Schematic splines consistent with reported non-linearity P-values (men P = 0.0001, women P = 0.0002) and directional descriptions.
Replace with exact Predict() output from rms::cph() sex-stratified model.

Fig. 5

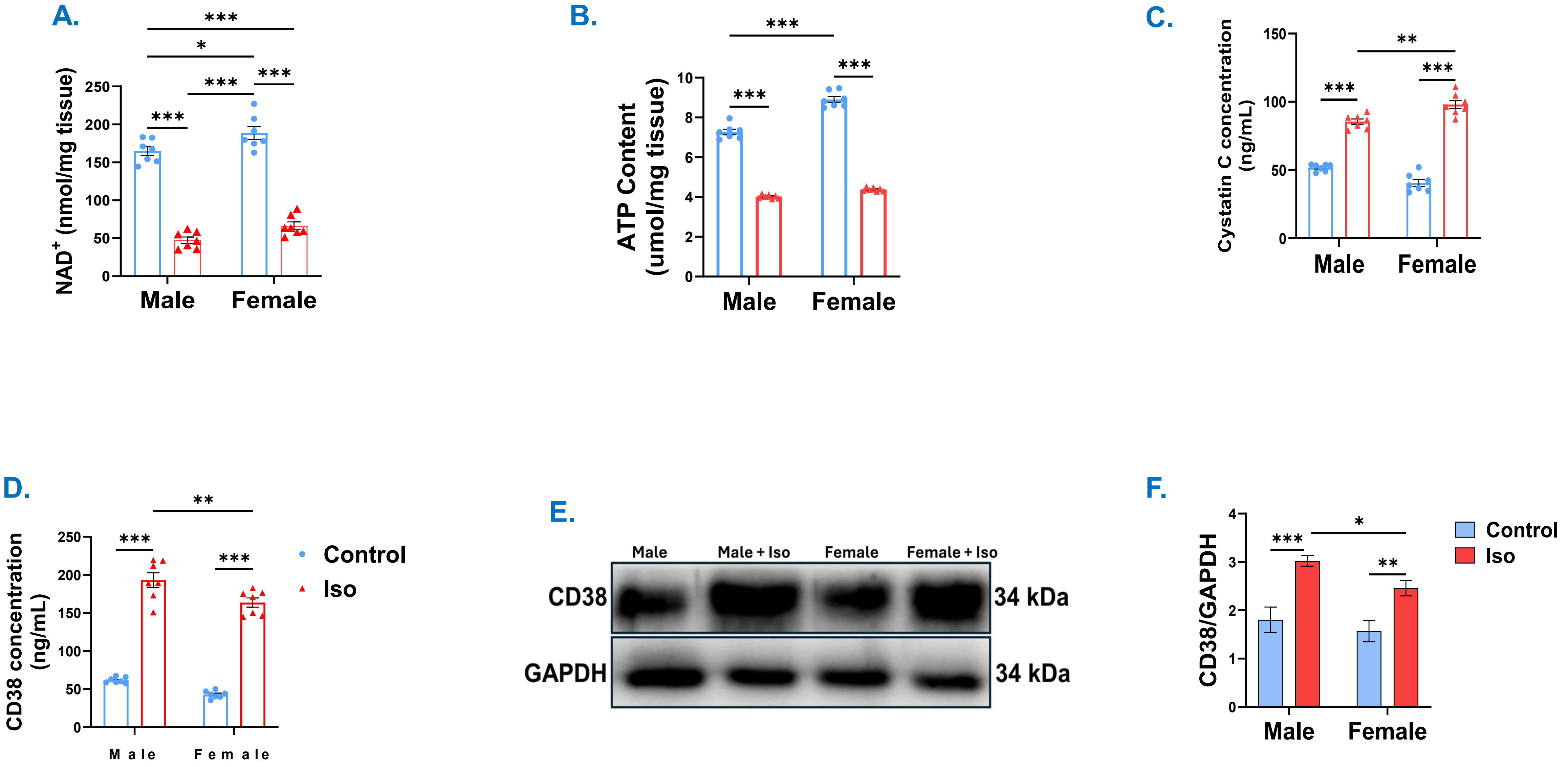

A.
NAD+ (nmol/mg tissue)
250
200
150
100
50
0
***
*
***
***
***
Male
Female
B.
ATP Content
(umol/mg tissue)
10
8
6
4
2
0
***
***
***
Male
Female
C.
Cystatin C concentration
(ng/mL)
150
100
50
0
**
***
***
Male
Female
D.
CD38 concentration
(ng/mL)
250
200
150
100
50
0
**
***
***
Male
Female
Control
Iso
E.
Male
Male + Iso
Female
Female + Iso
CD38
GAPDH
34 kDa
34 kDa
F.
CD38/GAPDH
4
3
2
1
0
***
*
**
Male
Female
Control
Iso

Fig. 5

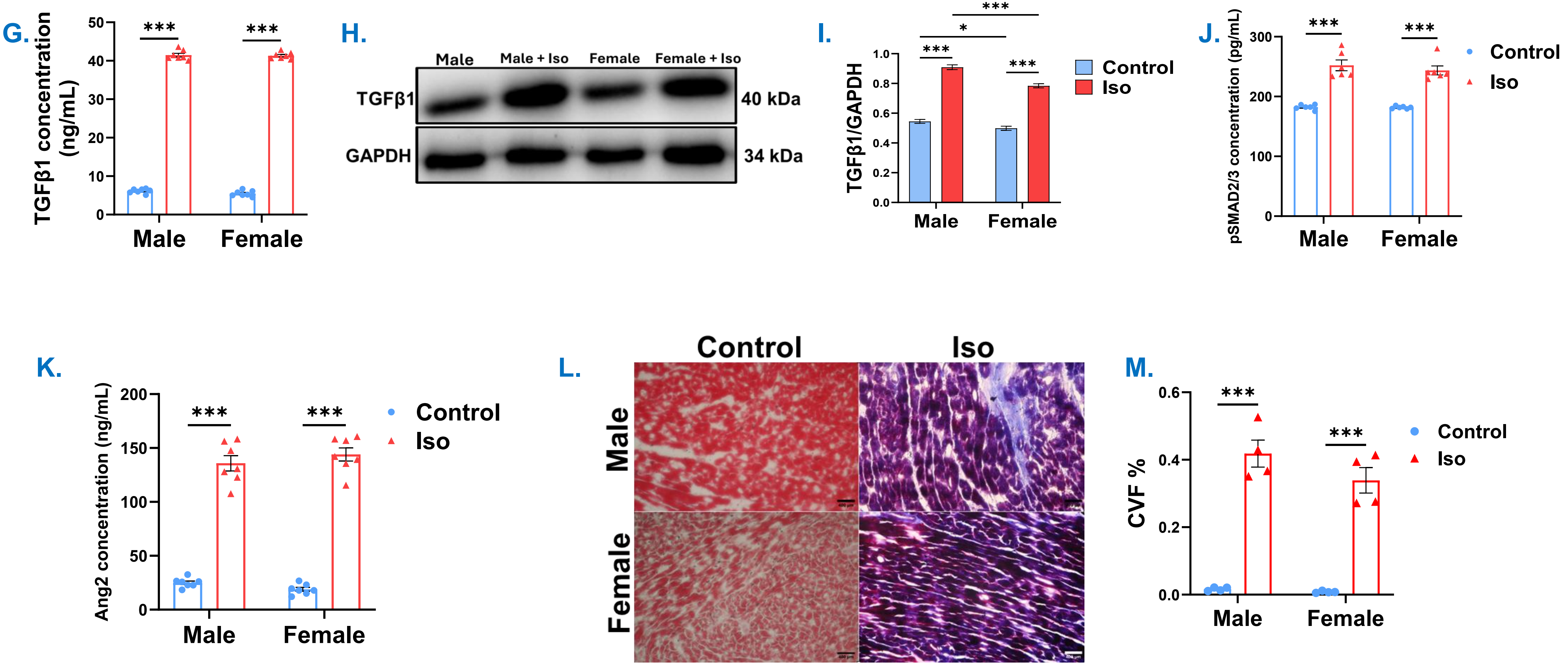

G.
TGFβ1 concentration (ng/mL)
0
10
20
30
40
50
***
***
Male
Female
H.
Male
Male + Iso
Female
Female + Iso
TGFβ1
40 kDa
GAPDH
34 kDa
I.
TGFβ1/GAPDH
0.0
0.2
0.4
0.6
0.8
1.0
***
*
***
***
Male
Female
Control
Iso
J.
pSMAD2/3 concentration (pg/mL)
0
100
200
300
***
***
Male
Female
Control
Iso
K.
Ang2 concentration (ng/mL)
0
50
100
150
200
***
***
Male
Female
Control
Iso
L.
Control
Iso
Male
Female
M.
CVF %
0.0
0.2
0.4
0.6
***
***
Male
Female
Control
Iso

Fig. 6

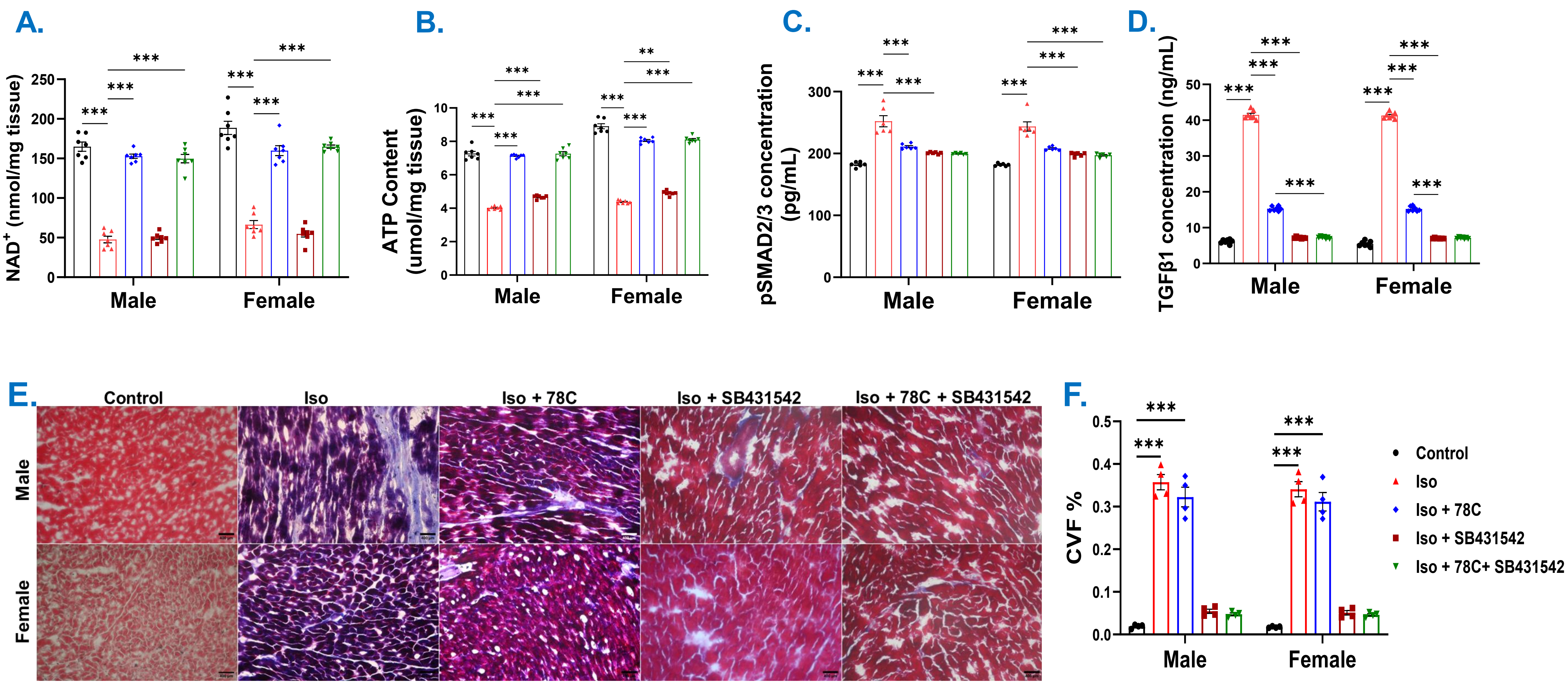

A.
NAD+ (nmol/mg tissue)
Male
Female
B.
ATP Content (umol/mg tissue)
Male
Female
C.
pSMAD2/3 concentration (pg/mL)
Male
Female
D.
TGFβ1 concentration (ng/mL)
Male
Female
E.
Control
Iso
Iso + 78C
Iso + SB431542
Iso + 78C + SB431542
Male
Female
F.
CVF %
Male
Female
Control
Iso
Iso + 78C
Iso + SB431542
Iso + 78C+ SB431542

Fig. 6

G.

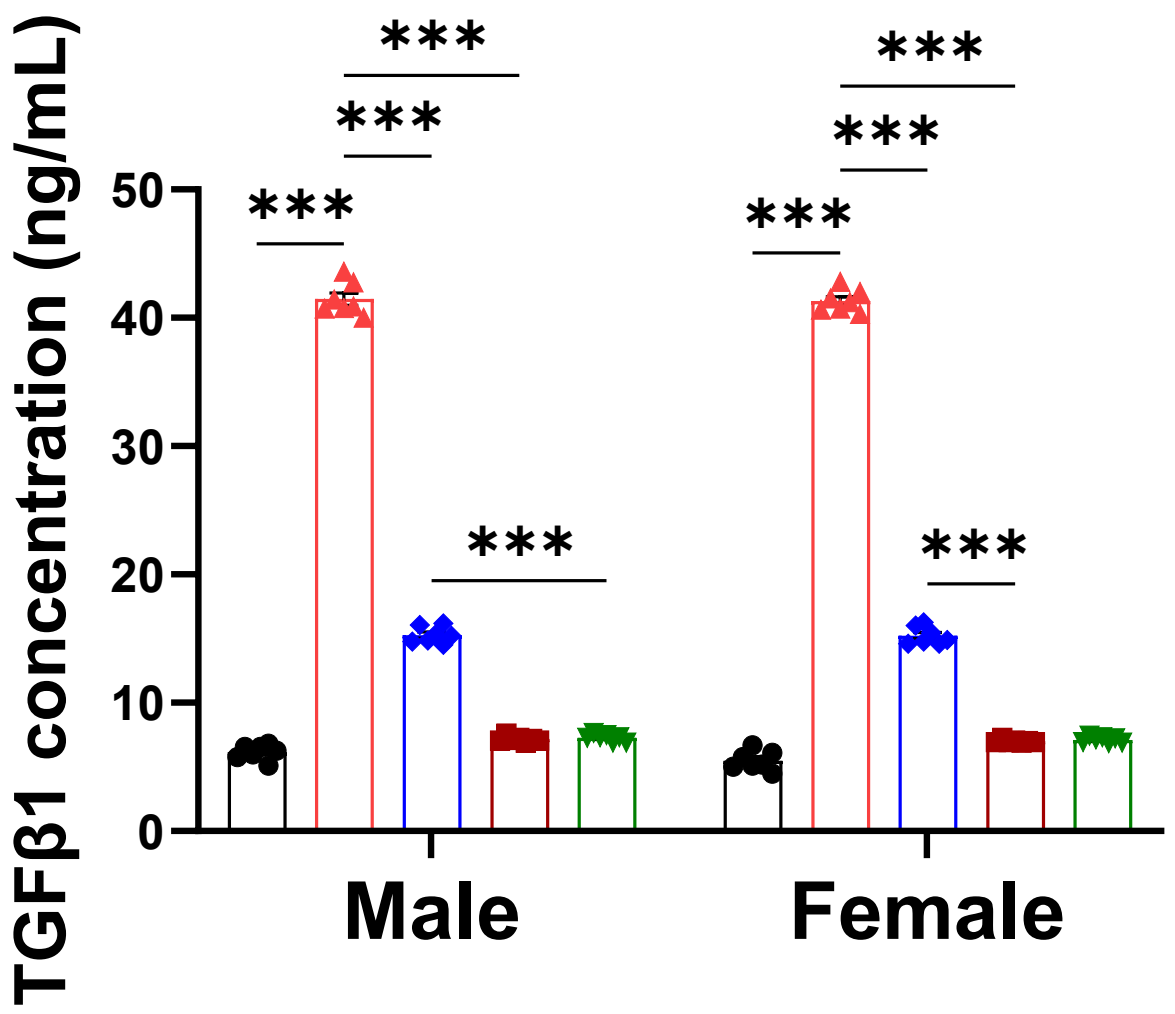
TGFβ1 concentration (ng/mL)
50
40
30
20
10
0
***
***
***
***
***
***
***
***
Male
Female

H.

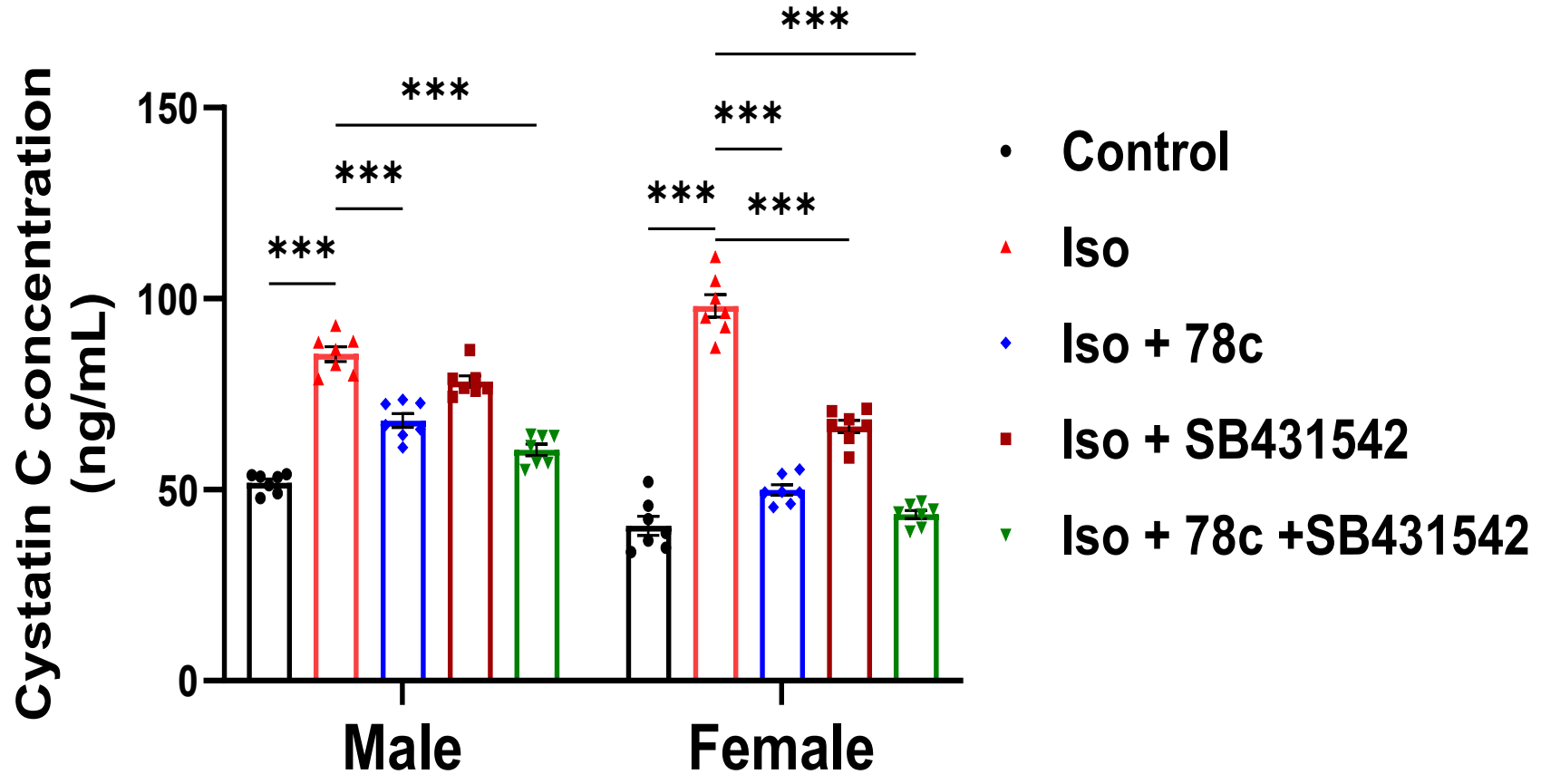
Cystatin C concentration (ng/mL)
150
100
50
0
***
***
***
***
***
***
***
Male
Female
Control
Iso
Iso + 78c
Iso + SB431542
Iso + 78c +SB431542

Fig S1.

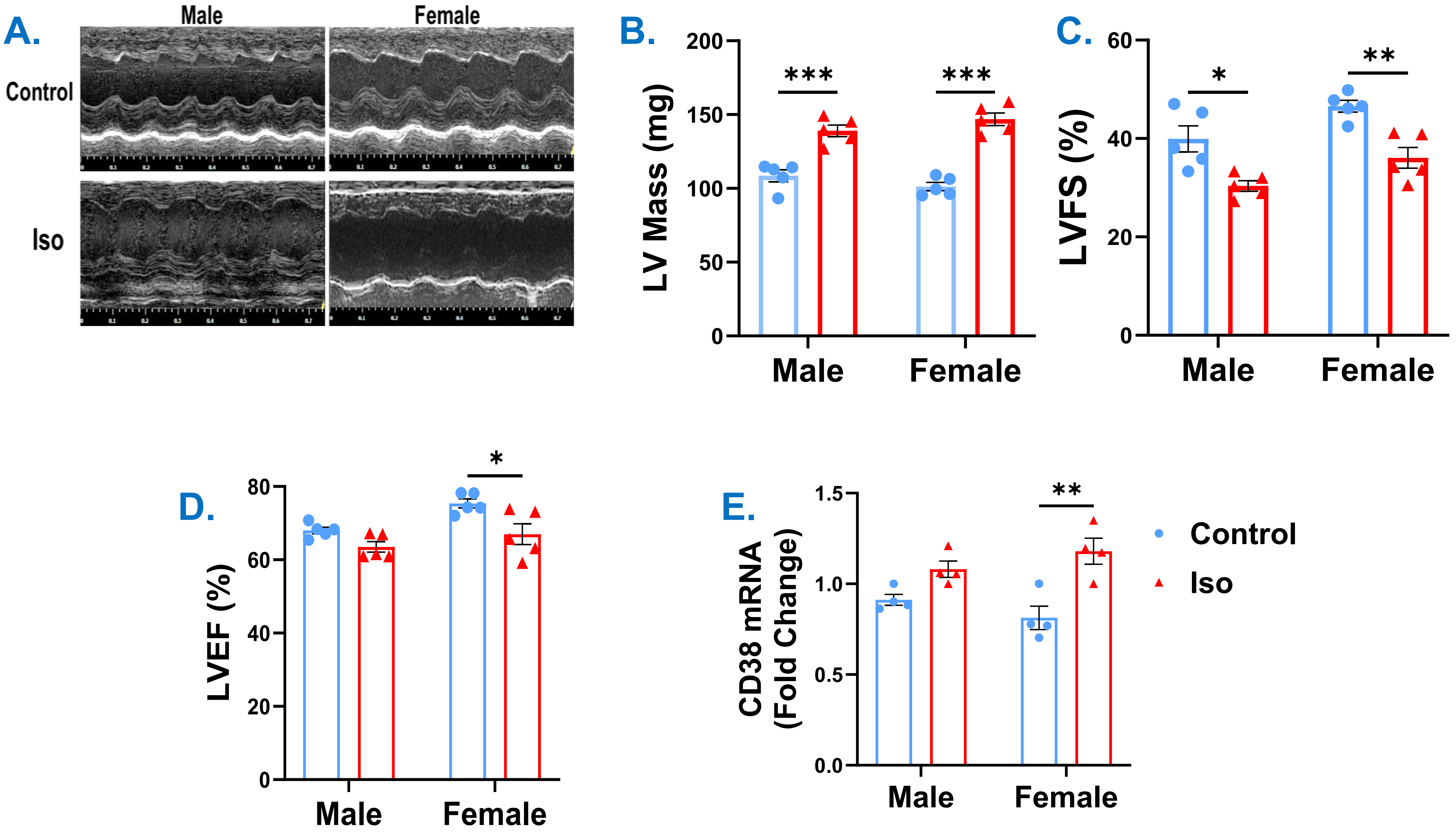
A.
Male
Female
Control
Iso
B.
LV Mass (mg)
200
150
100
50
0
***
***
Male
Female
C.
LVFS (%)
60
40
20
0
*
**
Male
Female
D.
LVEF (%)
80
60
40
20
0
*
Male
Female
E.
CD38 mRNA (Fold Change)
1.5
1.0
0.5
0.0
**
Male
Female
Control
Iso

Fig S2.

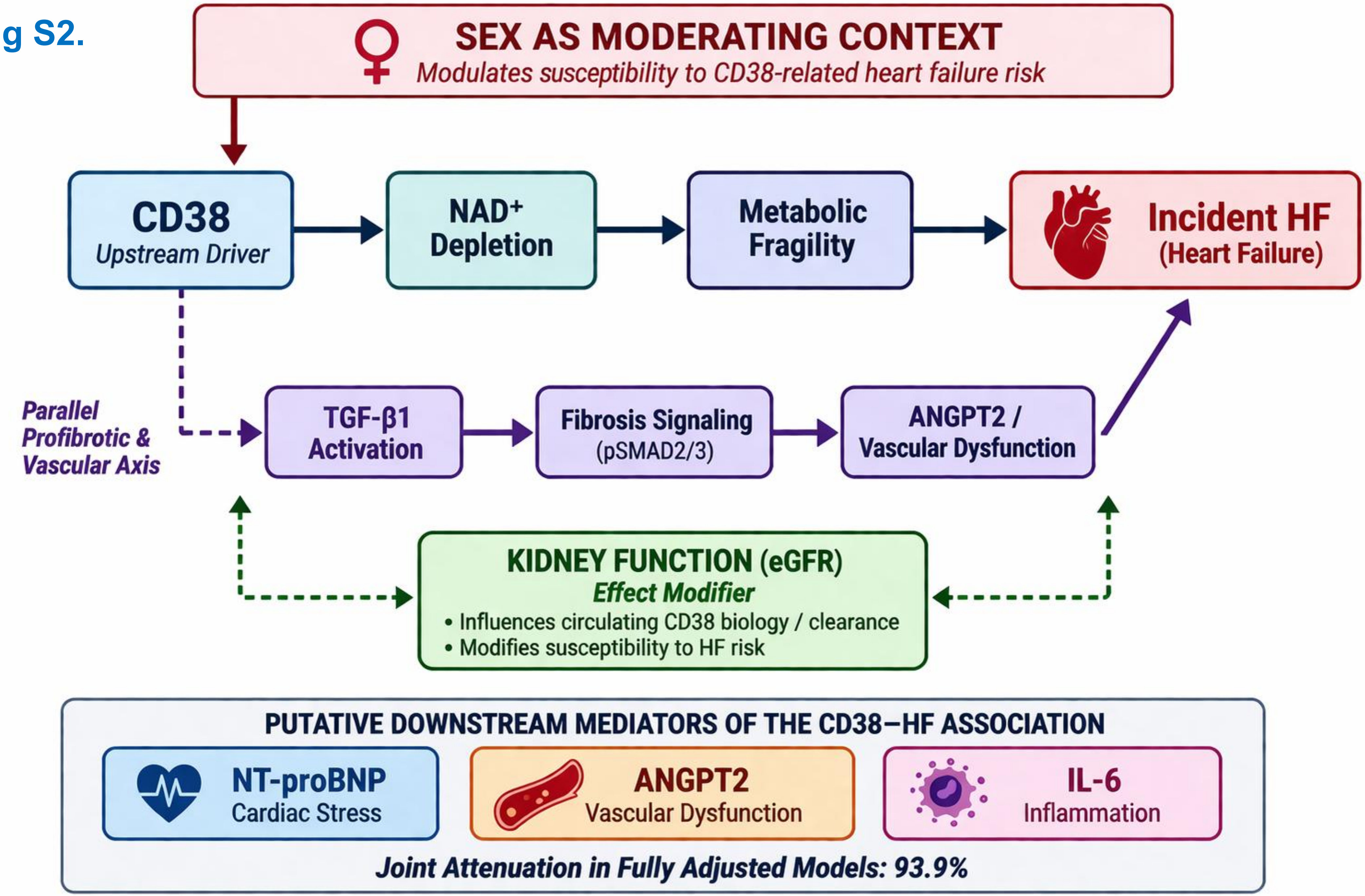
SEX AS MODERATING CONTEXT
Modulates susceptibility to CD38-related heart failure risk
CD38
Upstream Driver
NAD+ Depletion
Metabolic Fragility
Incident HF
(Heart Failure)
Parallel Profibrotic & Vascular Axis
TGF-β1 Activation
Fibrosis Signaling
(pSMAD2/3)
ANGPT2 / Vascular Dysfunction
KIDNEY FUNCTION (eGFR)
Effect Modifier
• Influences circulating CD38 biology / clearance
• Modifies susceptibility to HF risk
PUTATIVE DOWNSTREAM MEDIATORS OF THE CD38–HF ASSOCIATION
NT-proBNP
Cardiac Stress
ANGPT2
Vascular Dysfunction
IL-6
Inflammation
Joint Attenuation in Fully Adjusted Models: 93.9%

Graphical Abstract

CD38 SEX PARADOX: SUMMARY SCHEMATIC

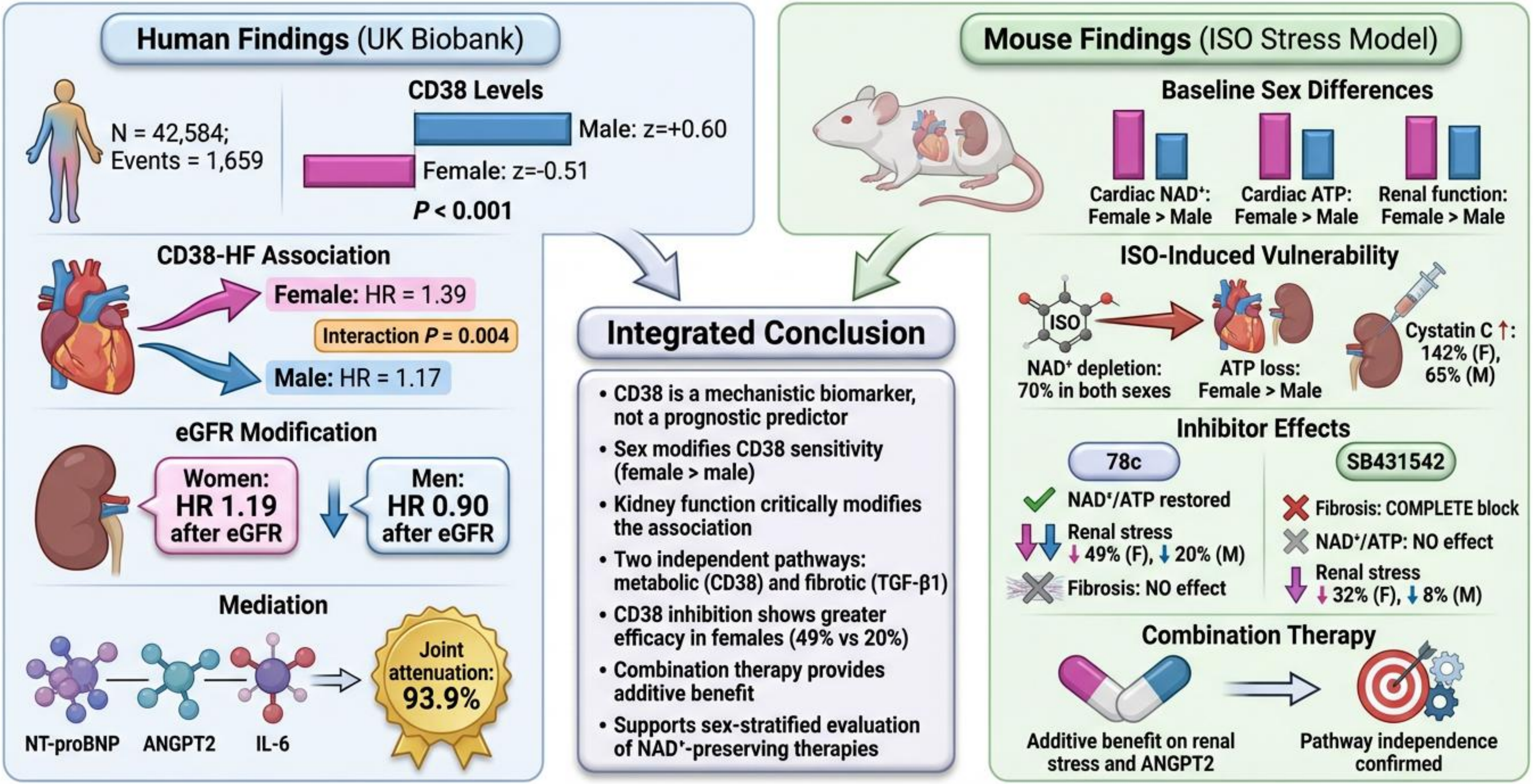

Supplement: Supplementary file 1 [file 13293_2026_924_MOESM3_ESM.pdf]
