# Supplementary material for: Circulating CD38 shows stronger association with incident heart failure in women despite lower levels: sex differences and kidney-function context
Source: Biol Sex Differ. 2026 Jun 4;17:144. doi: 10.1186/s13293-026-00924-7 (PMC13449966; doi:10.1186/s13293-026-00924-7)
Supplement: Supplementary file 2 [file 13293_2026_924_MOESM1_ESM.docx]

**Supplementary methods**

**Supplementary Methods 1: Detailed Covariate Definitions**

**Demographic and Clinical Variables**

Age was measured in years at baseline assessment. Sex was recorded as male or female. Body mass index (BMI) was calculated as weight (kg) divided by height squared (m²). Systolic blood pressure (mmHg) was recorded as the mean of two automated readings. Smoking status was categorized as never, former, or current smoker. Alcohol consumption status was categorized as never, former, or current drinker.

**Comorbidities**

Hypertension was defined as self-reported physician diagnosis, use of antihypertensive medication, or measured systolic blood pressure ≥140 mmHg or diastolic blood pressure ≥90 mmHg. Diabetes mellitus was defined as self-reported physician diagnosis, use of glucose-lowering medication, or HbA1c ≥6.5%. Hyperlipidemia was defined as self-reported physician diagnosis or use of lipid-lowering medication.

**Circulating Biomarkers**

N-terminal pro-B-type natriuretic peptide (NT-proBNP), interleukin-6 (IL-6), angiopoietin-2 (ANGPT2), cardiac troponin I (TNNI3), and C-reactive protein (CRP) were measured using the Olink Explore 3072 platform and expressed as Normalized Protein Expression (NPX) values. All values were standardized to z-scores for regression analyses. Blood biomarkers from the UK Biobank biochemistry panel included low-density lipoprotein cholesterol (LDL-C), high-density lipoprotein cholesterol (HDL-C), triglycerides, glycated hemoglobin (HbA1c), alanine aminotransferase (ALT), aspartate aminotransferase (AST), serum urate, and albumin, measured using standard biochemical assays in the UK Biobank core laboratory.

**Medications**

Medication use was ascertained from self-reported treatment codes at baseline. Cardiovascular medications included statins, angiotensin-converting enzyme inhibitors (ACEi), angiotensin receptor blockers (ARB), beta-blockers, calcium channel blockers (CCB), and diuretics. Antiplatelet agents and metformin use were also recorded. Polypharmacy was defined as concurrent use of five or more medications from any therapeutic class.

**Supplementary Methods 2: Cardiac Magnetic Resonance Imaging**

A subset of participants (n = 4,175) underwent cardiac magnetic resonance (CMR) imaging as part of the UK Biobank imaging enhancement. CMR scans were performed using a clinical wide-bore 1.5 Tesla scanner (MAGNETOM Aera, Syngo Platform VD13A, Siemens Healthcare, Erlangen, Germany). Left ventricular (LV) structure and function were assessed using balanced steady-state free precession cine imaging in standard long-axis and short-axis views covering the entire LV. Left ventricular end-diastolic volume (LVEDV), left ventricular end-systolic volume (LVESV), and left ventricular ejection fraction (LVEF) were derived from short-axis cine images using semi-automated threshold-based segmentation with manual correction. Volumes were indexed to body surface area (calculated using the Mosteller formula) to generate LVEDV index and LVESV index. All measurements were performed by trained observers following standardized protocols at a central core laboratory.

Associations between standardized biomarker levels (CD38, TGF-β1) and cardiac structural parameters (LVEF, LVEDV, LVESV, LVEDV index, LVESV index) were assessed using Pearson correlation coefficients. Correlations were computed separately for males and females and tested for significant sex differences using Fisher's z-transformation.

**Supplementary Methods 3: Biomarker Interaction Testing**

To test whether CD38 and TGF-β1 exhibit synergistic or antagonistic effects on heart failure risk, we included a multiplicative interaction term (z_CD38 × z_TGFB1) in Cox models adjusted for age, sex, BMI, hypertension, and diabetes. The significance of the interaction was assessed using the Wald test from the Cox model coefficient for the interaction term.

**Supplementary Methods 4: Subgroup and Sensitivity Analyses**

**Subgroup Analyses**

Subgroup analyses were performed stratified by age (<60 vs ≥60 years), BMI category (normal weight <25, overweight 25-29.9, obese ≥30 kg/m²), and hypertension status. Within each subgroup defined by sex and clinical characteristic, Cox models were fit using the base adjustment covariates (age, BMI, hypertension, diabetes). Effect modification was assessed using multiplicative interaction terms, with statistical significance evaluated using Wald tests.

We examined whether baseline levels of cardiac stress and inflammatory biomarkers modified the CD38-heart failure association by stratifying participants into tertiles of NT-proBNP, IL-6, and ANGPT2, and testing biomarker × tertile interactions. We also tested effect modification by age group (<60 vs ≥60 years). Multiple testing adjustment was applied using the Benjamini-Hochberg false discovery rate (FDR) procedure. Interactions with FDR-adjusted p-values <0.05 were considered statistically significant.

**Landmark Analyses**

To address potential reverse causation, we performed landmark analyses excluding participants who developed heart failure during the first 2 years and first 5 years of follow-up. Cox models were re-fit in the remaining cohort using the same adjustment strategy as primary analyses, with follow-up time recalculated from the landmark time point (2-year or 5-year mark). Hazard ratios and 95% confidence intervals were estimated separately for males and females.

**Supplementary Methods 5: Model Diagnostics**

**Proportional Hazards Assumption**

The proportional hazards assumption was assessed for CD38 and other biomarkers using scaled Schoenfeld residuals and tested formally with chi-square tests. For each Cox model, we plotted the scaled Schoenfeld residuals against time and tested the null hypothesis that the slope is zero. For biomarkers violating the proportional hazards assumption (P < 0.05 on the chi-square test), time-varying coefficients were examined graphically by plotting smoothed coefficient estimates over follow-up time and reported descriptively in results.

**Non-linearity Assessment**

Non-linearity of the CD38-heart failure association was assessed using restricted cubic splines with three knots placed at the 10th, 50th, and 90th percentiles of the CD38 distribution. The null hypothesis of linearity was tested using likelihood ratio tests comparing models with and without the spline terms (degrees of freedom = 2). Analyses were performed separately in males and females to assess whether the shape of the dose-response relationship differed by sex. Spline curves were plotted with 95% confidence intervals to visualize the functional form of the association.

**Supplementary Methods 6: Predictive Discrimination and Reclassification**

**Model Construction**

To evaluate the incremental prognostic value of CD38 beyond established risk factors, we constructed two risk prediction models. The base model included age, sex, BMI, eGFR, hypertension, diabetes, NT-proBNP, and ANGPT2. The extended model added CD38 to the base model. Both models were fit using Cox proportional hazards regression in the full cohort.

**Discrimination Metrics**

Predictive performance was assessed at 5 years using time-dependent area under the receiver operating characteristic curve (AUC), accounting for censoring using inverse probability of censoring weighting (IPCW). IPCW weights were calculated by fitting a censoring model (Cox model for time to censoring as a function of baseline covariates) and using the inverse of the predicted survival probability at each event time. The difference in AUC between the base and extended models (ΔAUC) was tested using DeLong's method for comparing correlated ROC curves.

**Reclassification Metrics**

Reclassification performance was evaluated using two metrics. The Integrated Discrimination Improvement (IDI) quantifies the improvement in average predicted risk, calculated as the difference in discrimination slopes between models: IDI = [P(events|extended) - P(events|base)] - [P(non-events|extended) - P(non-events|base)], where P denotes mean predicted probability. The continuous Net Reclassification Improvement (NRI) assesses the net proportion of participants correctly reclassified, defined as the proportion of events with increased predicted risk minus the proportion of non-events with increased predicted risk when adding CD38 to the base model.

Bootstrap resampling with 1,000 iterations was used to estimate 95% confidence intervals for AUC, ΔAUC, IDI, and NRI. For each bootstrap sample, the model was refit and discrimination metrics were calculated. Confidence intervals were constructed using the percentile method (2.5th and 97.5th percentiles of the bootstrap distribution).

**Supplementary Methods 7: Detailed Preclinical Methods**

**Plasma ELISA Measurements**

Plasma concentrations of CD38, TGF-β1, angiopoietin-2 (ANGPT2), phospho-SMAD2/3 (pSMAD2/3), and cystatin C were measured using enzyme-linked immunosorbent assays according to manufacturer protocols:

**Cystatin C:** Mouse Cystatin C SimpleStep ELISA Kit (Abcam, Cambridge, UK; catalog # ab201280)

**pSMAD2/3**: Mouse Phosphorylated Smad Family Member 2/3 ELISA Kit (Byabscience, Beijing, China; catalog # BY-EM223321)

**CD38**: Mouse CD38 Molecule ELISA Detection Kit (Jianglai Biological, Shanghai, China; catalog # JL54370-96T)

**ANGPT2**: Mouse Angiopoietin-2 ELISA Kit (Elabscience, Wuhan, China; catalog # E-EL-M3101)

**TGF-βI**: Mouse Transforming Growth Factor Beta Induced Protein ELISA Detection Kit (Jianglai Biological, Shanghai, China; catalog # JL29328-96T)

Plasma samples were thawed on ice and diluted according to manufacturer recommendations. Standards were prepared by serial dilution of recombinant protein standards provided in each kit. Samples and standards were added to pre-coated 96-well plates and incubated for the specified time at room temperature or 37°C. After washing, detection antibodies were added, followed by substrate solution. The reaction was stopped with stop solution, and absorbance was read at 450 nm (with 570 nm reference wavelength) using a microplate reader. Concentrations were calculated from standard curves using four-parameter logistic regression. All samples were run in duplicate with intra-assay coefficients of variation <10% and inter-assay coefficients of variation <15%. ELISA measurements were performed in both Phase 1 and Phase 2 experiments.

**Western Blotting Protocol**

Western blot analysis was performed in Phase 1 to characterize cardiac tissue expression of CD38 and TGF-β1 at baseline and following ISO treatment. Cardiac tissue lysates were prepared by homogenizing frozen tissue (~30 mg) in RIPA lysis buffer (VICMED, China) supplemented with protease inhibitor cocktail (VICMED, China) and phosphatase inhibitors (VICMED, China) at a ratio of 1:10 (weight:volume). Homogenization was performed on ice using a handheld homogenizer at maximum speed for 30 seconds. After incubation on ice for 30 minutes with intermittent vortexing every 10 minutes, lysates were centrifuged at 12,000 × g for 15 minutes at 4°C, and supernatants were collected. Protein concentration was determined using the BCA Protein Assay Kit (Beyotime, China; catalog # P0012S) according to manufacturer instructions, with bovine serum albumin as the standard.

Equal amounts of protein (30 μg per lane) were mixed with 4× SDS sample buffer containing β-mercaptoethanol and heated at 95°C for 5 minutes. Samples were separated by 10% SDS-PAGE at 120 V for approximately 90 minutes and transferred to polyvinylidene difluoride (PVDF) membranes (0.45 μm pore size, Millipore, Burlington, MA, USA) using a wet transfer system at 100 V for 90 minutes at 4°C. Transfer efficiency was confirmed by Ponceau S staining.

Membranes were blocked with 5% non-fat milk in Tris-buffered saline containing 0.1% Tween-20 (TBST) for 1 hour at room temperature with gentle agitation. Membranes were then incubated overnight at 4°C with primary antibodies diluted in 5% bovine serum albumin in TBST: anti-CD38 (catalog # DF6551, 1:1000 dilution; Affinity Biosciences, China), anti-TGF-β1 (catalog # 21898-I-AP, 1:1000 dilution; Proteintech, Rosemont, IL, USA), and anti-GAPDH (catalog # 5174, 1:5000 dilution, loading control; Cell Signaling Technology, Danvers, MA, USA).

After washing three times with TBST (10 minutes each), membranes were incubated with horseradish peroxidase (HRP)-conjugated secondary antibodies (anti-rabbit IgG, catalog # 7074, 1:5000 dilution, Cell Signaling Technology) for 1 hour at room temperature. Following three additional washes with TBST, immunoreactive bands were visualized using an enhanced chemiluminescence (ECL) reagent (Tanon Biotechnology Co., Ltd., Shanghai, China). Chemiluminescent signals were captured using a chemiluminescence imaging system (Tanon Biotechnology Co., Ltd., Shanghai, China) with automatic exposure optimization. Band densities were quantified using ImageJ software (version 1.52a; National Institutes of Health, Bethesda, MD, USA) by measuring integrated density within manually drawn regions of interest. Target protein levels were normalized to GAPDH loading control from the same membrane, and results were expressed as fold-change relative to sex-matched control group.

**Tissue Collection Protocol Details**

At study termination (day 21), mice were weighed and anesthetized with esketamine (100 mg/kg, intraperitoneal injection). Depth of anesthesia was confirmed by absence of toe pinch reflex. Blood was collected via cardiac puncture using a 25-gauge needle into EDTA-coated tubes (1.5 mL microcentrifuge tubes with 10 μL of 0.5 M EDTA). Blood samples were immediately placed on ice and centrifuged at 3,000 × g for 15 minutes at 4°C within 30 minutes of collection. Plasma (supernatant) was carefully transferred to fresh tubes without disturbing the buffy coat and stored at -80°C in 50 μL aliquots to avoid repeated freeze-thaw cycles.

Following blood collection, the chest cavity was opened and hearts were rapidly excised by cutting the great vessels. Each heart was immediately rinsed in ice-cold phosphate-buffered saline (PBS, pH 7.4) to remove residual blood, gently blotted dry on filter paper, weighed on an analytical balance, and snap-frozen by immersion in liquid nitrogen. Frozen hearts were stored at -80°C in cryovials until processing. For metabolite measurements and Western blotting, frozen hearts were sectioned on a pre-chilled metal block using a razor blade to obtain ~20-30 mg portions from the left ventricular free wall, ensuring standardized sampling across all animals.
