# Supplementary material for: Circulating CD38 shows stronger association with incident heart failure in women despite lower levels: sex differences and kidney-function context
Source: Biol Sex Differ. 2026 Jun 4;17:144. doi: 10.1186/s13293-026-00924-7 (PMC13449966; doi:10.1186/s13293-026-00924-7)
Supplement: Supplementary file 3 [file 13293_2026_924_MOESM2_ESM.docx]

**Supplementary Tables**

## Supplementary Table 1. Baseline Plasma Biomarker Concentrations by Incident Heart Failure Status

| **Biomarker** | **Overall Cohort (N=42,584)** | **No Heart Failure (N=40,984)** | **Heart Failure (N=1,659)** | **P-value** |
| --- | --- | --- | --- | --- |
| Primary Biomarkers (NPX) | | |  |  |
| CD38, mean ± SD | 0.01 ± 0.43 | -0.01 ± 0.43 | 0.19 ± 0.50 | <0.001 |
| CD38, median [IQR] | 0.00 [-0.28, 0.29] | -0.01 [-0.29, 0.28] | 0.19 [-0.10, 0.52] | |
| TGF-β1, mean ± SD | 0.02 ± 0.44 | 0.01 ± 0.44 | 0.14 ± 0.47 | <0.001 |
| TGF-β1, median [IQR] | 0.00 [-0.27, 0.29] | 0.00 [-0.28, 0.28] | 0.14 [-0.15, 0.45] | |
| Secondary Biomarkers | | |  |  |
| NT-proBNP (NPX), mean ± SD | 0.04 ± 1.38 | -0.03 ± 1.37 | 1.08 ± 1.67 | <0.001 |
| NT-proBNP (NPX), median [IQR] | 0.00 [-0.75, 0.79] | -0.03 [-0.77, 0.74] | 1.08 [0.08, 2.42] | |
| IL-6 (NPX), mean ± SD | 0.03 ± 0.77 | -0.02 ± 0.77 | 0.52 ± 0.88 | <0.001 |
| IL-6 (NPX), median [IQR] | 0.00 [-0.46, 0.53] | -0.02 [-0.47, 0.51] | 0.52 [-0.02, 1.13] | |
| ANGPT2 (NPX), mean ± SD | 0.01 ± 0.44 | -0.01 ± 0.44 | 0.24 ± 0.53 | <0.001 |
| ANGPT2 (NPX), median [IQR] | 0.00 [-0.27, 0.28] | -0.01 [-0.28, 0.27] | 0.24 [-0.04, 0.60] | |
| CRP (mg/L), median [IQR] | 1.34 [0.66, 2.78] | 1.31 [0.65, 2.72] | 2.27 [1.09, 4.68] | <0.001 |

Proteomic biomarker values from the Olink platform are reported as Normalized Protein eXpression (NPX) units, a log₂-transformed, plate-normalized relative concentration scale. An NPX value of 0 represents the plate median concentration; a 1-unit increase corresponds to an approximate doubling of protein concentration. Statistical comparisons of means were performed using Student's t-test.

## Supplementary Table 2A. Baseline Characteristics by Menopausal Status (Women Aged 45-60)

| **Characteristic** | **Premenopausal (N=3,033)** | **Postmenopausal (N=8,859)** | **P-value** |
| --- | --- | --- | --- |
| Age, years | 49.8 ± 3.2 | 58.4 ± 4.1 | <0.001 |
| BMI, kg/m² | 27.1 ± 5.2 | 27.8 ± 5.4 | <0.001 |
| Systolic BP, mmHg | 132.4 ± 17.8 | 138.9 ± 19.2 | <0.001 |
| Diastolic BP, mmHg | 81.2 ± 10.1 | 81.8 ± 10.3 | 0.004 |
| Hypertension, n (%) | 892 (29.4) | 4,126 (46.6) | <0.001 |
| Diabetes, n (%) | 42 (1.4) | 198 (2.2) | 0.004 |
| Current smoker, n (%) | 351 (11.6) | 962 (10.9) | 0.26 |
| Biomarkers | |  |  |
| CD38 (NPX) | -0.12 ± 0.41 | 0.08 ± 0.44 | <0.001 |
| NT-proBNP (NPX) | -0.31 ± 1.21 | 0.22 ± 1.42 | <0.001 |
| IL-6 (NPX) | -0.08 ± 0.74 | 0.06 ± 0.79 | <0.001 |
| ANGPT2 (NPX) | -0.08 ± 0.42 | 0.04 ± 0.45 | <0.001 |
| eGFR, mL/min/1.73m² | 92.4 ± 12.1 | 84.6 ± 13.8 | <0.001 |
| Hormones | |  |  |
| Estradiol, pmol/L | 842.3 ± 512.4 | 218.6 ± 198.3 | <0.001 |
| Testosterone, nmol/L | 1.12 ± 0.58 | 1.08 ± 0.62 | 0.002 |
| SHBG, nmol/L | 68.4 ± 32.1 | 58.2 ± 29.8 | <0.001 |
| Free Androgen Index | 0.021 ± 0.018 | 0.024 ± 0.022 | <0.001 |
| IGF-1, nmol/L | 22.4 ± 5.8 | 20.1 ± 5.6 | <0.001 |

Data presented as mean ± SD or n (%). P-values from the t-test or chi-square test comparing premenopausal versus postmenopausal women. NPX, Normalized Protein eXpression; SHBG, sex hormone-binding globulin; FAI, free androgen index; IGF-1, insulin-like growth factor-1.

## Supplementary Table 2B. CD38 Correlations with Circulating Hormones in Both Sexes

| **Hormone** | **Women (r)** | **P-value** | **N** | **Men (r)** | **P-value** | **N** |
| --- | --- | --- | --- | --- | --- | --- |
| Estradiol | -0.051 | 0.0002 | 5,232 | - | - | - |
| Testosterone | -0.006 | 0.41 | 19,117 | -0.06 | <0.001 | 19,276 |
| SHBG | -0.15 | <0.001 | 20,753 | -0.12 | <0.001 | 17,859 |
| Free Androgen Index | 0.08 | <0.001 | 17,312 | 0.08 | <0.001 | 17,744 |
| IGF-1 | -0.09 | <0.001 | 22,948 | 0.04 | <0.001 | 19,374 |

Pearson correlations with CD38 (z-scores). All correlations used complete case analysis. SHBG, sex hormone-binding globulin; FAI, free androgen index; IGF-1, insulin-like growth factor-1. Bold indicates P < 0.05.

## Supplementary Table 2C. CD38 Correlations with Hormones in Women by Menopausal Status

| **Hormone** | **Premenopausal (r)** | **P-value** | **Postmenopausal (r)** | **P-value** |
| --- | --- | --- | --- | --- |
| Estradiol | -0.02 | 0.23 | -0.093 | <0.001 |
| Testosterone | 0.024 | 0.092 | 0.014 | 0.087 |
| SHBG | -0.106 | <0.001 | -0.148 | <0.001 |
| Free Androgen Index | 0.053 | <0.001 | 0.103 | <0.001 |
| IGF-1 | -0.044 | 0.0015 | -0.042 | <0.001 |

Pearson correlations between CD38 (z-scores) and menopausal status in women. Premenopausal N ≈ 3,000; Postmenopausal N ≈ 8,800 (varies by hormone availability). SHBG, sex hormone-binding globulin; FAI, free androgen index; IGF-1, insulin-like growth factor-1. Bold indicates P < 0.05.

## Supplementary Table 2D. Estradiol Mediation Analysis (Women)

| **Effect** | **Estimate** | **95% CI** | **P-value** |
| --- | --- | --- | --- |
| ACME (average causal mediation effect) | −4.2 × 10⁻⁵ | -1.8 × 10⁻⁴ to 1.9 × 10⁻⁴ | 0.554 |
| ADE (average direct effect) | 0.0071 | 5.2 × 10⁻⁴ to 0.016 | 0.030 |
| Total Effect | 0.0071 | 4.6 × 10⁻⁴ to 0.016 | 0.030 |
| Proportion Mediated | -0.006 | -0.05 to 0.03 | 0.57 |

Mediation analysis using the mediation package with 1000 bootstrap simulations. Models adjusted for age, BMI, hypertension, and diabetes. N = 5,232 women with complete estradiol data. ACME, average causal mediation effect; ADE, average direct effect. The proportion mediated was near zero and non-significant (P = 0.57), indicating that circulating estradiol does not mediate the CD38-HF association.

## Supplementary Table 3. Subgroup Analysis of CD38 - Heart Failure Association by Sex

| Subgroup | N | Events | HR (95% CI) | P-value |
| --- | --- | --- | --- | --- |
| Males |  |  |  |  |
| Age < 60 | 10,466 | 231 | 1.09 (0.94-1.26) | 0.27 |
| Age ≥ 60 | 9,056 | 767 | 1.19 (1.10-1.28) | 1.1 × 10⁻⁵ |
| No hypertension | 10,148 | 151 | 1.00 (0.82-1.22) | 0.996 |
| With hypertension | 9,374 | 847 | 1.19 (1.11-1.28) | 1.8 × 10⁻⁶ |
| BMI < 25 (Normal) | 4,935 | 157 | 1.24 (1.04-1.48) | 0.015 |
| BMI 25-30 (Overweight) | 9,736 | 444 | 1.19 (1.08-1.33) | 9.4 × 10⁻⁴ |
| BMI ≥ 30 (Obese) | 4,851 | 397 | 1.13 (1.02-1.25) | 0.017 |
| Females |  |  |  |  |
| Age < 60 | 13,086 | 172 | 1.44 (1.23-1.69) | 9.8 × 10⁻⁶ |
| Age ≥ 60 | 10,036 | 490 | 1.38 (1.25-1.52) | 3.1 × 10⁻¹⁰ |
| No hypertension | 14,547 | 129 | 1.25 (1.01-1.56) | 0.045 |
| With hypertension | 8,575 | 533 | 1.42 (1.30-1.56) | 6.4 × 10⁻¹⁴ |
| BMI < 25 (Normal) | 9,055 | 180 | 1.39 (1.18-1.64) | 6.1 × 10⁻⁵ |
| BMI 25-30 (Overweight) | 8,549 | 205 | 1.44 (1.24-1.68) | 1.5 × 10⁻⁶ |
| BMI ≥ 30 (Obese) | 5,518 | 277 | 1.36 (1.19-1.56) | 6.0 × 10⁻⁶ |

Models adjusted for age, BMI, systolic BP, hypertension, diabetes, hyperlipidemia, and smoking status (excluding the stratification variable). HR, hazard ratio per 1-SD increment in standardized CD38.

## Supplementary Table 4. Model Diagnostics and Predictive Performance

| **Diagnostic Test** | **Males** | **P-value** | **Females** | **P-value** |
| --- | --- | --- | --- | --- |
| Proportional Hazards Test | | |  |  |
| CD38 (Schoenfeld χ²) | 0.32 | 0.57 | 1.21 | 0.27 |
| Global test | 50.35 | 2.6 × 10⁻⁶ | 31.94 | 0.003 |
| Non-linearity Test (RCS) | | |  |  |
| CD38 non-linear (Wald χ²) | 19.41 | 0.0001 | 16.8 | 0.0002 |
| Time-Dependent AUC | | |  |  |
| 5-year: Base model | 0.774 | — | 0.787 | — |
| 5-year: Base + CD38 | 0.776 | — | 0.792 | — |
| 10-year: Base model | 0.795 | — | 0.799 | — |
| 10-year: Base + CD38 | 0.798 | — | 0.806 | — |

Proportional hazards tested using Schoenfeld residuals. Non-linearity tested using restricted cubic splines (RCS) with 4 knots. The base model includes age, sex, BMI, systolic BP, hypertension, diabetes, hyperlipidemia, and smoking. AUC, area under the receiver operating characteristic curve.

## Supplementary Table 5. Sensitivity Analyses Additionally Adjusting for Waist Circumference and Body Fat Percentage

| **Cohort** | **Model** | **Adjustments** | **HR per 1-SD** | **95% CI** |
| --- | --- | --- | --- | --- |
| Overall | M2, BMI only | Age, sex, BMI, hypertension, diabetes, SBP, smoking, alcohol, hyperlipidemia, medications | 1.204 | 1.139–1.272 |
| Overall | M2 + body composition | M2 + waist circumference + body fat percentage | 1.196 | 1.132–1.263 |
| Overall | M3, BMI only | M2 + eGFR | 0.99 | 0.922–1.062 |
| Overall | M3 + body composition | M3 + waist circumference + body fat percentage | 0.986 | 0.919–1.058 |
| Women | M3, BMI only | M2 + eGFR (women) | 1.18 | 1.051–1.324 |
| Women | M3 + body composition | M3 + waist circumference + body fat percentage | 1.166 | 1.038–1.309 |
| Men | M3, BMI only | M2 + eGFR (men) | 0.893 | 0.817–0.976 |
| Men | M3 + body composition | M3 + waist circumference + body fat percentage | 0.895 | 0.819–0.978 |

*Sensitivity analysis restricted to participants with available waist circumference and body fat percentage data (n = 41,908). All P values for body composition-adjusted models are <0.05 for women and men at M3. Results confirm that body composition beyond BMI does not account for the primary findings.*

## Supplementary Table 6. Fine–Gray Competing-Risk Models for the Association Between Circulating CD38 and Incident Heart Failure

| **Cohort** | **Model** | **Adjustments** | **sHR per 1-SD** | **95% CI** | **P value** |
| --- | --- | --- | --- | --- | --- |
| Overall | Fine–Gray, kidney-adjusted | Age, sex, BMI, hypertension, diabetes, SBP, smoking, hyperlipidemia, eGFR | 0.997 | 0.931–1.067 | 0.93 |
| Women | Fine–Gray, kidney-adjusted | Age, BMI, hypertension, diabetes, SBP, smoking, hyperlipidemia, eGFR | 1.189 | 1.067–1.325 | 0.002 |
| Men | Fine–Gray, kidney-adjusted | Age, BMI, hypertension, diabetes, SBP, smoking, hyperlipidemia, eGFR | 0.899 | 0.825–0.979 | 0.015 |

*Death without prior heart failure was treated as the competing event (n = 3,212 competing deaths; 84.8% non-cardiovascular). sHR = subdistribution hazard ratio. Near-exact concordance between Fine–Gray and primary Cox estimates confirms that competing mortality does not materially bias the CD38–HF associations and that the sex-divergent pattern is robust to differential mortality by sex.*

## Supplementary Table 7. Sex-Specific Associations of CD38 and TGF-β1 with Incident Heart Failure

| **Biomarker** | **Subgroup (N, Events)** | **HR (95% CI) per 1-SD** | **P value** | **P-interaction** |
| --- | --- | --- | --- | --- |
| CD38 | Overall (42,584; 1,659) | 1.21 (1.15–1.27) | <0.001 |  |
| CD38 | Female (23,094; 662) | 1.39 (1.29–1.50) | <0.001 | 0.004 |
| CD38 | Male (19,490; 997) | 1.17 (1.09–1.26) | <0.001 |  |
| TGF-β1 | Overall (42,584; 1,659) | 1.15 (1.10–1.21) | <0.001 |  |
| TGF-β1 | Female (23,094; 662) | 1.20 (1.11–1.29) | <0.001 | 0.098 |
| TGF-β1 | Male (19,490; 997) | 1.10 (1.04–1.17) | 0.001 |  |

*Hazard ratios per 1-SD increase in biomarker. Models adjusted for age, sex (overall only), BMI, hypertension, and diabetes (Model 1). P-interaction from multiplicative interaction term (CD38 × sex or TGF-β1 × sex) tested using the z-statistic of the interaction coefficient*

## Supplementary Table 8. Two-Way ANOVA Results for Sex × Treatment Interactions in Murine Studies

| **Outcome** | **Effect** | **SS** | **df** | **MS** | **F** | **P** | **η²** |
| --- | --- | --- | --- | --- | --- | --- | --- |
| Phase 1: Control vs. Isoproterenol | | | | | | | |
| Cardiac CD38 protein | | | | | | | |
|  | Sex | 0.4776 | 1 | 0.3836 | 12.45 | 0.008 | 0.114 |
|  | Treatment | 3.324 | 1 | 3.324 | 86.67 | <0.001 | 0.792 |
|  | Interaction | 0.08045 | 1 | 0.08045 | 2.097 | 0.186 | 0.019 |
|  | Residual | 0.3069 | 8 | 0.03836 |  |  |  |
| Cardiac TGF-β1 protein | | | | | | | |
|  | Sex | 0.0219 | 1 | 0.0219 | 106.6 | <0.001 | 0.064 |
|  | Treatment | 0.3161 | 1 | 0.3161 | 1538 | <0.001 | 0.918 |
|  | Interaction | 0.004705 | 1 | 0.004705 | 22.9 | 0.001 | 0.014 |
|  | Residual | 0.001644 | 8 | 0.000206 |  |  |  |
| Plasma CD38 | | | | | | | |
|  | Sex | 4050 | 1 | 4050 | 17.04 | <0.001 | 0.033 |
|  | Treatment | 111446 | 1 | 111446 | 468.9 | <0.001 | 0.918 |
|  | Interaction | 198.1 | 1 | 198.1 | 0.8334 | 0.37 | 0.002 |
|  | Residual | 5704 | 24 | 237.7 |  |  |  |
| Plasma TGF-β1 | | | | | | | |
|  | Sex | 1.166 | 1 | 1.166 | 1.413 | 0.246 | 0.00013 |
|  | Treatment | 8859 | 1 | 8859 | 10733 | <0.001 | 0.998 |
|  | Interaction | 0.5183 | 1 | 0.5183 | 0.628 | 0.436 | 0.00006 |
|  | Residual | 19.81 | 24 | 0.8254 |  |  |  |
| Plasma ANGPT2 | | | | | | | |
|  | Sex | 7.75 | 1 | 7.75 | 0.047 | 0.83 | 0.00008 |
|  | Treatment | 97441 | 1 | 97441 | 592.6 | <0.001 | 0.958 |
|  | Interaction | 355.8 | 1 | 355.8 | 2.164 | 0.154 | 0.004 |
|  | Residual | 3946 | 24 | 164.4 |  |  |  |
| Plasma Cystatin C | | | | | | | |
|  | Sex | 3.206 | 1 | 3.206 | 0.0922 | 0.764 | 0.0002 |
|  | Treatment | 14569 | 1 | 14569 | 418.8 | <0.001 | 0.888 |
|  | Interaction | 997.2 | 1 | 997.2 | 28.67 | <0.001 | 0.061 |
|  | Residual | 834.8 | 24 | 34.78 |  |  |  |
| Cardiac ATP | | | | | | | |
|  | Sex | 6.969 | 1 | 6.969 | 90.52 | <0.001 | 0.059 |
|  | Treatment | 105.9 | 1 | 105.9 | 1376 | <0.001 | 0.901 |
|  | Interaction | 2.872 | 1 | 2.872 | 37.3 | <0.001 | 0.024 |
|  | Residual | 1.848 | 24 | 0.07699 |  |  |  |
| Cardiac NAD⁺ | | | | | | | |
|  | Sex | 3163 | 1 | 3163 | 12.42 | 0.002 | 0.029 |
|  | Treatment | 100428 | 1 | 100428 | 394.3 | <0.001 | 0.915 |
|  | Interaction | 44.57 | 1 | 44.57 | 0.175 | 0.679 | 0.0004 |
|  | Residual | 6113 | 24 | 254.7 |  |  |  |
| Cardiac pSMAD2/3 | | | | | | | |
|  | Sex | 121.4 | 1 | 121.4 | 0.5779 | 0.456 | 0.004 |
|  | Treatment | 26038 | 1 | 26038 | 123.9 | <0.001 | 0.855 |
|  | Interaction | 96.86 | 1 | 96.86 | 0.4611 | 0.505 | 0.003 |
|  | Residual | 4202 | 20 | 210.1 |  |  |  |
| LV Mass | | | | | | | |
|  | Sex | 0.4135 | 1 | 0.4135 | 0.0057 | 0.941 | 0.00005 |
|  | Treatment | 7,237 | 1 | 7,237 | 99.76 | <0.001 | 0.833 |
|  | Interaction | 285.3 | 1 | 285.3 | 3.933 | 0.065 | 0.033 |
|  | Residual | 1,161 | 16 | 72.54 |  |  |  |
| Ejection Fraction (EF) | | | | | | | |
|  | Sex | 147.4 | 1 | 147.4 | 9.497 | 0.007 | 0.237 |
|  | Treatment | 207 | 1 | 207 | 13.34 | 0.002 | 0.333 |
|  | Interaction | 19.26 | 1 | 19.26 | 1.241 | 0.282 | 0.031 |
|  | Residual | 248.3 | 16 | 15.52 |  |  |  |
| Fractional Shortening (FS) | | | | | | | |
|  | Sex | 190.8 | 1 | 190.8 | 10.8 | 0.005 | 0.195 |
|  | Treatment | 501.8 | 1 | 501.8 | 28.4 | <0.001 | 0.514 |
|  | Interaction | 1.046 | 1 | 1.046 | 0.0592 | 0.811 | 0.001 |
|  | Residual | 282.7 | 16 | 17.67 |  |  |  |
| Phase 2: Pharmacological Inhibition Studies | | | | | | | |
| Plasma CD38 | | | | | | | |
|  | Sex | 1676 | 1 | 1676 | 15.62 | <0.001 | 0.011 |
|  | Treatment | 135833 | 4 | 33958 | 316.5 | <0.001 | 0.927 |
|  | Interaction | 2595 | 4 | 648.7 | 6.047 | <0.001 | 0.018 |
|  | Residual | 6437 | 60 | 107.3 |  |  |  |
| Plasma TGF-β1 | | | | | | | |
|  | Sex | 0.9406 | 1 | 0.9406 | 2.147 | 0.148 | 0.00007 |
|  | Treatment | 12656 | 4 | 3164 | 7224 | <0.001 | 0.998 |
|  | Interaction | 0.9086 | 4 | 0.2272 | 0.5186 | 0.722 | 0.00007 |
|  | Residual | 26.28 | 60 | 0.438 |  |  |  |
| Cardiac pSMAD2/3 | | | | | | | |
|  | Sex | 169.8 | 1 | 169.8 | 1.906 | 0.174 | 0.005 |
|  | Treatment | 29152 | 4 | 7288 | 81.83 | <0.001 | 0.86 |
|  | Interaction | 110.8 | 4 | 27.7 | 0.311 | 0.869 | 0.003 |
|  | Residual | 4453 | 50 | 89.06 |  |  |  |
| Plasma Cystatin C | | | | | | | |
|  | Sex | 1454 | 1 | 1454 | 61.61 | <0.001 | 0.061 |
|  | Treatment | 18635 | 4 | 4659 | 197.4 | <0.001 | 0.786 |
|  | Interaction | 2197 | 4 | 549.2 | 23.28 | <0.001 | 0.093 |
|  | Residual | 1416 | 60 | 23.59 |  |  |  |
| Cardiac ATP | | | | | | | |
|  | Sex | 11.21 | 1 | 11.21 | 217.8 | <0.001 | 0.055 |
|  | Treatment | 186.8 | 4 | 46.71 | 907.8 | <0.001 | 0.91 |
|  | Interaction | 4.305 | 4 | 1.076 | 20.92 | <0.001 | 0.021 |
|  | Residual | 3.087 | 60 | 0.05145 |  |  |  |
| Cardiac NAD⁺ | | | | | | | |
|  | Sex | 3392 | 1 | 3392 | 19.78 | <0.001 | 0.016 |
|  | Treatment | 202949 | 4 | 50737 | 295.9 | <0.001 | 0.933 |
|  | Interaction | 876.7 | 4 | 219.2 | 1.278 | 0.289 | 0.004 |
|  | Residual | 10289 | 60 | 171.5 |  |  |  |
| Plasma ANGPT2 | | | | | | | |
|  | Sex | 1035 | 1 | 1035 | 8.512 | 0.005 | 0.008 |
|  | Treatment | 124922 | 4 | 31230 | 256.9 | <0.001 | 0.914 |
|  | Interaction | 3507 | 4 | 876.6 | 7.21 | <0.001 | 0.026 |
|  | Residual | 7295 | 60 | 121.6 |  |  |  |
| Cardiac Fibrosis & Kidney CD38 Expression | | | | | | | |
| Collagen Volume Fraction | | | | | | | |
|  | Treatment | 0.8378 | 4 | 0.2095 | 307.4 | <0.001 | 0.975 |
|  | Sex | 0.00046 | 1 | 0.00046 | 0.671 | 0.419 | 0.0005 |
|  | Interaction | 0.00036 | 4 | 0.00009 | 0.131 | 0.97 | 0.0004 |
|  | Residual | 0.02044 | 30 | 0.00068 |  |  |  |
| Kidney CD38 mRNA | | | | | | | |
|  | Treatment | 0.2868 | 1 | 0.2868 | 23.47 | <0.001 | 0.607 |
|  | Sex | 2.09×10⁻⁷ | 1 | 2.09×10⁻⁷ | 1.71×10⁻⁵ | 0.997 | 0.0004 |
|  | Interaction | 0.0393 | 1 | 0.0393 | 3.217 | 0.098 | 0.083 |
|  | Residual | 0.1466 | 12 | 0.01222 |  |  |  |

***SS = sum of squares; df = degrees of freedom; MS = mean square; F = F-statistic; η² = eta-squared (effect size). Bold blue P values indicate statistical significance. * Collagen volume fraction from Phase 2 (n = 4/group, total n = 40); kidney CD38 mRNA from Phase 1 (n = 4/group, total n = 16).***
